# Effects of the Chalcogenide Identity in *N*‐Aryl Phenochalcogenazine Photoredox Catalysts

**DOI:** 10.1002/cctc.202200485

**Published:** 2022-07-08

**Authors:** Daniel A. Corbin, Christopher Cremer, Katherine O. Puffer, Brian S. Newell, Frederic W. Patureau, Garret M. Miyake

**Affiliations:** ^1^ Department of Chemistry Colorado State University 200 W. Lake St. Fort Collins Colorado 80523 United States; ^2^ Institute of Organic Chemistry RWTH Aachen University Landoltweg 1 52074 Aachen Germany; ^3^ Analytical Resources Core, Materials and Molecular Analysis Center Colorado State University 200 W. Lake St. Fort Collins Colorado 80523 United States

**Keywords:** chalcogens, O-ATRP, organocatalysis, photocatalysis, photooxidation, polymerization

## Abstract

Phenochalcogenazines such as phenoxazines and phenothiazines have been widely employed as photoredox catalysts (PCs) in small molecule and polymer synthesis. However, the effect of the chalcogenide in these catalysts has not been fully investigated. In this work, a series of four phenochalcogenazines is synthesized to understand how the chalcogenide impacts catalyst properties and performance. Increasing the size of the chalcogenide is found to distort the PC structure, ultimately impacting the properties of each PC. For example, larger chalcogenides destabilize the PC radical cation, possibly resulting in catalyst degradation. In addition, PCs with larger chalcogenides experience increased reorganization during electron transfer, leading to slower electron transfer. Ultimately, catalyst performance is evaluated in organocatalyzed atom transfer radical polymerization and a photooxidation reaction for C(sp^2^)−N coupling. Results from these experiments highlight that a balance of PC properties is most beneficial for catalysis, including a long‐lived excited state, a stable radical cation, and a low reorganization energy.

## Graphical abstract

**Atom transfer**: This work explores the effect of the chalcogenide identity in phenochalcogenazine photocatalysts for small molecule photooxidations and polymer synthesis by organocatalyzed atom transfer radical polymerization. Notably, increasing the size of the chalcogenide in these photocatalysts results in greater reorganization energy penalties during electron transfer as well as destabilized radical cations, slowing electron transfer and possibly leading to photocatalyst degradation, respectively.

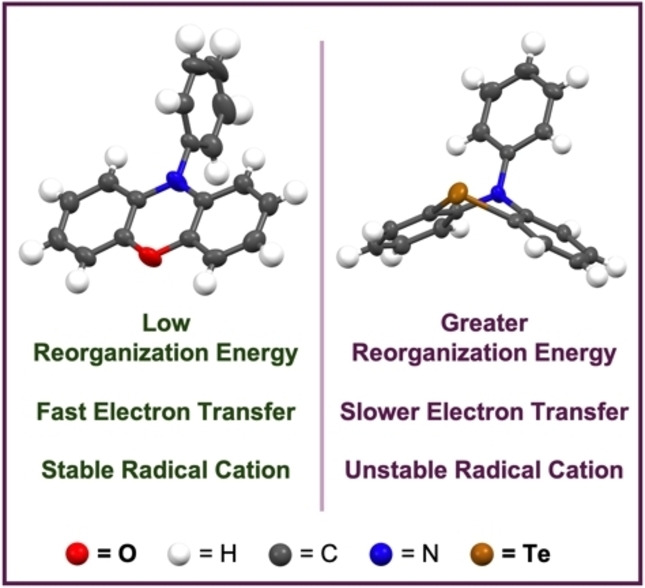

## Introduction

**Figure 1:**
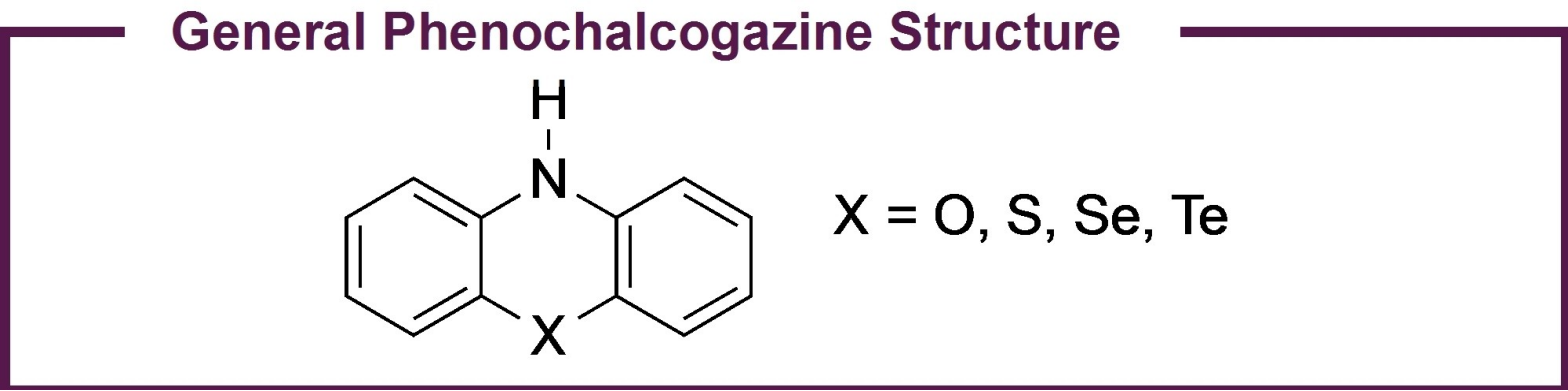
General structure of phenochalcogenazines.

For over a century, phenochalcogenazines (Figure 1) – primarily phenoxazines and phenothiazines – have attracted the interest of the synthetic chemistry community.^[1]^ Early interest in this class of molecules stemmed from observations that many phenochalcogenazines could act as photosensitizers, yielding new pathways to interesting chemical transformations.^[2]^ As a result of these early works, subsequent research efforts continued to explore the reactivity of phenochalcogenazines as photosensitizers and photoredox catalysts (PCs).^[3]^ In recent years, this class of molecules has found numerous applications in various small molecule and polymer syntheses.[^4^, ^5^, ^6^, ^7^, ^8^] However, perhaps the most significant contributor to the popularity of these molecules as PCs in recent times has been the application of phenothiazines and phenoxazines in organocatalyzed atom transfer radical polymerization (O‐ATRP).[^6^, ^8^]

**Figure 2:**
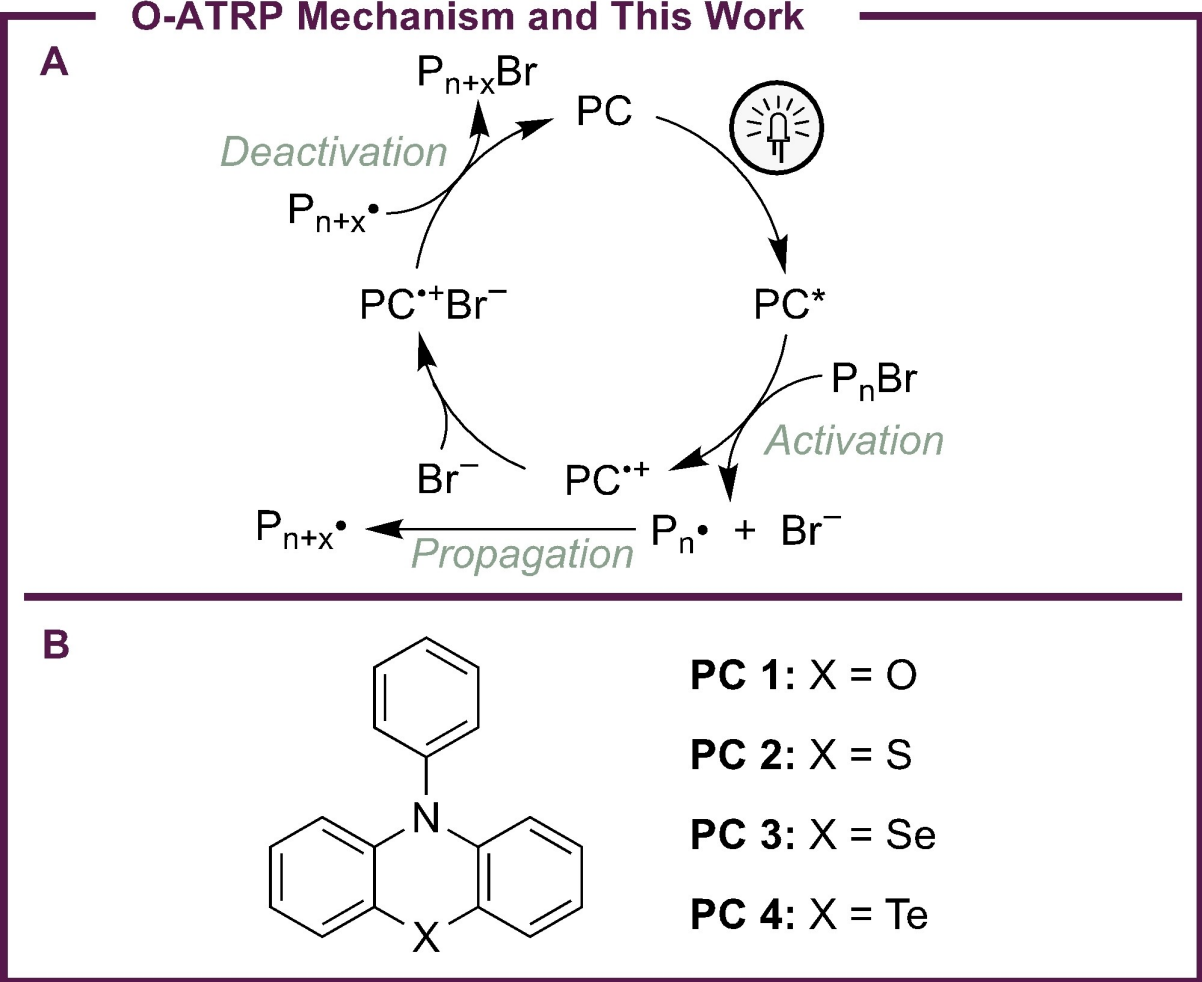
Mechanism of O‐ATRP (a) and structures of PCs investigated in this work (b).

O‐ATRP is a controlled radical polymerization strategy employing organic PCs for the synthesis of well‐defined polymers with precise structures. In O‐ATRP, control of polymer structure is achieved through a reversible activation/deactivation mechanism (Figure 2a), which maintains a low concentration of propagating polymer radicals (P_n_^⋅^) to minimize biomolecular radical side reactions caused by these intermediates. In order for a PC to successfully mediate this process, several catalyst properties are necessary, including a sufficiently reducing excited state to reduce a polymer alkyl bromide bond [*E*°(C−Br/C−Br^⋅−^) ∼−0.8 to −0.6 V vs. saturated calomel electrode (SCE)], an excited state lifetime (*τ*) long enough to engage in bimolecular reactions (typically nanoseconds or longer), and chemical reversibility so the PC can be regenerated after electron transfer (ET).^[9]^

Following early O‐ATRP reports, the superior catalytic properties of 10‐phenyl phenothiazine^[6]^ over perylene^[10]^ motivated the development of new phenochalcogenazine PCs, including numerous phenothiazines[^11^, ^12^] and phenoxazines.[^7^, ^13^, ^14^] In particular, significant research efforts were devoted to tuning the yield of PC triplet excited states (^3^PC*),[^7^, ^12^, ^15^, ^16^] which may benefit catalysis due to their longer lifetimes than singlet excited states (^1^PC*) by making them more likely to engage in catalysis.[^17^, ^18^, ^19^] It has been hypothesized that tuning the identity of the chalcogenide in these PCs could serve as a useful strategy for increasing the yield of ^3^PC*. Introducing heavy atoms such as Se and Te into the PC structure could increase the yield of ^3^PC* through the heavy atom effect, which increases spin‐orbit coupling to encourage intersystem crossing (ISC) from the singlet to the triplet excited state manifold.[^20^, ^21^]

In addition to influencing the yield of ^3^PC*, the identity of the chalcogenide in phenochalcogenazine PCs could also impact reorganization of the PC during ET. Previous work has investigated this possibility using density functional theory (DFT), which suggested the increase in chalcogenide size from O to S causes structural distortions in phenothiazine PCs relative to phenoxazines. These distortions could introduce greater reorganization of the PC during ET, in turn lowering the rate of ET and hindering catalysis. Supporting these findings, when 1‐naphthyl‐10‐phenoxazine and 1‐naphthyl‐10‐phenothiazine were compared as PCs in O‐ATRP, the phenoxazine PC showed superior polymerization performance despite having otherwise similar properties to the phenothiazine.^[7]^

Finally, the identity of the chalcogenide could also impact the reactivity of phenochalcogenazine radical cations, which have recently been investigated for their ability to perform challenging excited‐state oxidations. Early work by Wasielewski and coworkers showed phenothiazine could act as an excited‐state photooxidant when covalently tethered to electron‐donating moieties, generating interest in the exploration of these molecules as potent photooxidants.^[22]^ In work by Rombach and Wagenknecht, 10‐phenyl phenothiazine was explored for its ability to mediate the pentafluorosulfanylation of styrenes. The radical cation of this PC was proposed to act as an excited state photooxidant in this mechanism, oxidizing various styrenes to initiate radical addition to the alkene.[^5^, ^23^] Further, recent work by Wickens and coworkers revealed this same radical cation can serve as a potent photooxidant to mediate C(sp^2^)−N bond forming reactions between *N*‐containing heterocycles and benzene.^[24]^ Motivated by these reports, we wondered if other phenochalcogenazines would exhibit similar, if not improved, photooxidation behavior, since the identity of the chalcogenide could impact several radical cation properties, such as oxidation potential, excited state lifetime, or chemical stability.

For these reasons, our groups have been interested in investigating phenochalcogenazine PCs for O‐ATRP and other photoredox reactions. Enabled by recent synthetic advancements which have facilitated the synthesis of selenazines and tellurizines,^[25]^ this work aims to understand how the chalcogenide identity in these PCs impacts their catalytic properties and PC performance. Specifically, this work investigates a series of four phenochalcogenazines which differ only in the identity of their chalcogenides (Figure 2b). By investigating the effect of this atom, structural effects are revealed that can significantly impact PC stability and ET rates, ultimately impacting the ability of these PCs to perform as effective catalysts in O‐ATRP and other chemical transformations.

## Results and Discussion

**Figure 3:**
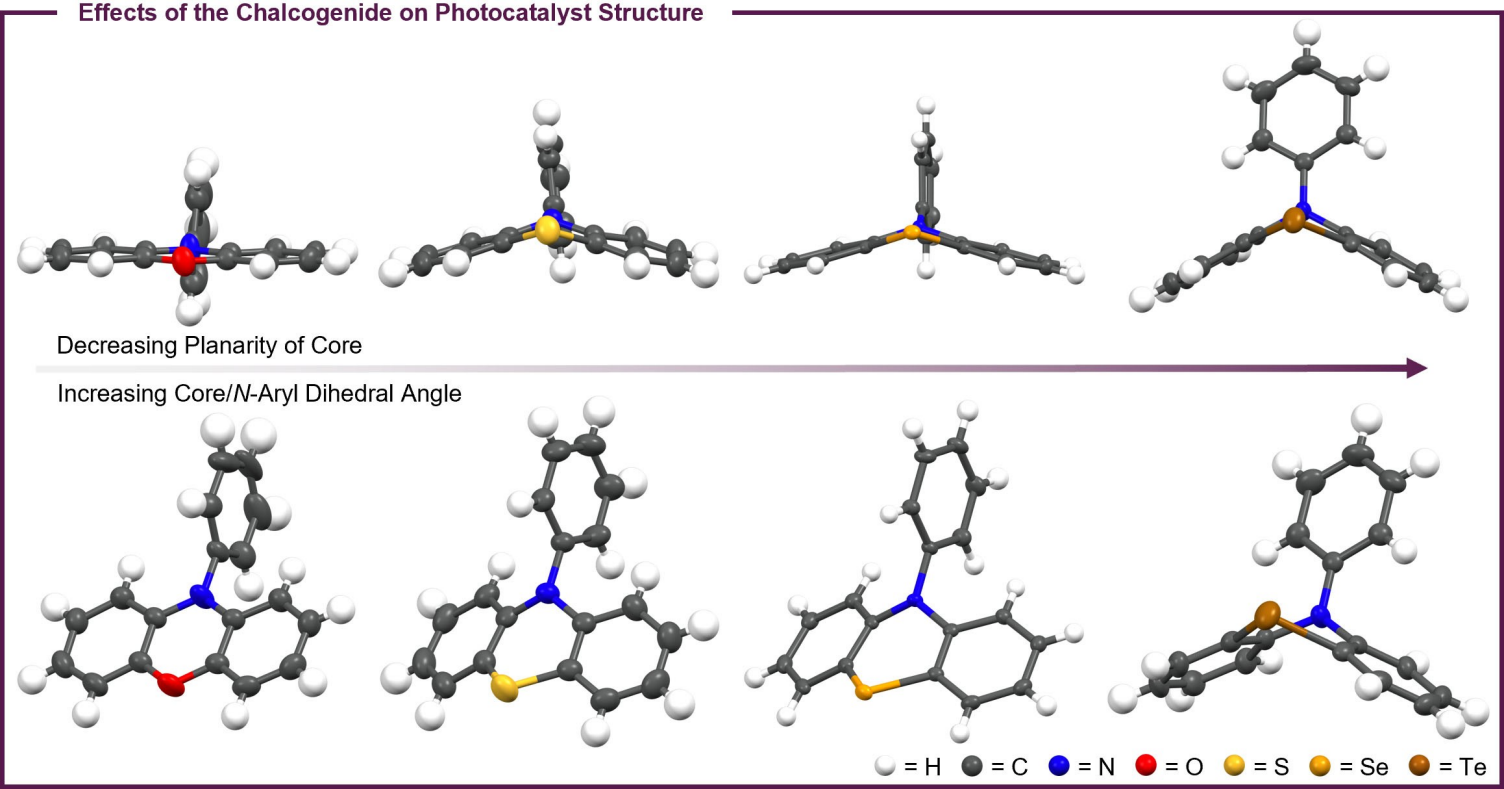
Crystal structures of PCs **1**–**4** showing that increasing the size of the chalcogenide decreases the planarity of the PC core (top) and increases the dihedral angle between the PC core and *N*‐aryl group (bottom).

One of the major effects expected upon altering the identity of the chalcogenide in PCs **1**–**4** is a distortion of the PC structure due to the increasing size of the chalcogenide going down the group. To investigate these possible structural distortions, crystal structures of PCs **1**–**4** were collected (Figure 3). Upon examination of these data, two trends are present. As the size of the chalcogenide increases, the planarity of the PC core decreases to accommodate the larger atom in the core. In addition, the dihedral angle between the PC core and *N*‐aryl group increases with increasing chalcogenide size (1.24° for **1**, 5.93° for **2**, 17.44° for **3**, and 75.89° for **4**). As a result, the central ring of the PC core goes from a planar geometry in **1** to a boat‐shaped geometry in **4**, placing the *N*‐aryl group perpendicular to the core of **4** rather than in the plane of the core as is seen in **1**.

**Figure 4:**
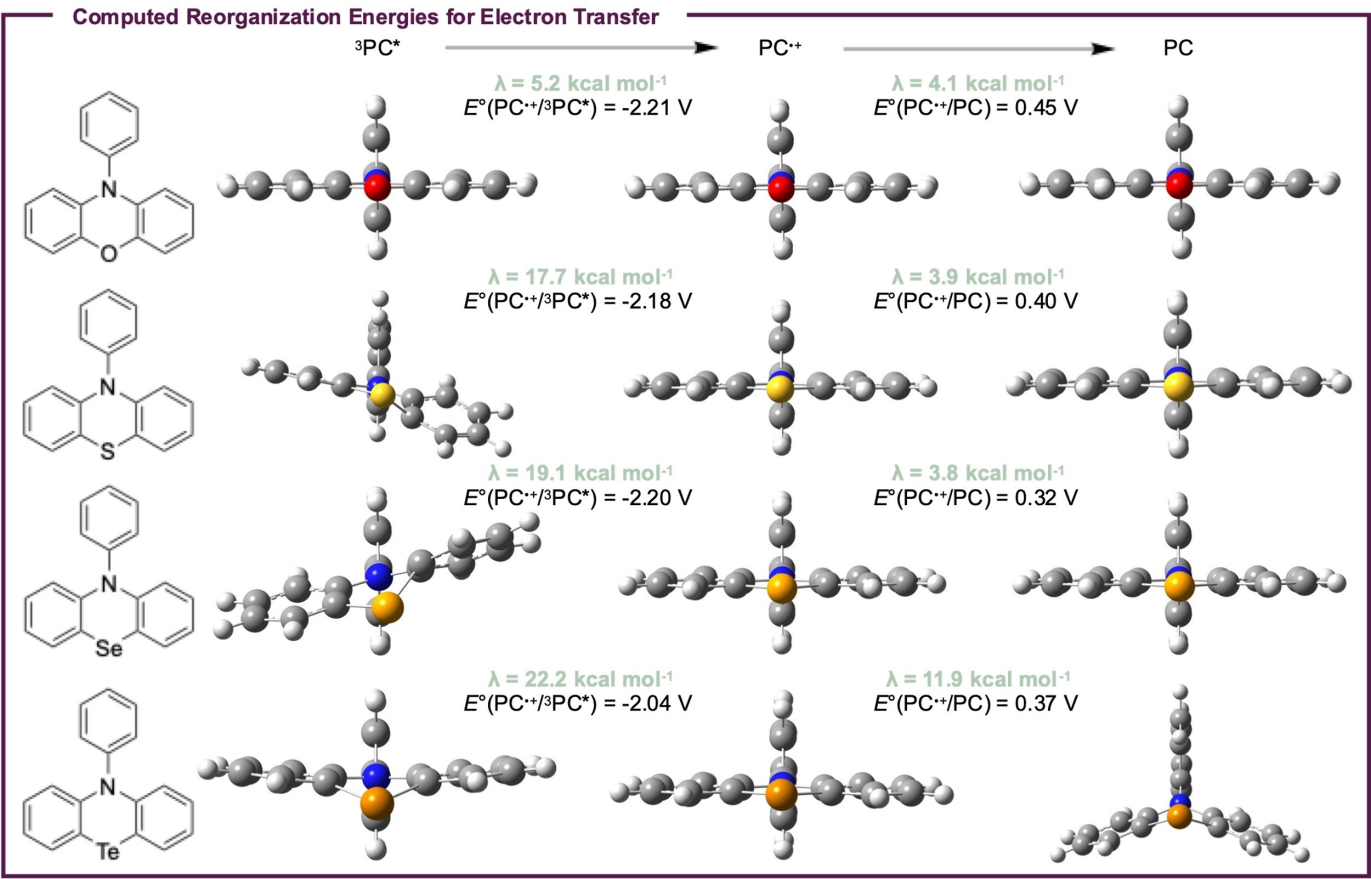
Computed structures and reorganization energies for ET from the PC triplet excited states (^3^PC* to PC^⋅+^) and to the PC radical cations (PC^⋅+^ to PC, see *Computational Details and Data* in the *Supporting Information*).

To investigate the effect these structural distortions could have on catalysis, density functional theory (DFT) was employed. The structures of three PC states – the triplet excited state (^3^PC*), the radical cation (PC^⋅+^), and the ground state (PC) – were computed at uM06/LANL2DZ to analyze reorganization energies (λ) relevant to ET during catalysis (Figure 4). Importantly, analysis of the DFT predicted PC structures reveals qualitatively similar trends in core geometries and core/*N*‐aryl dihedral angles, although these predicted trends are not quite as dramatic as those observed experimentally (Figures S76–S79). This disagreement between DFT and the experimentally determined crystal structures could be due to the relatively low level of theory used for these calculations, which was necessitated by an incompatibility between the Te atom and the typical basis sets[^7^, ^13^] used for these calculations. Alternatively, it is also possible that the DFT predicted structures are more representative of the solution structures of PCs **1**–**4**, whereas the differences seen in the crystal structures arise from the packing of these molecules in the solid state.

Regardless of the reasons for these disparities, these structural distortions are predicted to increase λ going from **1** to **4**, especially for ET from ^3^PC* (λ=5.2 kcal mol^−1^ for **1**, 17.7 kcal mol^−1^ for **2**, 19.1 kcal mol^−1^ for **3**, 22.2 kcal mol^−1^ for **4**). Practically, these increases in λ could lower rates of ET during catalysis, although this possibility will be discussed further in a subsequent section. With regard to the reduction of PC^⋅+^ to PC, a small effect on λ is also observed, although it is only significant for PC **4** (λ=11.9 kcal mol^−1^ vs. λ ∼4 kcal mol^−1^ for **1**–**3**). This effect appears to be caused predominately by distortions in the structure of the PC ground state, since all four PC^⋅+^ structures are predicted to have similar geometries. As such, it is possible the significant PC distortions observed experimentally for **1**–**4** may cause more significant λ values for PC^⋅+^ reduction than are predicted by DFT, assuming the crystal structures of **1**–**4** are representative of their solution phase structures.

Another area where structural distortions due to changes in the size of the chalcogenide could be impactful is in the photophysical and electrochemical properties of these compounds. Table 1 provides data related the absorption and emission of PCs **1**–**4** in *N,N*‐dimethylacetamide (DMAc), which shows a blue‐shift of the absorption (*λ*_max,abs_) of each PC with increasing chalcogenide size. This effect can be rationalized by the decreasing planarity of the PC core with increasing chalcogenide size, which is expected to decrease the degree of conjugation across the molecule and in turn increase its energy of absorption.

**Table 1:** UV‐Visible absorption and emission data for PCs **1**–**4**.

PC	*λ*_max,abs_ [nm]^[a]^	*ϵ*_max_ [M^−1^ cm^−1^]^[a]^	*λ*_max,fluor_ [nm]^[a]^	*λ*_max,phos_ [nm]^[b]^
**1**	322	9000	391	490
**2**	317	3600	444	533
**3**	310^[c]^	4000	419^[d]^	507
**4**	290^[c]^	10,600	418^[d]^	504

[a] In DMAc. [b] In DMF at 77 K, excited at 355 nm. [c] Determination of *λ*_max,abs_ was complicated by overlap with another absorption band, so this value was estimated (see *Supporting Information* for more details). [d] Excited at 355 nm.

Unfortunately, no other clear trends emerged for this series of compounds. When the molar absorptivities (ϵ_max_) of PCs **1**–**4** were measured, a decrease in ϵ_max_ was seen going from **1** (9,000 M^−1^ cm^−1^) to **2** (3,600 M^−1^ cm^−1^), but an increase in ϵ_max_ was observed from **2** to **4** (ϵ_max_=4,000 M^−1^ cm^−1^ for **3**; 10,600 M^−1^ cm^−1^ for **4**). Similarly, when the emission spectra of PCs **1**–**4** were measured, both the wavelength of maximum fluorescence (λ_max,fluor_) and phosphorescence (λ_max,phos_) increased from **1** (λ_max,fluor_=391 nm) to **2** (444 nm), but decreased from **2** to **4** (419 nm for **3**; 418 nm for **4**).

Using these data in combination with electrochemical characterization, the ground state and excited state redox properties of PCs **1**–**4** in DMAc were probed as well (Table 2). From the emission data in Table 1, the singlet and triplet excited state energies (*E*_S1,exp_ and *E*_T1,exp_, respectively) were obtained. In addition, cyclic voltammetry was employed to estimate the oxidation potential of each compounds’ radical cations [*E*_1/2_(PC^⋅+^/PC)∼*E*°(PC^⋅+^/PC)], although the identity of the chalcogenide seems to have a negligible impact on this property. By then subtracting the excited state energies from *E*_1/2_(PC^⋅+^/PC), estimates for the singlet and triplet excited state reduction potentials were obtained [*E*°_exp_(PC^⋅+^/^1^PC*) and *E*°_exp_(PC^⋅+^/^3^PC*), respectively]. Since there is almost no variation in *E*_1/2_(PC^⋅+^/PC), both *E*°_exp_(PC^⋅+^/^1^PC*) and *E*°_exp_(PC^⋅+^/^3^PC*) follow similar trends to those seen in the excited state energies (i. e. decreasing in magnitude from **1** to **2**, and then increasing from **2** to **4**).

**Table 2:** Photophysical and electrochemical properties of PCs **1**–**4**.

PC	*E*_S1,exp_ [eV]^[a]^	*E*_T1,exp_ [eV]^[b]^	*E*_T1,calc_ [eV]^[c]^	*E*_1/2_(PC^⋅+^/PC) [V vs. SCE]	*E*°_calc_(PC^⋅+^/PC) [V vs. SCE]^[c]^	*E*°_exp_(PC^⋅+^/^1^PC*) [V vs. SCE]^[d]^	*E*°_exp_(PC^⋅+^/^3^PC*) [V vs. SCE]^[d]^	*E*°_calc_(PC^⋅+^/^3^PC*) [V vs. SCE]^[c]^
**1**	3.17	2.53	2.66	0.66	0.45	−2.51	−1.87	−2.21
**2**	2.79	2.33	2.57	0.66	0.40	−2.13	−1.67	−2.18
**3**	2.96	2.45	2.52	0.67^[e]^	0.32	−2.29	−1.78	−2.20
**4**	2.97	2.46	2.41	0.63^[e]^	0.37	−2.34	−1.83	−2.04

[a] Determined from the emission maximum in DMAc. [b] Determined from the emission maximum in DMF at 77 K. [c] Computed by DFT at uM06/LANL2DZ/CPCM−H_2_O. [d] *E*°_exp_=*E*_1/2_−*E*_0,0_, where *E*_0,0_=*E*_S1_ or *E*_T1_. [e] Estimated from the *E*_1/2_ measured in DCM versus the ferrocene/ferrocenium redox couple (see *Electrochemical Characterization* in the *Supporting Information*).

**Figure 5:**
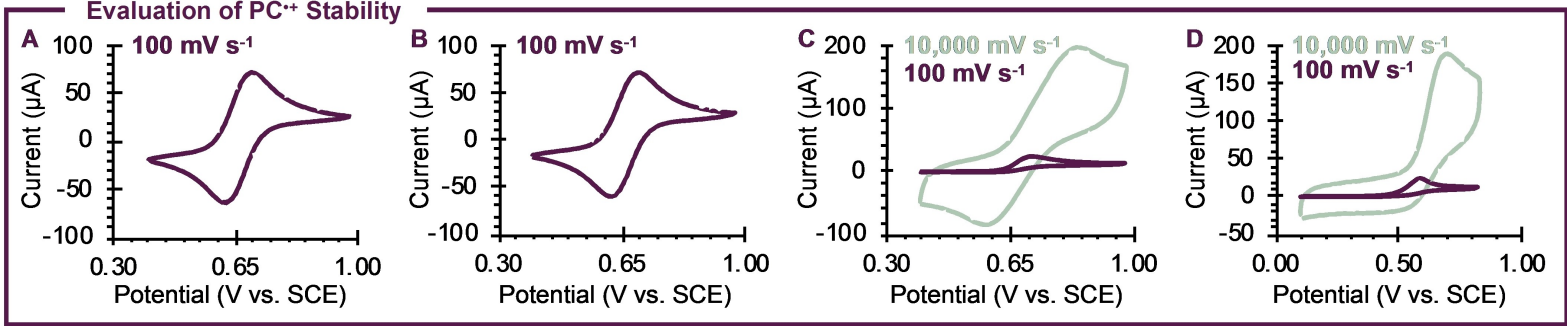
Cyclic voltammograms for PCs **1** (a), **2** (b), **3** (c), and **4** (d) in DMAc showing a decrease in electrochemical reversibility as a function of chalcogenide identity (O ≈ S > Se > Te).

Where a trend is observed is in the stability of the radical cations of PC **1**–**4**. It has been well documented that the reversibility of a compound's cyclic voltammogram can give insight into the chemical stability of the oxidized or reduced species formed during electrochemical experiments.^[26]^ In the cases of PCs **1** and **2**, excellent reversibility is observed in DMAc, both qualitatively (Figures 5a and b) and quantitatively (i_ac/pc_=0.96 for **1** and 0.99 for **2**, where i_ac/pc_=1.00 indicates perfect reversibility; see Figures S47 and S48 for more details). Instead, PCs **3** and **4** show poor reversibility under the same conditions (Figures 5c and d, purple traces), indicating possible decomposition of the radical cations formed upon oxidation of **3** and **4**. In the case of **3**, increasing the scan rate from 100 mV s^−1^ to 10,000 mV s^−1^ improves the reversibility of this system (Figure 5c, green), yielding a value of i_ac/pc_=1.68 (Figure S49). However, the reversibility of this compound is still less than ideal. Instead, performing the same experiment with PC **4** gives no improvements in reversibility (Figure 5d), indicating that increasing the size of the chalcogenide may destabilize PC^⋅+^.

Since *E*_1/2_(PC^⋅+^/PC) remains relatively unchanged for PCs **1**–**4**, it is unlikely that the oxidation potential of PC^⋅+^ contributes to this decrease in stability. Instead, it is possible that the structural distortions caused by increasing the size of the chalcogenide contribute to the decreased stability of the radical cations of **3** and **4**. Similar effects have been observed previously for dihydroacridine PCs,[^27^, ^28^] where the chalcogenide is replaced by a quaternary carbon. In this catalyst family, radical cation stability was ultimately enabled by adding substituents to the 3‐ and 7‐ positions of the PC core, which inhibit reactivity from these core positions in PC^⋅+^.^[28]^ It is possible a similar substitution strategy could be beneficial for **3** and **4**, although further investigation of this approach is necessary.

**Figure 6:**
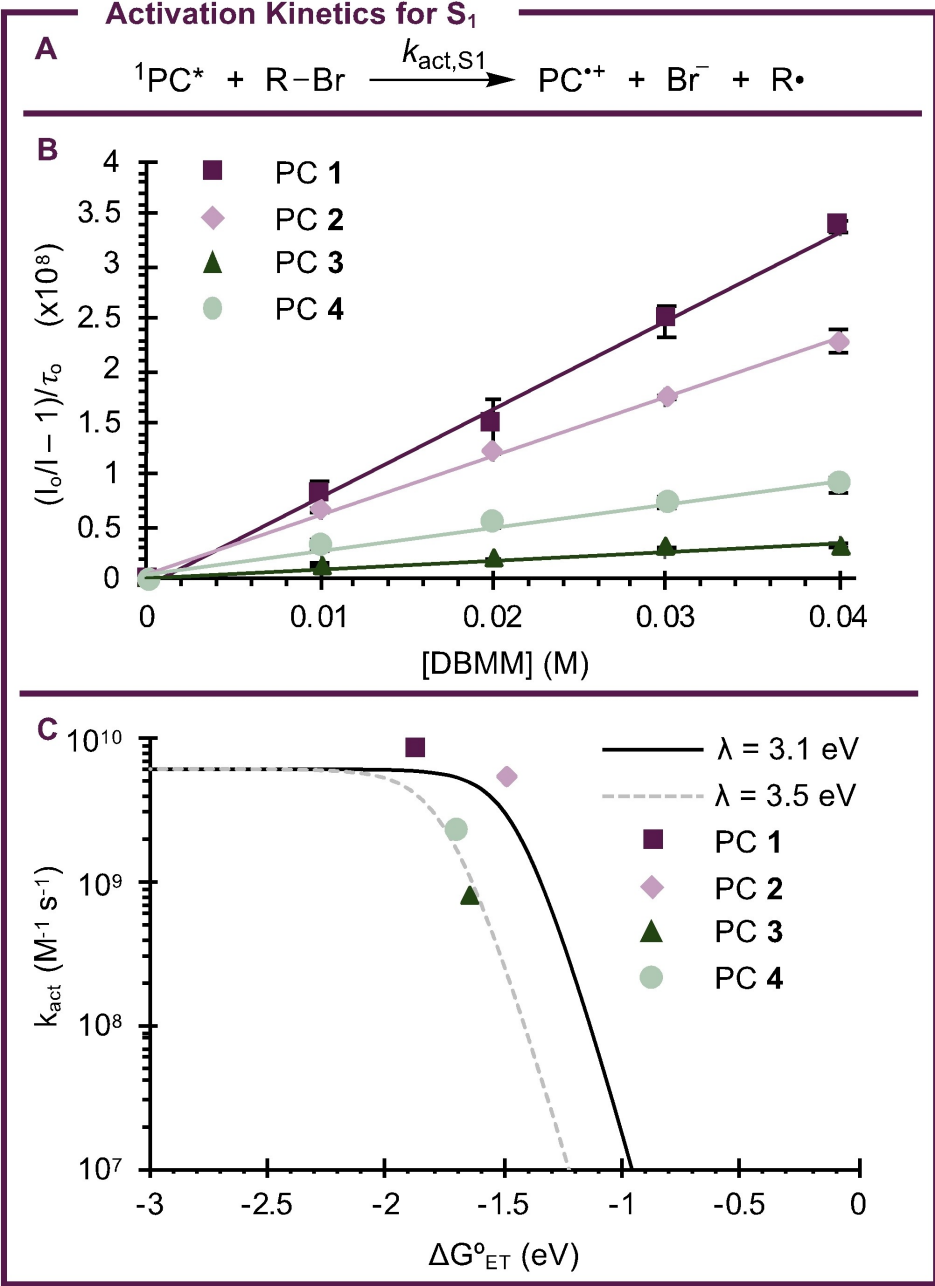
(a) Scheme for the measurement of *k*_act,S1_ by Stern‐Volmer fluorescence quenching (see *Steady State Emission Spectroscopy* in the *Supporting Information*). (b) Activation kinetics for the S_1_ excited state obtained by steady state Stern‐Volmer quenching. (c) Analysis of activation kinetics by Marcus Theory with two different values for the reorganization energy (λ).

To understand how the chalcogenide identity ultimately impacts the reactivity of PCs **1**–**4**, the ability of these compounds to perform as photoredox catalysts in O‐ATRP was investigated next. In the case of **4**, we note the metallic character of Te may be counter to the definition of “organocatalyzed.” However, the performance of this PC in O‐ATRP is still of interest as it relates to understanding the impact of the chalcogenide on PC properties. Initially, Stern‐Volmer fluorescence quenching was employed to probe whether the excited states of PCs **1**–**4** could mediate activation in O‐ATRP by reduction of an alkyl−bromide bond (Figure 6a). While Stern‐Volmer quenching can occur through either ET or energy transfer, ET is more likely on the basis of spectral overlap – or lack thereof – between the PC emission and quencher absorption, where the quencher chosen here is diethyl‐2‐bromo‐2‐methylmalonate (DBMM). Further, all of the PCs used in this work exhibit a sufficient thermodynamic driving force for electron transfer to DBMM, this making electron transfer the most likely mechanism of fluorescence quenching.^[17]^ Importantly, since these experiments followed the fluorescence of PCs **1**–**4**, activation by the singlet excited state (S1) was likely measured exclusively. While it is expected that the triplet excited state (T1) of each PC can also contribute to activation,[^19^, ^29^] measurement of activation from S1 provides useful insight into this reactivity and answers to questions regarding activation.

To measure Stern‐Volmer quenching under steady state conditions, a solution of each PC was prepared in DMAc and its fluorescence spectrum measured. Additional PC solutions were then prepared with excess quantities of a quenching molecule (Q), and the fluorescence spectra of these solutions were again measured. In this case, DBMM was chosen as the quencher given its usage as an initiator in O‐ATRP. In the presence of DBMM, it was anticipated the intensity of fluorescence would be decreased relative to the solution of pure PC at the same concentration, since the reaction between PC* and DBMM should decrease the [PC*] available to undergo fluorescence. As such, these data were analyzed according to Equation 1 (Figure 6b), which relates the change in fluorescence intensity with (*I*) and without (*I*°) quencher present to the rate constant (*k*_q_) for the quenching reaction (i. e. activation, where *k*_q_=*k*_act,S1_), the lifetime of the singlet excited state (*τ*_o_=*τ*_S1_), and the [Q]=[DBMM].

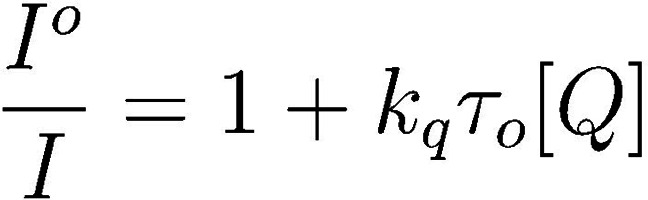




To obtain the lifetime of the singlet excited states of **1**–**4**, time correlated single photon counting (TCSPC) was employed and yielded lifetimes of about 3 ns for **1** and **2**, and lifetimes of about 9 ns for **3** and **4** (Table 3). In addition, the quantum yield of fluorescence was measured for each PC, although this property should not impact Stern‐Volmer quenching since pseudo‐first order kinetics are assumed in the derivation of the Stern Volmer equation (Equation 1) and the quencher (i. e. DBMM) is present in large excess (i. e. 100 or more equivalents) relative to the PC.^[30]^

**Table 3:** Properties of the S_1_ excited state for PCs **1**–**4** related to activation in O‐ATRP.

PC	*Φ*_fluor._ [%]	*τ*_S1_ [ns]^[a]^	ΔG°_ET,S1_ [Ev]^[b]^	*k*_act,S1_ [x10^9^ M^−1^ s^−1^]^[c]^
**1**	6.6	2.95±0.03	−1.87	8.4±0.1
**2**	1.4	3.15±0.08	−1.49	5.6±0.2
**3**	0.3	9.10±0.30	−1.65	0.81±0.05
**4**	4.3	9.33±0.27	−1.70	2.2±0.1

[a] Measured by TCSPC in DMAc, values are an average of three separate measurements. [b] In DMAc. [c] Measured in triplicate by steady state Stern‐Volmer quenching.

With these properties and considerations in mind, rate constants for activation from the singlet excited states of **1**–**4** (*k*_act,S1_) were measured and are reported in Table 3. The values for *k*_act,S1_ were generally near the diffusion limit and ranged from 8.4×10^9^ M^−1^ s^−1^ for **1** to 8.1×10^8^ M^−1^ s^−1^ for **3**. To understand the factors influencing *k*_act,S1_, these data were analyzed according to Marcus‐Saveánt theory as recently discussed by Lattke *et al*. for phenoxazine based PCs.^[17]^ In their work, Equation 2 was shown to describe the relationship between *k*_act_ and the driving force for ET during activation [ΔG°_ET_∼*E*°(PC^⋅+^/PC)−*E*°(DBMM/DBMM^⋅−^)] for this class of PCs, where *k*_diff_ is the rate constant for diffusion and is estimated as 6.1×10^9^ M^−1^ s^−1^; *K*_d_ describes the equilibrium formation of the encounter complex between PC* and DBMM prior to ET and is estimated as 0.55 M^−1^; *h* is the Planck constant (6.58×10^−16^ eV s); *K*_B_ is the Boltzmann constant (8.62×10^−5^ eV K^−1^); T is the absolute temperature (293.15 K); and λ is the reorganization energy described by Marcus‐Saveánt theory.^[17]^


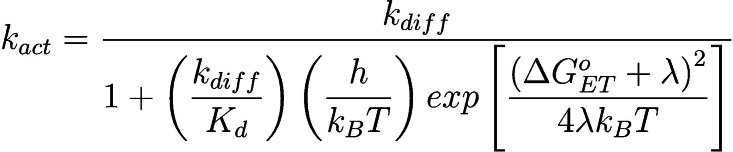




Interestingly, this analysis revealed that, in agreement with prior work for which λ=3.1 eV (Figure 6c, black line),^[17]^ *k*_act,S1_ for **1** and **2** is largely dependent upon the value of ΔG°_ET_. However, the data obtained for **3** and **4** showed significant deviation from this prior work. By changing the value for λ from 3.1 to 3.5 eV (Figure 6c, grey dashed line), better agreement was obtained between the experimental data for **3** and **4** and Equation 2, suggesting PCs **3** and **4** may experience a greater reorganization energy penalty during this ET process. Excitingly, this conclusion is consistent with our earlier predictions made based on the crystal structures of **1**–**4** and DFT calculations suggesting an increase in λ with increasing chalcogenide size. Additionally, this result may explain why a decrease in *k*_act,S1_ is observed from **2** (5.6×10^9^ M^−1^ s^−1^) to **3** (8.1×10^8^ M^−1^ s^−1^), even though **3** exhibits a larger ΔG°_ET_ than **2** (−1.65 eV vs. −1.49 eV, respectively). While ΔG°_ET_ has a significant influence on *k*_act,S1_, the effect of reorganization during ET cannot be neglected for PCs with larger chalcogenides in the PC core.

With these results in mind, the ability of PCs **1**–**4** to mediate O‐ATRP of methyl methacrylate (MMA) was investigated (Table 4). The conditions for these reactions were chosen based on previously reported conditions for phenoxazine PCs.^[7]^ Under these conditions, PCs **1** and **2** showed the best performance in O‐ATRP, as measured by their ability to produce poly(methyl methacrylate) (PMMA) with low dispersity (*Ð*≤1.5) and near quantitative initiator efficiency (I* ∼100 %). By contrast, PCs **3** and **4** produced PMMA with higher *Ð* (1.92 for **3** and 1.66 for **4**) and significantly lower I* (59 % for **3** and 18 % for **4**). Further, when the polymerization performance of PCs **1**–**4** was tracked over the course of the reaction (Figures S57–S60), PC **2** showed the best *Ð* and I* over time, indicating consistent polymerization control throughout the reaction rather than only at the end as in the case of **1**. Polymerizations performed using PCs **1**–**4** without light resulted in no conversion (Table S11).

**Table 4:** Polymerization results from the O‐ATRP of methyl methacrylate using PCs **1**–**4**.

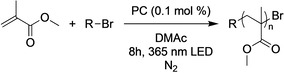					
PC	Conv. [%]^[a]^	*M*_n,theo_ [kDa]	*M*_n,exp_ [kDa]^[b]^	*Ð* ^[b]^	I* [%]^[c]^
**1**	80	8.25	8.44	1.53	98
**2**	75	7.77	8.97	1.44	87
**3**	48	5.02	8.47	1.92	59
**4**	64	6.68	37.4	1.66	18

[a] Conversion of monomer to polymer at 8 h. [b] Determined by gel permeation chromatography. [c] Initiator efficiency (I*)=(*M*_n, theo_/*M*_n, exp_) ⋅ 100 %. See the *Experimental* section for more details.

Together, these data give insight into several important PC design features. As has previously been hypothesized in O‐ATRP,[^17^, ^19^, ^31^] increasing the yield of ^3^PC* by increasing intersystem crossing (ISC) should benefit polymerization control by creating a long‐lived lived excited state that can easily mediate activation. This property is likely the reason PC **2** exhibits better performance than PC **1** in O‐ATRP, since the S atom in **2** should increase ISC by the heavy atom effect[^20^, ^21^] relative to **1**.

At the onset of this work, it was believed that replacing S with Se in **3** or Te in **4** would further increase ISC and improve PC performance in O‐ATRP, but that is not the case. It is possible that Se and Te do not significantly impact ISC relative to S, although this effect should not decrease polymerization control as observed for **3** and **4**. Instead, a likely explanation for this trend is that **3** and **4** are capable of mediating activation in O‐ATRP, but deactivation with these PCs is ineffective. For this reason, conversion of the monomer to polymer is observed (i. e. activation has occurred), but the polymer produced exhibits *Ð* >1.5 and I*≪100 % (i. e. deactivation was poor).

While the cause of poor deactivation with **3** and **4** is currently unknown, it may be due to the poor PC^⋅+^ stability of these PCs in DMAc. Alternatively, since **3** and **4** exhibit slower activation due to reorganization, it is possible that the buildup of PC^⋅+^ during early polymerization times is slower, which in turn may delay effective deactivation and lead to poor polymerization control. In either case, these results suggest a balance of catalyst properties is necessary to achieve optimal control in O‐ATRP. While changing the chalcogenide may lead to some improvements in triplet yield and activation, it is important to also consider how the chalcogenide might also impact deactivation during the polymerization. In this case, PC **2** appears to have the best balance of properties, since the presence of S likely increases triplet yield without significantly impacting PC^⋅+^ stability or PC^⋅+^ formation.

**Figure 7:**
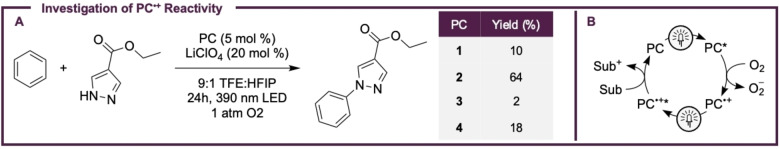
(a) Scheme for the photooxidation reported by Wickens and coworkers^[24]^ that was used to test PC^⋅+^ reactivity in this work (see the *Experimental* section for more details.), including ^1^H NMR yields obtained by PCs **1**–**4**. (b) Proposed mechanism for the generation of PC^⋅+^ from the PC and O_2_.

Another reaction in which these PCs may be useful is in the photooxidation of pyrazoles reported by Wickens and coworkers in 2021 (Figure 7a). In this reaction, it has been proposed that PC^⋅+^ can be generated by the reaction of a PC with an oxidant, such as O_2_. The PC^⋅+^ could then undergo photoexcitation to generate a PC^⋅+^ excited state, which may act as a super‐oxidant capable of oxidizing challenging substrates (Figure 7b). In the work by Wickens and coworkers, this reactivity was harnessed to perform a C−N coupling with pyrazoles and benzene, and PC **2** was shown to operate most effectively in this reaction.^[24]^ However, excited state radicals such as PC^⋅+^* typically have extremely short lifetimes in the range of picoseconds,^[32]^ which can inhibit their ability to engage in bimolecular reactions. As such, we wondered if changing the chalcogenide to Se or Te could improve catalytic activity, possibly by extending the lifetime of PC^⋅+^* through the formation of a longer‐lived quartet excited state.

To test this hypothesis, PCs **1**–**4** were employed in this reaction under the optimized conditions reported by Wickens and coworkers.^[24]^ The yield of the desired product was determined by ^1^H NMR relative to a dibromomethane internal standard, revealing PC **2** gives the highest yield (64 %), followed by PCs **4** (18 %), **1** (10 %), and **3** (2 %). Photooxidation control reactions performed without light resulted in no desired product (0 % yield), supporting the proposed photocatalytic mechanism (Figures S72–S75). Based on these results, PC **2** remains the best catalyst for this reaction. It is possible that the poor stability of PC^⋅+^ for **3** and **4** may cause catalyst degradation in this case, resulting in the lower yields observed for these PCs. In addition, it is important to note that these reactions were performed using 390 nm light, which is poorly absorbed by all four PCs, but especially so for **3** and **4**. In turn, this poor absorption of the light source may limit the formation of PC^⋅+^, thus inhibiting this reaction. It is possible that increasing the energy of the light source could improve yield with PCs **3** and **4**, although similar improvements would also be expected with PCs **1** and **2**, and higher energy light could also lead to increased side reactions through the activation of other molecules in solution.[^21^, ^33^]

## Conclusions

Enabled by recent synthetic advancements, this work sought to evaluate the impact of the chalcogenide atom in phenochalcogenazine PCs. As anticipated, increasing the size of the chalcogenide causes structural distortions within PCs **1**–**4**, which we expected would impact PC properties in a predictable fashion. While many photophysical and electrochemical properties do not show clear trends with the chalcogenide identity, two clear trends have emerged which include increasing the energy of light absorbed by the PC (i. e. blue‐shifting its absorption) and increasing the reorganization energy penalty experienced during ET reactions.

In O‐ATRP, a balance of the effects of the chalcogenide must be achieved for optimal catalysis. While it is anticipated that larger chalcogenides will increase the yield of the triplet excited state, which is believed to be beneficial for catalysis, the increase in reorganization energy that accompanies these larger chalcogenides may be detrimental to the polymerization process. In addition, PCs containing Se (PC **3**) and Te (PC **4**) exhibit poor PC^⋅+^ stability, which may cause catalyst degradation and limit polymerization control in O‐ATRP. It may be possible to overcome some of these issues through further catalyst functionalization (i. e. altering the *N*‐aryl group or adding core substituents). However, it should be noted that the limitations of PC **1** – namely the lower triplet yield expected for this catalyst – have previously been addressed through catalyst functionalization.[^7^, ^13^, ^15^, ^34^] Moreover, these strategies for increasing the triplet yield of phenoxazine PCs do not negatively impact the reorganization energies or radical cation stability of these PCs, yielding perhaps the best balance of properties currently available for phenochalcogenazine PCs in O‐ATRP.

With regard to the Wickens photooxidation reaction,^[24]^ PC **2** remains the best performing catalyst, although PC **4** also shows some promise in this reaction. It is hypothesized that the poor radical cation stability of PCs **3** and **4** is a major limitation of these catalysts. As such, it may be beneficial to explore synthetic strategies that could improve radical cation stability in this catalyst family, such as substitution of the catalyst core.[^28^, ^35^] In addition to benefiting catalyst stability, catalyst substitution could also red‐shift the absorption of these PCs, allowing lower energy light to be employed effectively. Together, these improvements could yield improved catalyst performance in this photooxidation reaction, making this a promising area of future research.

## Experimental Section

*Synthesis of **1***: 10‐Phenyl phenoxazine was synthesized according to a modified literature procedure.^[13]^ A storage tube was equipped with phenoxazine (4.25 g, 23.2 mmol, 1 eq), sodium *t*‐butoxide (6.70 g, 69.6 mmol, 3 eq) and degassed bromobenzene (3.7 mL, 35 mmol, 1.5 eq) under a nitrogen atmosphere. In an nitrogen filled glovebox, palladium(0) bis(dibenzylideneacetone) (154 mg, 0.232 mmol, 0.01 eq), tri‐*t*‐butyl phosphine (156 g, 0.696 mmol, 0.03 eq), and toluene (200 mL) were added to the storage tube. The reaction was heated at 110 °C for 24 h, cooled to room temperature, and dried by rotary evaporation. The crude mixture was purified washing in DCM (500 mL) with water (2×500 mL) and brine (1×500 mL). The organic layer was dried with magnesium sulfate and concentrated by rotary evaporation. The crude solid was then purified by sublimation (200 °C, 100 mtorr). The resulting off‐white solid was collected, rinsed with hexanes, and dried under high vacuum. Yield=5.2 g (87 %). ^1^H NMR (400 MHz, C_6_D_6_): *δ*=7.12–7.06 (m, 2H), 7.03–6.98 (m, 1H), 6.93–6.88 (m, 2H), 6.77 (dd, 2H), 6.49 (td, 2H), 6.42 (td, 2H), 5.91 ppm (dd, 2H); UV/Vis (DMAc): *λ*_max_ (*ϵ*)=322 (9000 M^−1^ cm^−1^); fluorescence (DMAc): *λ*_ex_=322 nm; *λ*_em_=391.

*Synthesis of **2***: 10‐Phenyl phenothiazine was synthesized according to a modified literature procedure.^[13]^ A storage tube was equipped with phenothiazine (4.35 g, 21.8 mmol, 1 eq), sodium *t*‐butoxide (6.34 g, 65.4 mmol, 3 eq) and degassed bromobenzene (3.5 mL, 33 mmol, 1.5 eq) under a nitrogen atmosphere. In an nitrogen filled glovebox, palladium(0) bis(dibenzylideneacetone) (143 mg, 0.218 mmol, 0.01 eq), tri‐*t*‐butyl phosphine (147 g, 0.654 mmol, 0.03 eq), and toluene (200 mL) were added to the storage tube. The reaction was heated at 110 °C for 24 h, cooled to room temperature, and dried by rotary evaporation. The crude mixture was purified washing in DCM (400 mL) with water (2×400 mL) and brine (1×400 mL). The organic layer was dried with magnesium sulfate and concentrated by rotary evaporation. The crude solid was then purified by sublimation (200 °C, 100 mtorr). The resulting off‐white solid was collected, rinsed with hexanes, and dried under high vacuum. Yield=4.3 g (72 %). ^1^H NMR (400 MHz, C_6_D_6_): *δ*=7.11–7.05 (m, 2H), 7.04–6.92 (m, 5H), 6.64–6.54 (m, 4H), 6.21–6.15 ppm (m, 2H); UV/Vis (DMAc): *λ*_max_ (*ϵ*)=317 (3600 M^−1^ cm^−1^); fluorescence (DMAc): *λ*_ex_=317 nm; *λ*_em_=444.

*Synthesis of **3***: A stirred mixture of 10H‐phenoselenazine^[36]^ (246 mg, 1 mmol), iodobenzene (134 μL, 1.2 mmol, 1.2 equiv.), NaOtBu (134 mg, 1.4 mmol, 1.4 equiv.), Pd_2_(dba)_3_ (46 mg, 0.05 mmol, 5 mol%) and DPPF (55 mg,0.1 mmol, 10 mol%) in toluene were heated to 120 °C in a closed 20 mL vial for 24 h. The solvent was removed in vacuo. It was taken up in sat. NH_4_Cl solution, extracted with ethyl acetate and dried over Na_2_SO_4_. The solvent was removed on silica. The crude product was purified by flash column chromatography in hexane/dichloromethane (6 : 4) yielding the title compound as yellow solid. Yield=198 mg (61 %).^1^H NMR (400 MHz, CDCl_3_): *δ*=7.78 (t, 2H), 7.65–7.53 (m, 5H), 7.31 (td, 2H), 7.21 (td, 2H), 6.96 ppm (dd, 2H); ^13^C NMR (101 MHz, CDCl_3_): *δ*=144.13 (s, C_quat_), 143.07 (s, C_quat_), 130.26 (s, CH), 130.01 (s, CH), 127.42 (s, CH), 127.40 (s, CH), 126.34 (s, CH), 123.83 (s, CH), 120.25 (s, CH), 119.98 ppm (s, C_quat_); ^77^Se NMR (115 MHz, CDCl_3_): *δ*=275.45 ppm (t); IR (neat): $\tilde{\nu }$ =3055, 2325, 2109, 1899, 1782, 1580, 1488, 1453, 1296, 1242, 1159, 1117, 1062, 1031, 969, 935, 896, 843, 740, 702 cm^−1^; UV/Vis (DMAc): *λ*_max_ (*ϵ*)=310 (4000 M^−1^ cm^−1^); fluorescence (DMAc): *λ*_ex_=355 nm; *λ*_em_=419; HRMS (ESI): *m*/*z* calcd for C_18_H_14_NSe ([M+H]^+^): 324.02860; found: 324.02874.

*Synthesis of **4***: 10‐Phenyl phenotellurazine was prepared according to a literature procedure.^[37]^

*General Procedure for O‐ATRP*: Photocatalyst (0.00935 mmol, 1 eq) was weighed into a 20 mL scintillation vial and brought into a nitrogen filled glovebox. DMAc (1 mL), methyl methacrylate (1 mL, 9.35 mmol, 1000 eq), and diethyl‐2‐bromo‐2‐methyl malonate (17.9 μL, 0.0935, 10 eq) were added to the vial with minimal light exposure. The vial was then placed in a 365 nm LED beaker to drive the reaction (see *Materials and Methods* in the *Supporting Information*).

*General Procedure for Photooxidation Reactions*: Photooxidation reactions were performed according to the literature procedure published by Wickens and coworkers.^[24]^

## Conflict of interest

The authors declare no conflict of interest.

1

## Supporting information

As a service to our authors and readers, this journal provides supporting information supplied by the authors. Such materials are peer reviewed and may be re‐organized for online delivery, but are not copy‐edited or typeset. Technical support issues arising from supporting information (other than missing files) should be addressed to the authors.

Supporting Information

## Data Availability

The data that support the findings of this study are available in the supplementary material of this article.
