# Supplementary material for: Effects of the Chalcogenide Identity in N‐Aryl Phenochalcogenazine Photoredox Catalysts
Source: ChemCatChem. 2022 Jul 8;14(17):e202200485. doi: 10.1002/cctc.202200485 (PMC9541587; doi:10.1002/cctc.202200485)
Supplement: Supplementary file 1 — Supporting Information [file CCTC-14-0-s001.pdf]

ChemCatChem

Supporting Information

Effects of the Chalcogenide Identity in *N*-Aryl Phenochalcogenazine Photoredox Catalysts

Daniel A. Corbin, Christopher Cremer, Katherine O. Puffer, Brian S. Newell, Frederic W. Patureau,* and Garret M. Miyake*

Table of Contents

1. MATERIALS AND METHODS 4

PURCHASED CHEMICALS 4
CHEMICAL PREPARATION AND STORAGE 4
EXPERIMENTAL EQUIPMENT 5
INSTRUMENTATION 5

2. CRYSTALLOGRAPHIC INFORMATION FOR PCS 1 – 4 7

INFORMATION FOR PC 1 7
INFORMATION FOR PC 3 11
INFORMATION FOR PC 4 13

3. UV-VISIBLE ABSORPTION SPECTROSCOPY AND RELATED DATA 15

DETERMINATION OF MOLAR ABSORPTIVITY 15
OVERLAID SPECTRA 19
INSIGHTS FROM DENSITY FUNCTIONAL THEORY 20

4. STEADY STATE EMISSION SPECTROSCOPY 24

FLUORESCENCE SPECTROSCOPY 24
PHOSPHORESCENCE SPECTROSCOPY 32
STERN-VOLMER QUENCHING EXPERIMENTS 36

5. FLUORESCENCE QUANTUM YIELD MEASUREMENTS 40

6. TIME CORRELATED SINGLE PHOTON COUNTING 44

7. ELECTROCHEMICAL CHARACTERIZATION 48

CYCLIC VOLTAMMETRY IN *N,N*-DIMETHYLACETAMIDE 48
CYCLIC VOLTAMMETRY IN DICHLOROMETHANE 53
ESTIMATION OF $E_{1/2}$ IN *N,N*-DIMETHYLACETAMIDE FROM DATA IN DICHLOROMETHANE 58

8. SUPPLEMENTAL POLYMERIZATION DATA 59

PROCEDURE FOR THE ANALYSIS OF KINETICS AND MOLECULAR WEIGHT GROWTH 59
SUPPLEMENTAL DATA 59

9. SUPPLEMENTAL PHOTOOXIDATION DATA 64

PROCEDURE FOR REACTION ANALYSIS 64
REACTION NMR SPECTRA 64
CONTROL NMR SPECTRA 70

10. COMPUTATIONAL DETAILS AND DATA 74

COMPUTATIONAL DETAILS 74
RESULTS 75
MOLECULAR COORDINATES 85

11. NMR SPECTRA FOR PC 3 99

12. REFERENCES 102

1. Materials and Methods

Purchased Chemicals

*For the Synthesis of N-Phenyl Phenoxazine (**1**):* Bis(dibenzylideneacetone)palladium(0), sodium *t*-butoxide, and tri-*t*-butylphosphine were purchased from Sigma Aldrich. Phenoxazine was purchased from Accela. Bromobenzene was obtained from TCI America. Toluene was obtained from an mBraun MB-SPS-800 solvent purification system.

*For the Synthesis of N-Phenyl Phenothiazine (**2**):* Bis(dibenzylideneacetone)palladium(0), sodium *t*-butoxide, tri-*t*-butylphosphine, and phenothiazine were purchased from Sigma Aldrich. Bromobenzene was obtained from TCI America. Toluene was obtained from an mBraun MB-SPS-800 solvent purification system.

For the Synthesis of Phenoselenazine: Selenium powder was purchased from Alfa Aesar.

*For the Synthesis of N-Phenyl Phenoselenazine (**3**):* Bis(dibenzylideneacetone) palladium(0) was purchased from Sigma Aldrich. Sodium t-butoxide was purchased from TCI Deutschland GmbH. Iodobenzene was obtained from BLDpharm. 1,1'-Bis(diphenylphosphino)ferrocene was purchased from fluorochem.

For the Synthesis of Phenotellurazine: Tellurium powder was purchased from Acros Organics.

*For the Synthesis of N-Phenyl Phenotellurazine (**4**):* Bis(dibenzylideneacetone) palladium(0) was purchased from Sigma Aldrich. Sodium t-butoxide was purchased from TCI Deutschland GmbH. Iodobenzene was obtained from BLDpharm. 1,1'-Bis(diphenylphosphino)ferrocene was purchased from fluorochem.

For Electrochemical Experiments: *N,N*-Dimethylacetamide (DMAc), silver nitrate, acetonitrile, and ferrocene were purchased from Sigma Aldrich. Tetra-*n*-butylammonium hexafluorophosphate (Bu_4NPF_6) was purchased from TCI America. Dichloromethane (DCM) was purchased from Fisher Scientific.

For Organocatalyzed Atom Transfer Radical Polymerization (O-ATRP): DMAc, methyl methacrylate (MMA), and diethyl-2-bromo-2-methylmalonate (DBMM) were purchased from Sigma Aldrich.

For Photooxidation Reactions: Ethyl 1H-pyrazole-4-carboxylate hydrochloride, lithium perchlorate, trifluoroethanol, and 1,1,1,3,3,3-hexafluoroisopropanol were purchased from Oakwood Chemical. Benzene was purchased from Sigma Aldrich. Sodium bicarbonate was purchased from Arm and Hammer. Oxygen was purchased from Airgas.

Chemical Preparation and Storage

Unless otherwise stated, chemicals and reagents were used as received from the manufacturer. Bis(dibenzylideneacetone)palladium(0) and tri-*t*-butyl phosphine were stored in a nitrogen filled glovebox until their use. Toluene for Buchwald couplings was purified using an mBraun MB-SPS-800 solvent purification system and stored under nitrogen in a glovebox until it was used. All photocatalysts (PCs) used in this work were stored under air.

For polymerizations, MMA and DBMM were dried overnight using CaH_2, vacuum distilled, and degassed by three freeze-pump-thaw cycles. Both chemicals were then stored in a nitrogen

filled glovebox at -40 ºC until their use. DMAc for polymerizations was also stored in a nitrogen filled glovebox prior to use.

For photooxidations, the pyrazole ester used in these reactions was prepared from the hydrochloride salt obtained from the chemical manufacturer according to a published literature procedure.[1] Yield and ^{1}H NMR characterization matched those reported in the literature.

Experimental Equipment

Light beakers were constructed in the following manner. LED strips (365 nm emission) were purchased from LEDLightingHut.com (item no. LLH-UVFS-365NM). Reactors were constructed by wrapping a 400 mL beaker (10.0 cm tall, 8.5 cm diameter) with aluminum foil and wrapping LED strips (9 LED segments, 16” total) around the inside of the reactor (Figure S1).

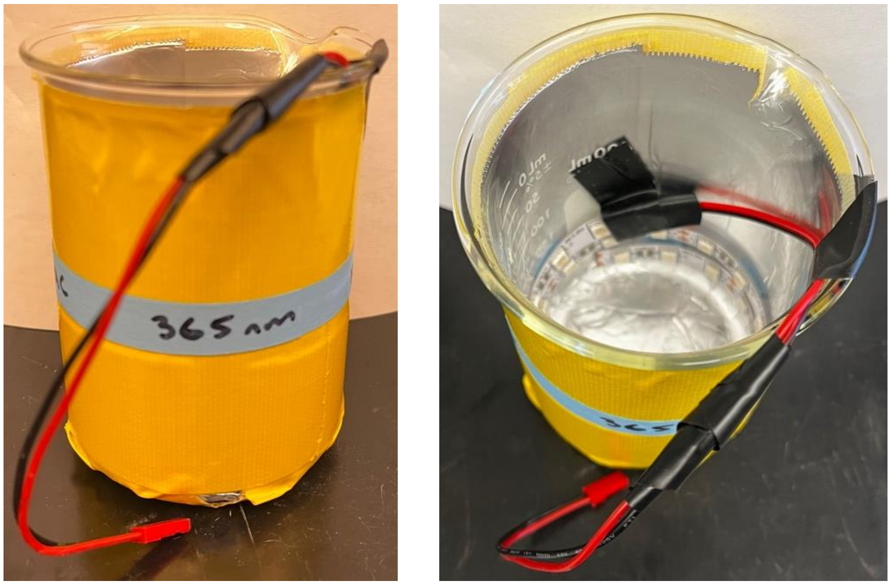

Figure S1. Photographs of LED beaker photoreactors from the side (left) and top (right).

For photooxidation reactions, a HepatoChem PhotoRedOx Box TC photoreactor was used and cooled by blowing compressed air through the reactor body. Irradiation for these reactions was achieved using a 390 nm Kessil LED lamp (PR160L-390).

Instrumentation

Nuclear magnetic resonance (NMR) spectroscopy was performed using either a Bruker US 400 MHZ spectrometer, a Bruker Ascend 400 MHZ spectrometer, an Agilent VNMRS 400 MHz spectrometer or a Bruker Av 600 MHz spectrometer. All ^{1}H NMR spectra are reported in δ units, parts per million (ppm), and are referenced to residual chloroform (7.26 ppm) or benzene (7.15). High resolution mass spectrometry was performed using a Bruker Maxis QTOF UPLC-MA or a ThermoFisher Scientific LTQ Orbitrap XL spectrometer with an ESI source in positive ion mode. IR spectra were measured on a PerkinElmer 100 FT-IR spectrometer with an UATR Diamond KRS-5 unit.

Structures were determined for the compounds listed in Figure 2b. Single crystals were coated with Paratone-N oil and mounted under a cold stream of dinitrogen gas. Single crystal X-ray diffraction data were acquired on a Bruker D8 QUEST diffractometer equipped with a Photon50 CMOS detector and curved graphite monochromator using Mo Kαradiation (λ =

0.71073 Å). Initial lattice parameters were obtained from a least-squares analysis of more than 100 reflections; these parameters were later refined against all data. None of the crystals showed significant decay during data collection. Data were integrated and corrected for Lorentz and polarization effects using Bruker APEX4 software, and semiempirical absorption corrections were applied using SCALE.[2] Space group assignments were based on systematic absences, E statistics, and successful refinement of the structures. Structures were solved using Direct Methods and were refined with the aid of successive Fourier difference maps against all data using the SHELXTL 6.14 software package.[3] Thermal parameters for all non-hydrogen atoms were refined anisotropically. All hydrogen atoms were assigned to ideal positions and refined using a riding model with an isotropic thermal parameter 1.2 times that of the attached carbon atom (1.5 times for methyl hydrogens). For *R*1 and *wR*2, the following definitions apply: *R*1 = S ||*F*o| – |*F*c|| / S |*F*o|; *wR*2 = {S[*w*(*F*o2 – *F*c2)2] / S[*w*(*F*o2)2]}1/2. Selected bond distances and angles for crystals of compounds **1 – 4** are collected in Figures S3, S5, S7, and S9. All other metric parameters can be found in the .CIF files included with the Supporting Information. Crystal structure figures were produced using Mercury.[4]

Analysis of polymer molecular weights were performed via gel permeation chromatography (GPC) coupled with multi-angle light scattering (MALS), using an Agilent HPLC fitted with one guard column, three PLgel 5 μm MIXED-C gel permeation columns, a Wyatt Technology TrEX differential refractometer, and a Wyatt Technology miniDAWN TREOS light scattering detector, using THF as the eluent at a flow rate of 1.0 mL/min. A dn/dc value of 0.084 was used for PMMA molecular weight analysis.

Electrochemical measurements were performed using either a Gamry Interface 1010B or 1010E potentiostat. UV-Visible spectroscopy was performed using an Agilent Cary 5000 UV-Vis-NIR spectrometer. Steady state fluorescence spectroscopy was performed using an FS5 Spectrofluorometer from Edinburgh Instruments. Fluorescence quantum yield measurements were performed using an FS5 Spectrofluorometer from Edinburgh Instruments equipped with an SC-30 integrating sphere. Time correlated single photon counting (TCSPC) was performed using an FS5 Spectrofluorometer from Edinburgh Instruments with a TCSPC upgrade and either a 295 nm or 365 nm EPLED from Edinburgh Instruments.

Phosphorescence measurements were performed using an Edinburgh Instruments LP980KS spectrometer with a Minilite Nd-YAG Q-switched laser (Continuum Lasers) configured to deliver a 355 nm excitation pulse. Spectral emission data was obtained with the indicated time delays using an iStar ICCD camera (Andor) as the detector. Time zero was set on the instrument using the emission of $[Ru(bpy)_3]Cl_2$ to locate the pump pulse with a resolution of 1 ns.

2. Crystallographic Information for PCs 1 – 4

Information for PC 1

Crystals for PC **1** were grown by dissolving **1** in hexanes and allowing the solution to evaporate to dryness. The resulting crystals were clear, colorless blocks. See the attached .CIF file for the full crystal structure and experiment details.

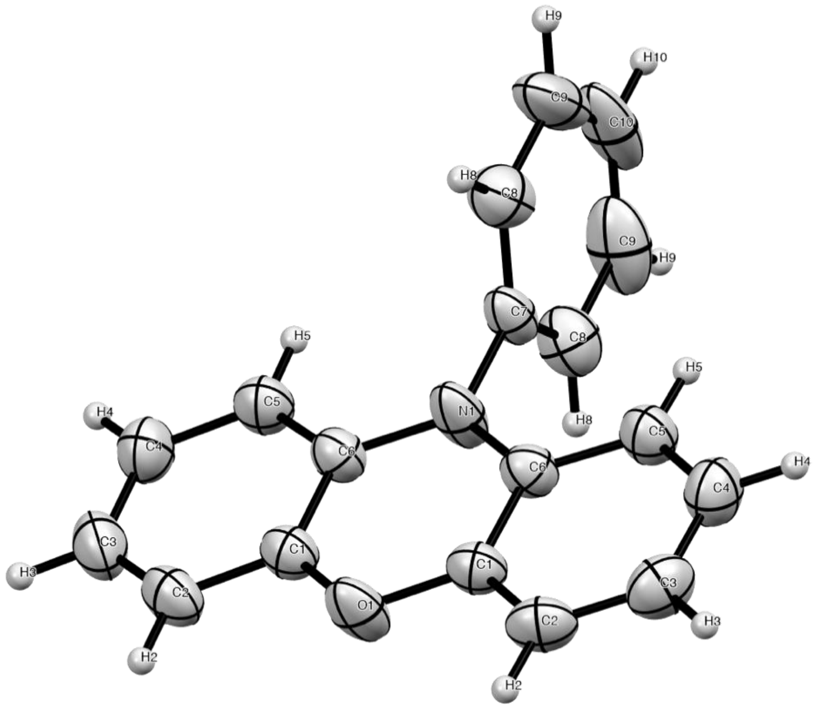

Figure S2. Crystal structure of **1** shown as an ORTEP plot.

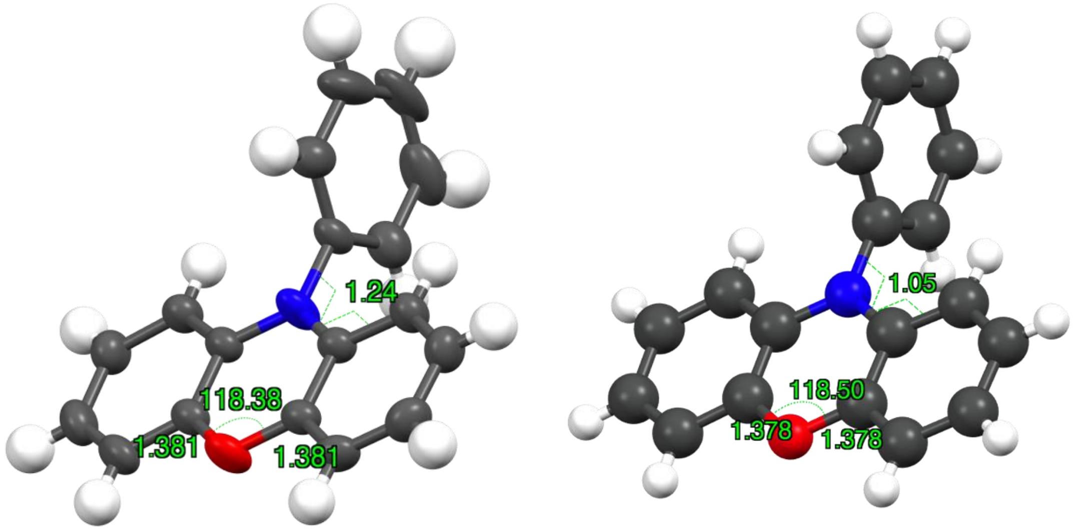

Figure S3. (Left) Crystal structure of **1** collected in this work with C-O bond lengths, C-O-C bond angles, and core/*N*-aryl dihedral angles labeled. (Right) Crystal structure of **1**[5] previously reported for comparison.

Table S1. Crystallographic information for the structural refinement of **1**.

Empirical formula	$C_{18}H_{13}NO$
Formula weight	259.29
Temperature/K	250.(2)
Crystal system	monoclinic
Space group	C2/c
a/Å	14.8144(8)
b/Å	10.5396(6)
c/Å	10.2429(6)
α/°	90
β/°	125.405(2)
γ/°	90
Volume/Å^3	1303.56(13)
Z	4
ρ_{calc}g/cm^3	1.321
μ/mm^{-1}	0.082
F(000)	544.0
Crystal color	Clear colorless
Crystal size/mm^3	0.103 × 0.068 × 0.039
Radiation	Mo Kα (λ = 0.71073)
2Θ range for data collection/°	5.12 to 51.34
Index ranges	-18 ≤ h ≤ 18, -12 ≤ k ≤ 12, -12 ≤ l ≤ 12
Reflections collected	22842
Independent reflections	1242 [R_{int} = 0.0805, R_{sigma} = 0.0252]
Data/restraints/parameters	1242/0/93
Goodness-of-fit on F^2	1.032
Final R indexes [I>=2σ (I)]	R_1 = 0.0442, wR_2 = 0.1098
Final R indexes [all data]	R_1 = 0.0873, wR_2 = 0.1339
Largest diff. peak/hole / e Å^{-3}	0.13/-0.17

Information for PC 2

Crystals for PC **2** were grown by dissolving **2** in hexanes and allowing the solution to evaporate to dryness. The resulting crystals were clear, colorless rods. See the attached .CIF file for the full crystal structure and experiment details.

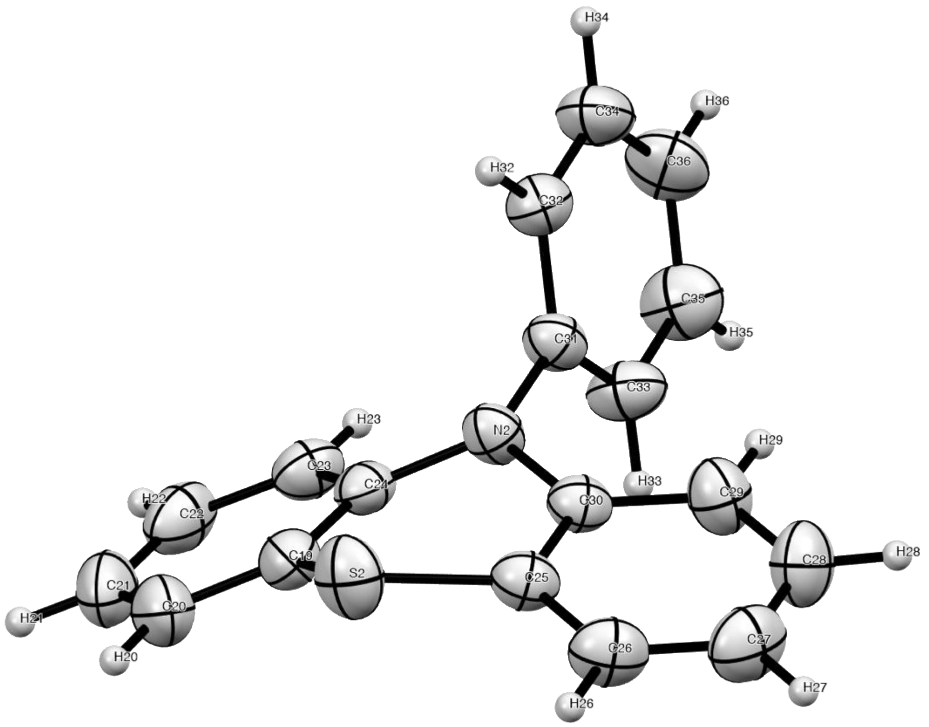

Figure S4. Crystal structure of **2** shown as an ORTEP plot.

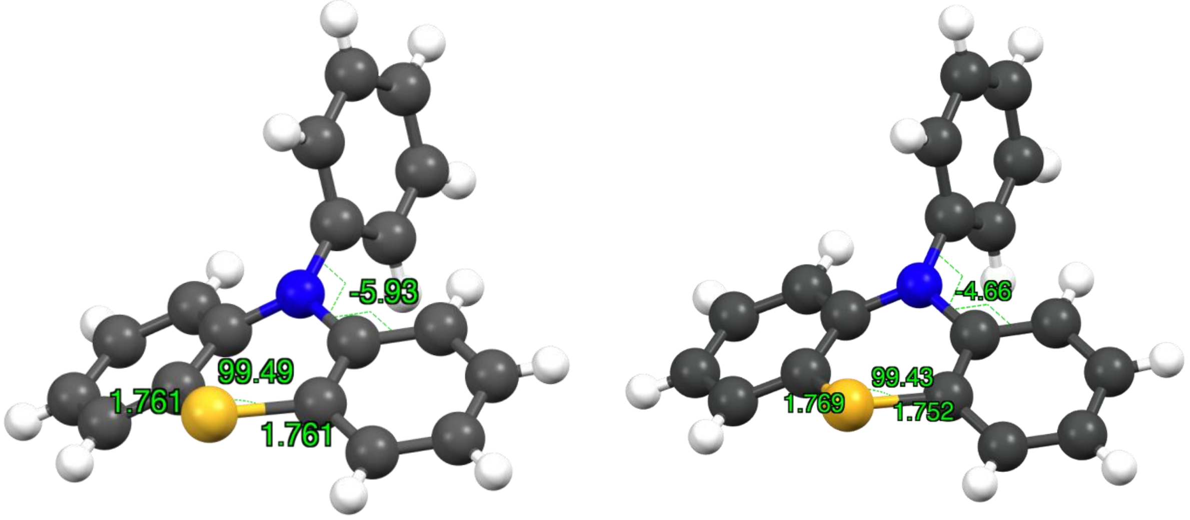

Figure S5. (Left) Crystal structure of **2** collected in this work with C-S bond lengths, C-S-C bond angles, and core/*N*-aryl dihedral angles labeled. (Right) Crystal structure previously reported for **2**[6] for comparison.

Table S2. Crystallographic information for the structural refinement of **2**.

Empirical formula	$C_{18}H_{13}NS$
Formula weight	275.35
Temperature/K	250.(2)

Crystal system	triclinic
Space group	P-1
a/Å	11.7811(3)
b/Å	14.7447(4)
c/Å	17.5729(5)
α/°	97.3740(10)
β/°	90.0450(10)
γ/°	110.6140(10)
Volume/Å^3	2830.01(13)
Z	8
ρ_{calc}g/cm^3	1.293
μ/mm^{-1}	0.217
F(000)	1152.0
Crystal color	Clear colorless
Crystal size/mm^3	0.12 × 0.04 × 0.032
Radiation	Mo Kα (λ = 0.71073)
2Θ range for data collection/°	3.52 to 50.06
Index ranges	-14 ≤ h ≤ 14, -17 ≤ k ≤ 17, -20 ≤ l ≤ 20
Reflections collected	113138
Independent reflections	9958 [R_{int} = 0.0957, R_{sigma} = 0.0406]
Data/restraints/parameters	9958/0/721
Goodness-of-fit on F^2	1.017
Final R indexes [I>=2σ (I)]	R_1 = 0.0439, wR_2 = 0.0950
Final R indexes [all data]	R_1 = 0.0809, wR_2 = 0.1110
Largest diff. peak/hole / e Å^{-3}	0.42/-0.43

Information for PC 3

Crystals for PC **3** were grown by dissolving **3** in a minimal quantity of hot hexanes and then allowing the solution to cool slowly to -25 ºC. The resulting crystals were clear, colorless blocks. See the attached .CIF file for the full crystal structure and experiment details.

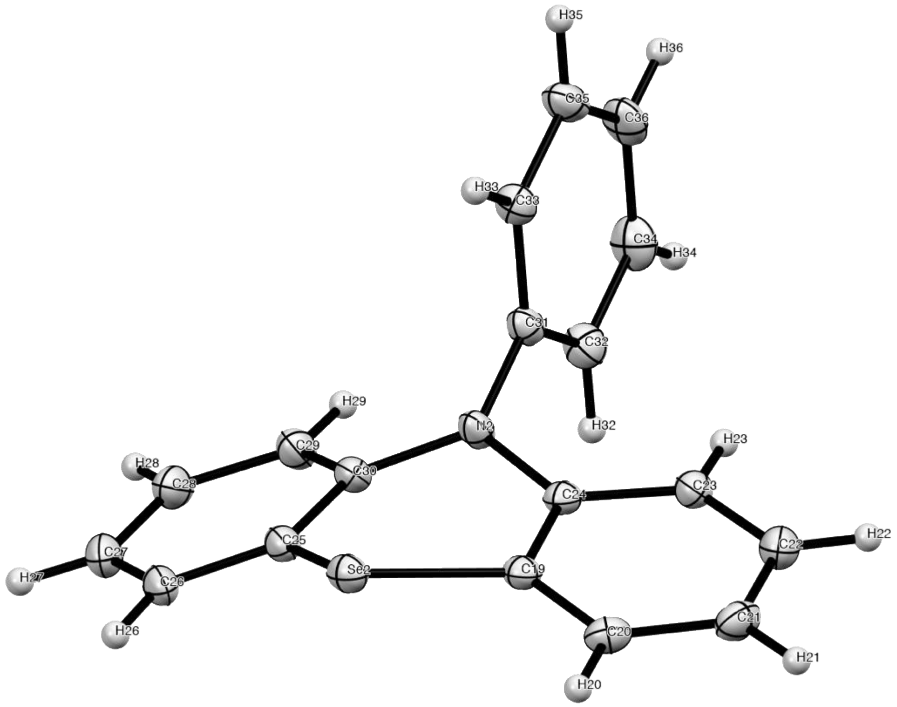

Figure S6. Crystal structure of **3** shown as an ORTEP plot.

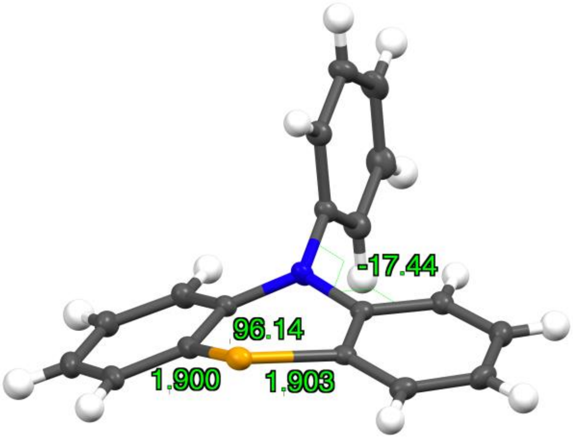

Figure S7. Crystal structure of **3** with C-Se bond lengths, C-Se-C bond angles, and core/*N*-aryl dihedral angles labeled.

Table S3. Crystallographic information for the structural refinement of **3**.

Empirical formula	$C_{18}H_{13}NSe$
Formula weight	322.25
Temperature/K	100.(2)
Crystal system	triclinic
Space group	P-1
a/Å	9.1008(3)
b/Å	9.1593(3)
c/Å	36.5463(11)
α/°	96.5740(10)
β/°	91.4440(10)
γ/°	112.3120(10)
Volume/Å^3	2791.95(16)
Z	8
ρ_{calc}g/cm^3	1.533
μ/mm^{-1}	2.678
F(000)	1296.0
Crystal color	Clear colorless
Crystal size/mm^3	0.102 × 0.071 × 0.058
Radiation	Mo Kα (λ = 0.71073)
2Θ range for data collection/°	3.38 to 66.46
Index ranges	-14 ≤ h ≤ 14, -14 ≤ k ≤ 14, -56 ≤ l ≤ 56
Reflections collected	175777
Independent reflections	21358 [R_{int} = 0.0707, R_{sigma} = 0.0388]
Data/restraints/parameters	21358/0/721
Goodness-of-fit on F^2	1.028
Final R indexes [I>=2σ (I)]	R_1 = 0.0380, wR_2 = 0.0828
Final R indexes [all data]	R_1 = 0.0627, wR_2 = 0.0932
Largest diff. peak/hole / e Å^{-3}	1.27/-0.90

Information for PC 4

Crystals of PC **4** were grown by dissolving **4** in a mixture of 10:1 hexanes and DCM and allowing the solution to evaporate to dryness. The resulting crystals were clear, colorless rods. See the attached .CIF file for the full crystal structure and experiment details.

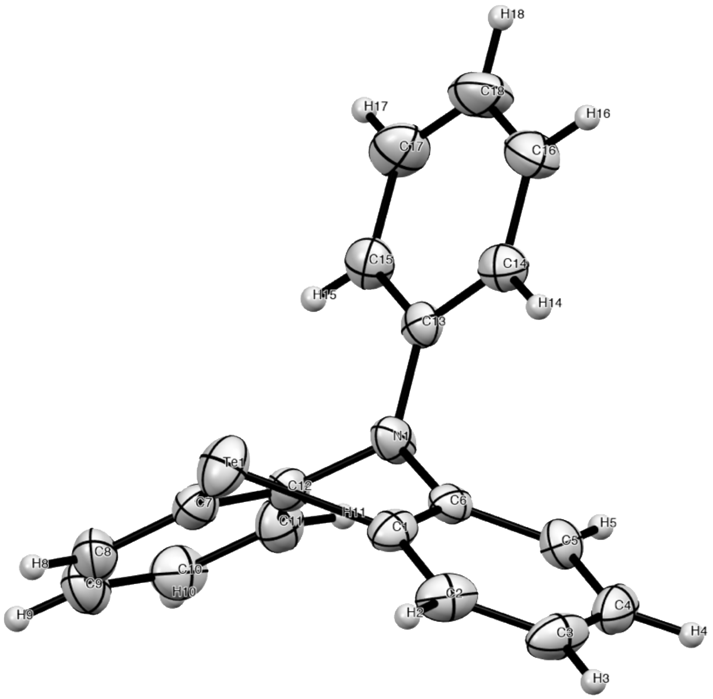

Figure S8. Crystal structure of **4** shown as an ORTEP plot.

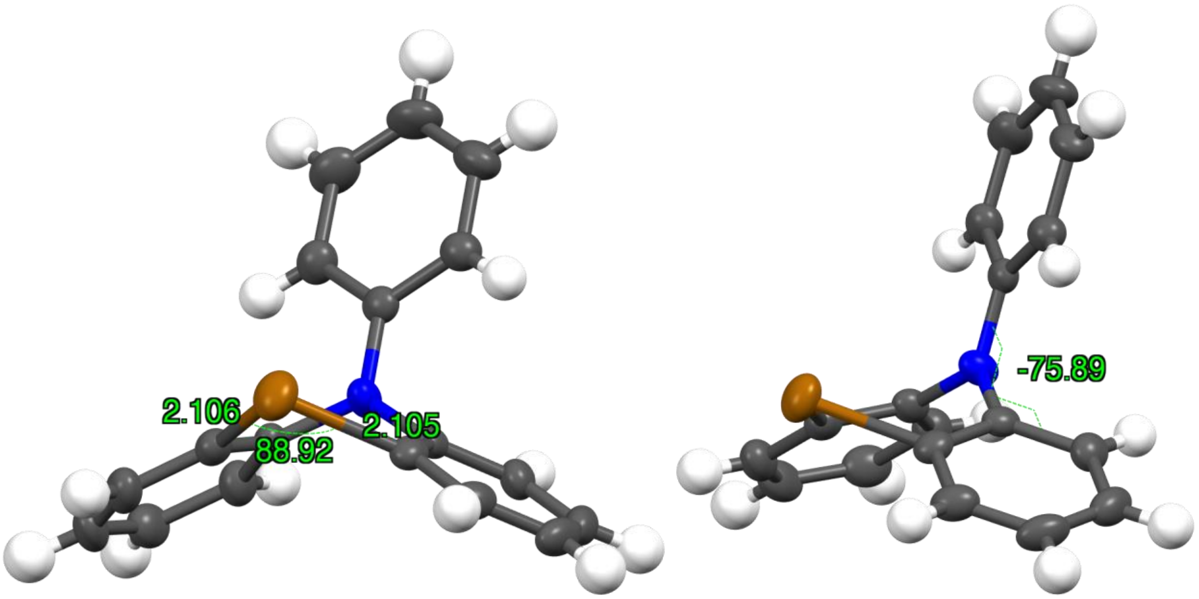

Figure S9. Crystal structure of **4** with C-Te bond lengths, C-Te-C bond angles, and core/*N*-aryl dihedral angles labeled.

Table S4. Crystallographic information for the structural refinement of **4**.

Empirical formula	$C_{18}H_{13}NTe$
Formula weight	370.89
Temperature/K	250.(2)
Crystal system	orthorhombic
Space group	Pbca
a/Å	12.5866(4)
b/Å	7.6043(2)
c/Å	29.9178(8)
α/°	90
β/°	90
γ/°	90
Volume/Å^3	2863.50(14)
Z	8
ρ_{calc}g/cm^3	1.721
μ/mm^{-1}	2.066
F(000)	1440.0
Crystal color	Clear colorless
Crystal size/mm^3	0.101 × 0.046 × 0.025
Radiation	Mo Kα (λ = 0.71073)
2Θ range for data collection/°	4.24 to 50.06
Index ranges	-14 ≤ h ≤ 14, -9 ≤ k ≤ 9, -35 ≤ l ≤ 35
Reflections collected	104059
Independent reflections	2524 [R_{int} = 0.0518, R_{sigma} = 0.0107]
Data/restraints/parameters	2524/0/181
Goodness-of-fit on F^2	1.232
Final R indexes [I>=2σ (I)]	R_1 = 0.0284, wR_2 = 0.0613
Final R indexes [all data]	R_1 = 0.0325, wR_2 = 0.0630
Largest diff. peak/hole / e Å^{-3}	0.47/-0.55

3. UV-Visible Absorption Spectroscopy and Related Data

Determination of Molar Absorptivity

The molar absorptivity of each PC was determined using the following procedure: a stock solution of the PC was prepared with a concentration of 1.3 mM in DMAc. This solution was then diluted to produce six solutions of different concentrations with a maximum absorbance roughly between 0 and 1. For PCs **1** and **2**, a clear $\lambda_{max,abs}$ around 320 nm was observed, and molar absorptivity (ε_{max}) was determined at this wavelength. For PC **3**, a shoulder around 310 nm was observed resembling an absorption peak that is overlaid with another absorption feature. As such, the $\lambda_{max,abs}$ for PC **3** was estimated to be 310 nm and ε_{max} was measured at this wavelength. For PC **4**, a similar shoulder was observed, in addition to a blue-shifted peak. In this case, ε_{max} was measured for both features (the $\lambda_{max,abs}$ at 274 nm and the shoulder's $\lambda_{max,abs}$ around 290 nm).

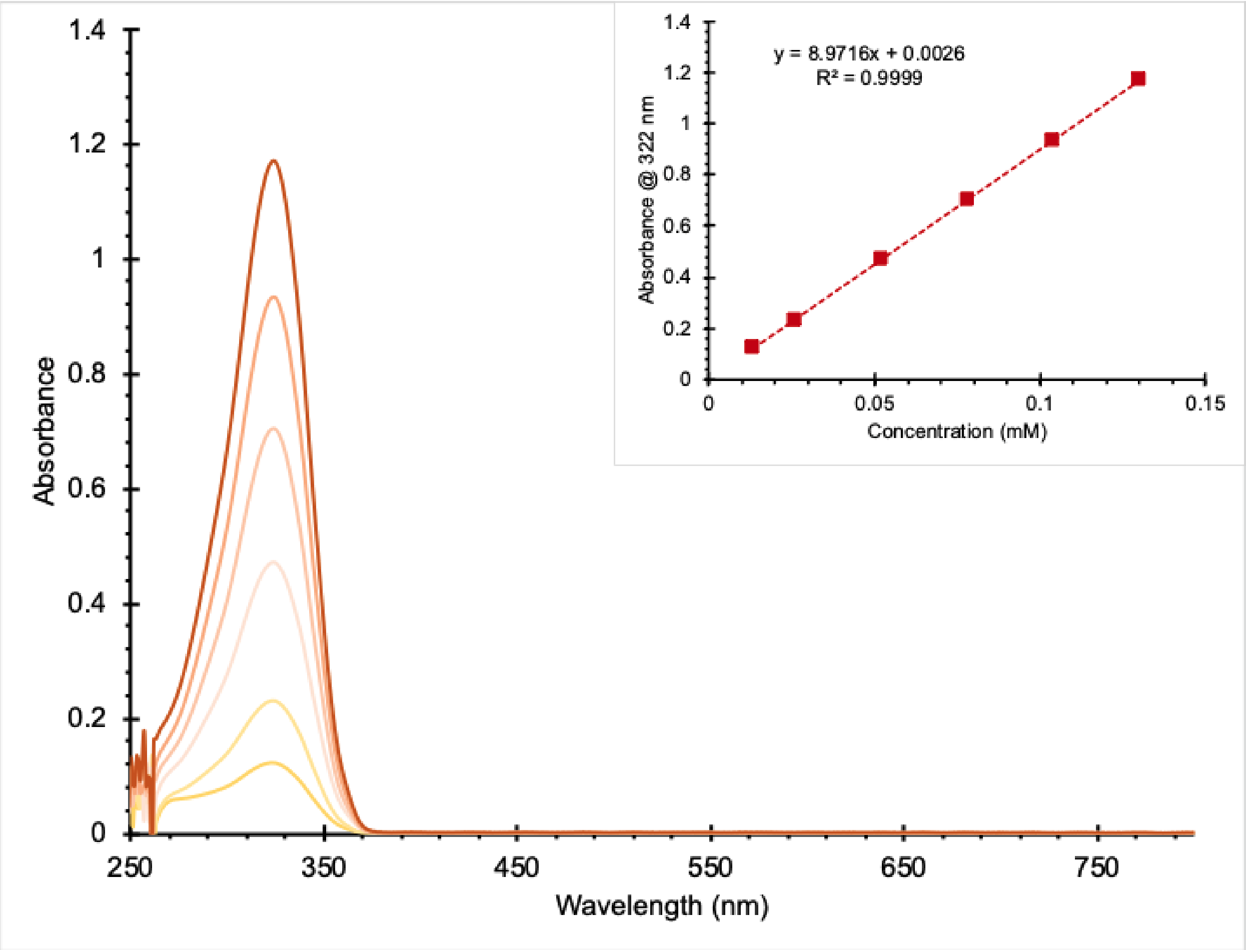

Figure S10. UV-Visible absorption spectra used to determine the molar absorptivity of **1** in DMAc.

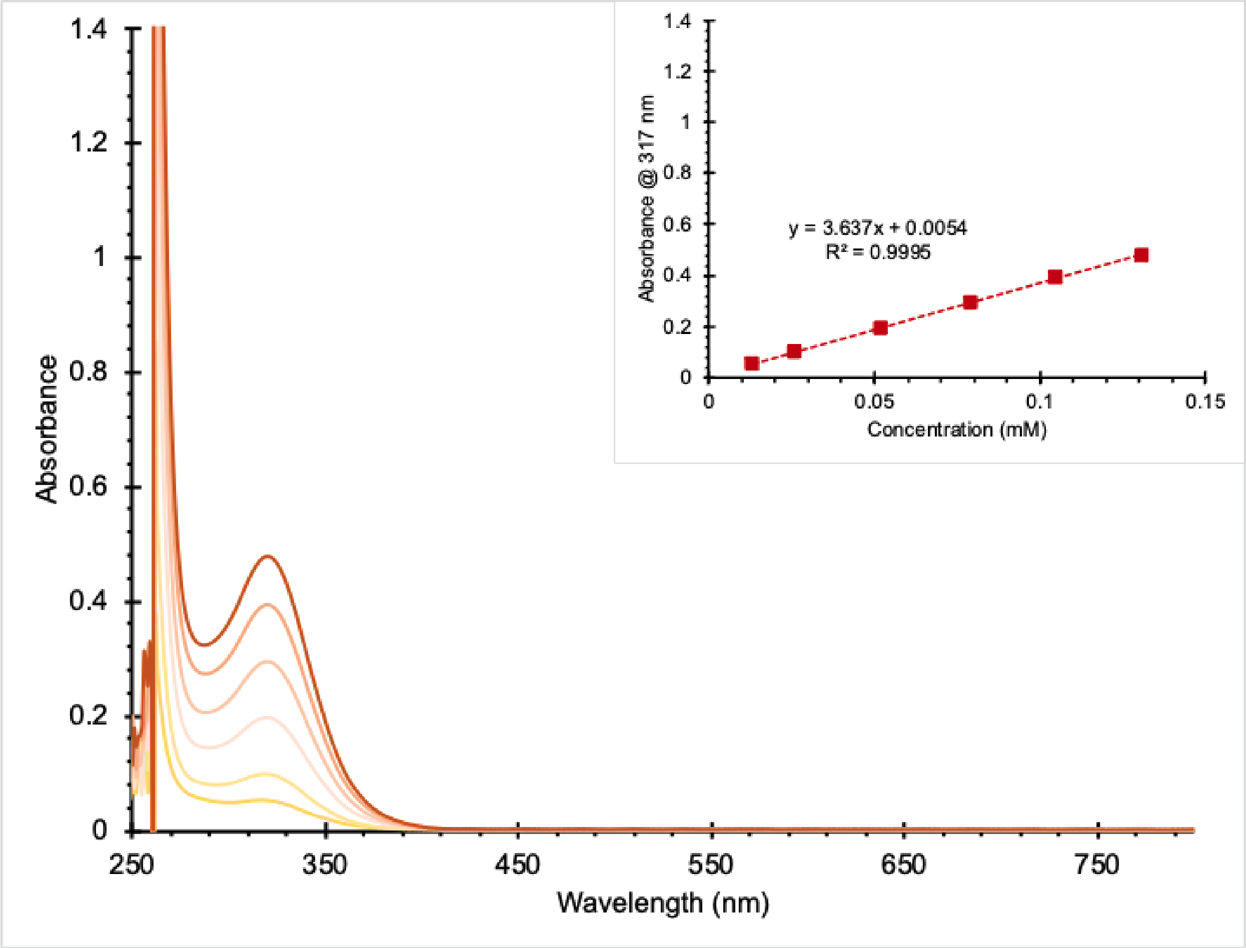

Figure S11. UV-Visible absorption spectra used to determine the molar absorptivity of **2** in DMAc.

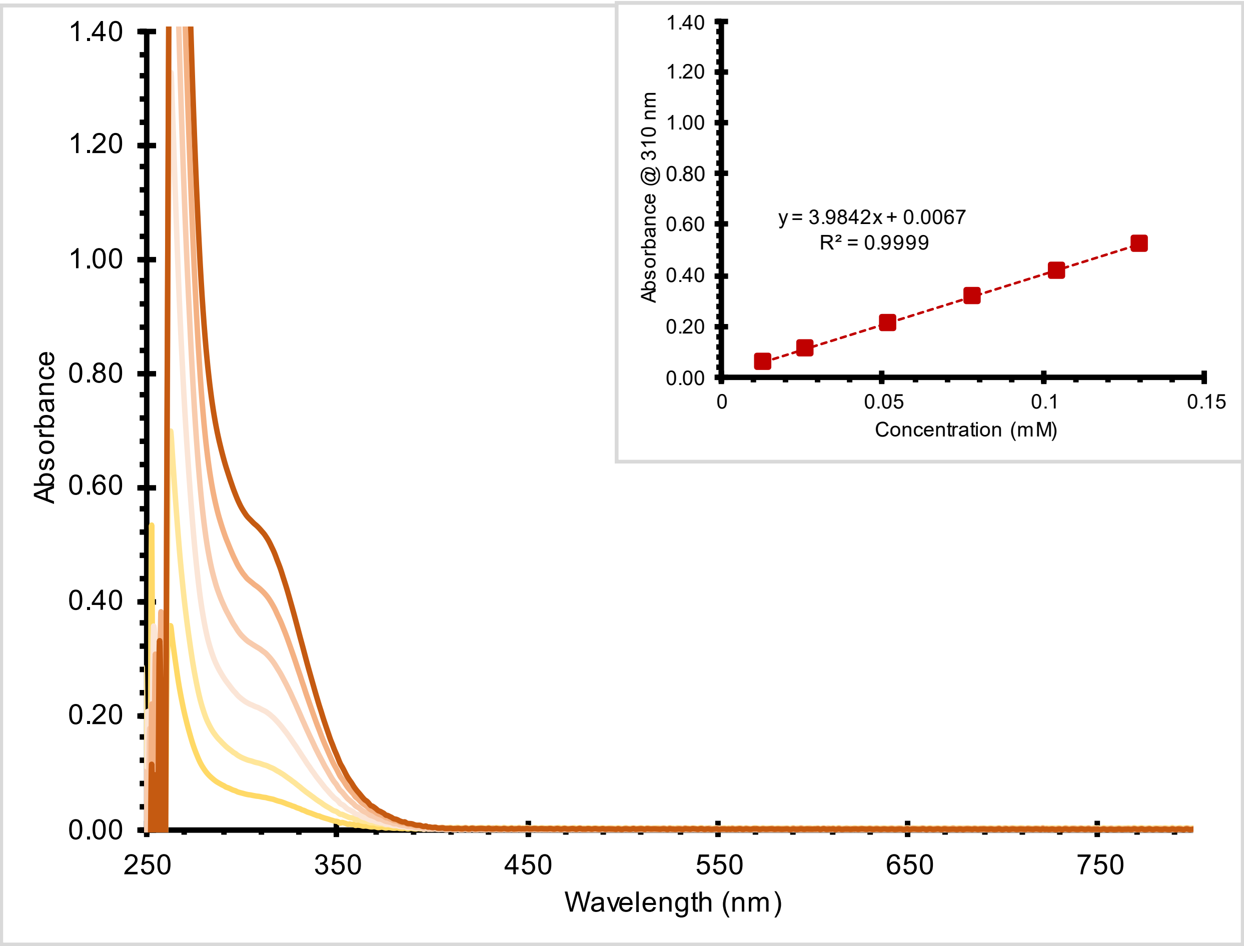

Figure S12. UV-Visible absorption spectra used to determine the molar absorptivity of **3** in DMAc.

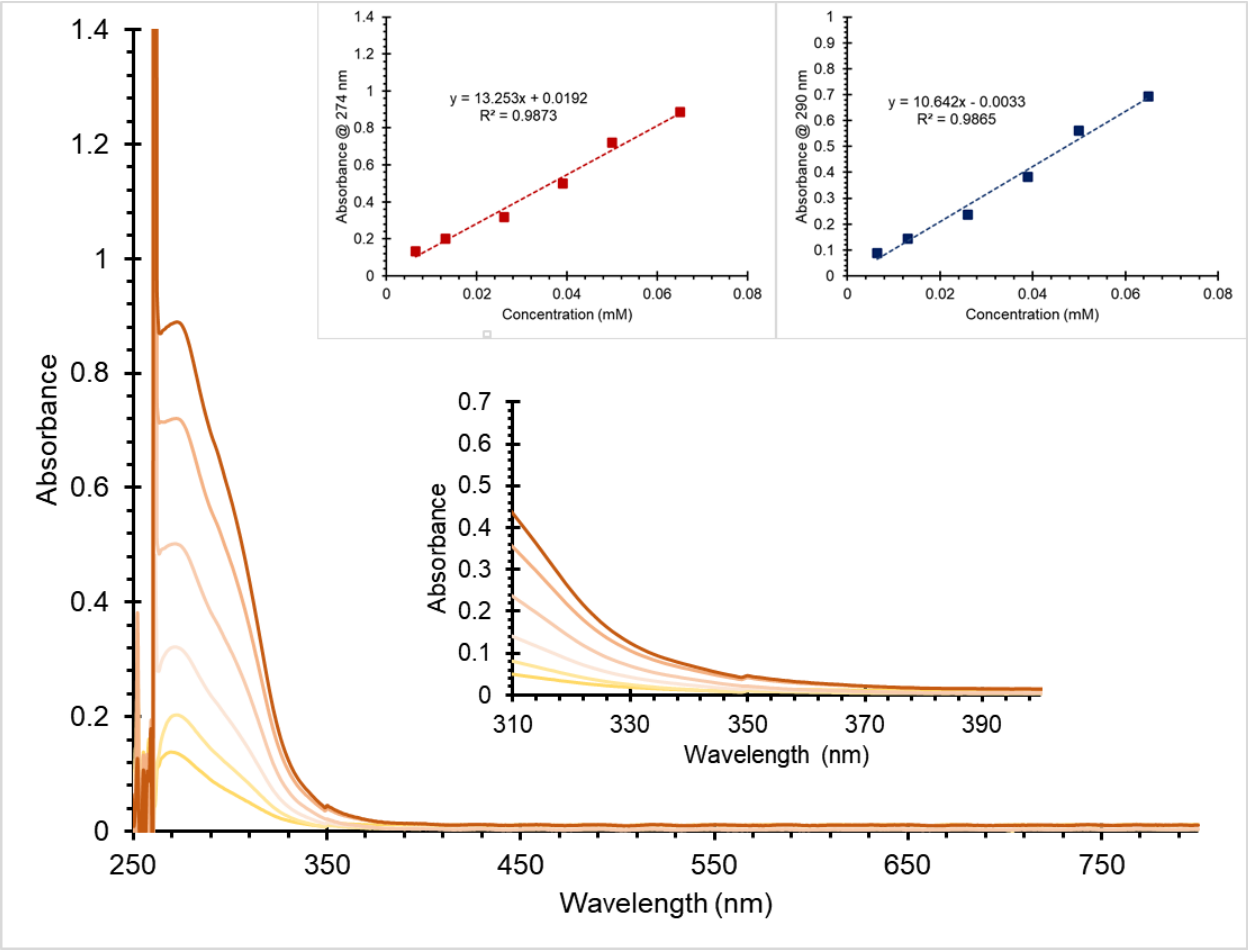

Figure S13. UV-Visible absorption spectra used to determine the molar absorptivity of **4** in DMAc.

Overlaid Spectra

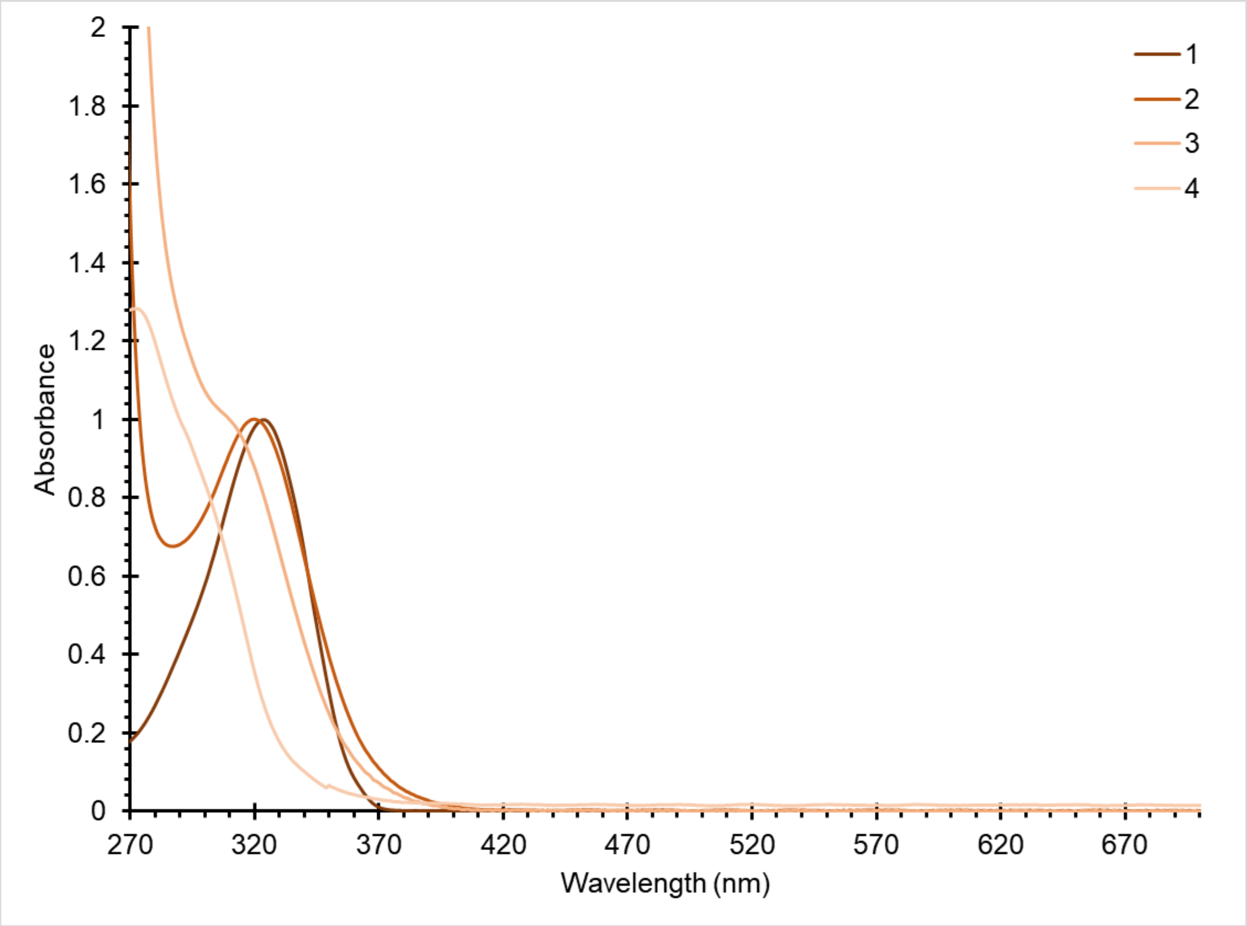

Figure S14. Overlaid UV-Vis absorption spectra of PC **1 – 4**.

Insights from Density Functional Theory

To gain insight into the orbitals involved in the absorption of light by each PC, density functional theory (DFT) calculations were performed to calculate the UV-Vis absorption spectrum and orbitals involved in absorption for PC **1** – **4**. For details on how these calculations were performed, see *Section 10. Computational Details and Data*.

In each case, the orbitals involved in absorption appear qualitatively similar in nature. These results are consistent with experimental data, which suggest a similar transition is present for each PC, but that this transition is blue-shifted to higher energies as the chalcogenide increases in size.

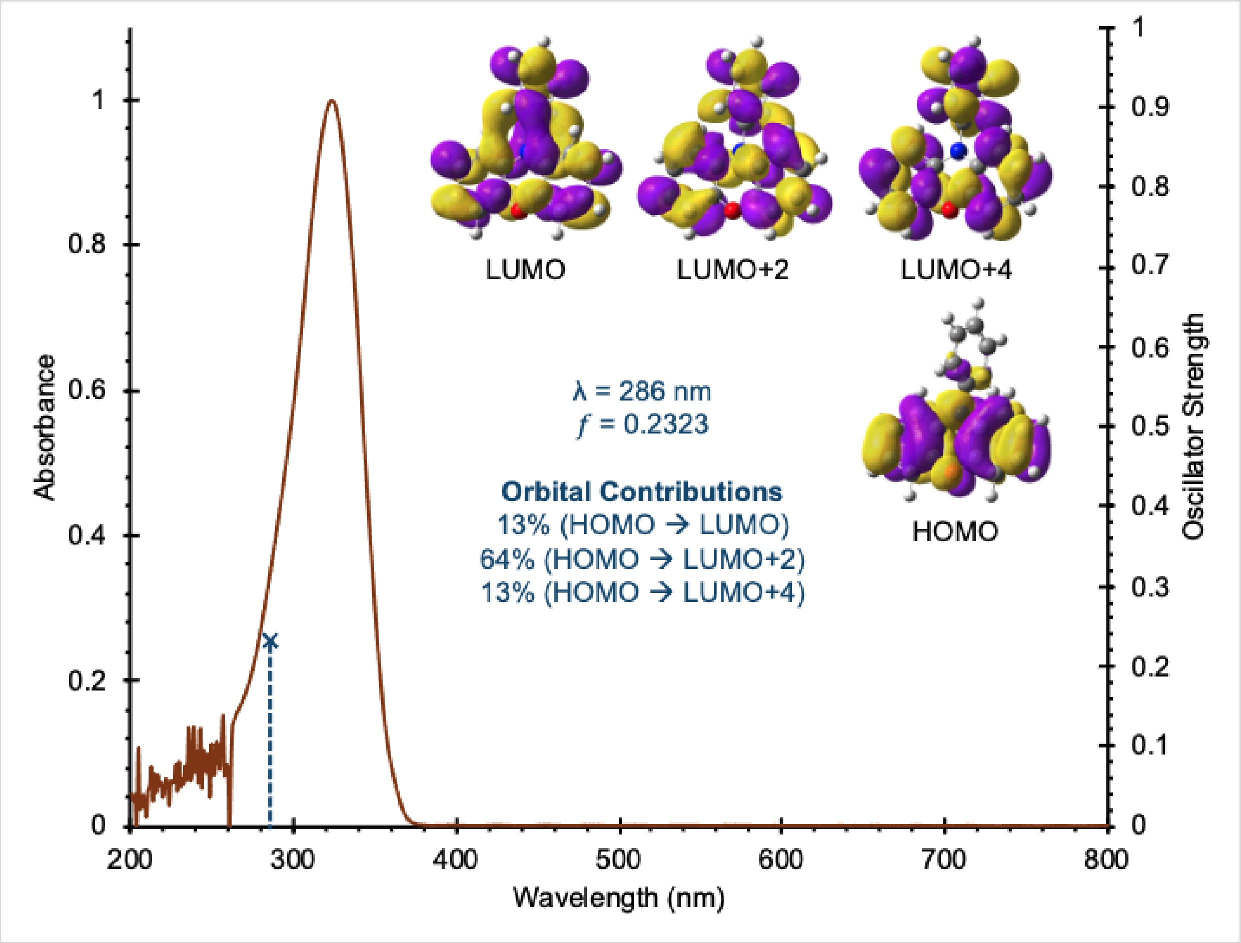

Figure S15. Computationally predicted electronic transition of **1** (dashed line) overlaid with the experimental absorption spectrum (solid line). Insets include orbital contributions for this transition and depictions of the orbitals involved.

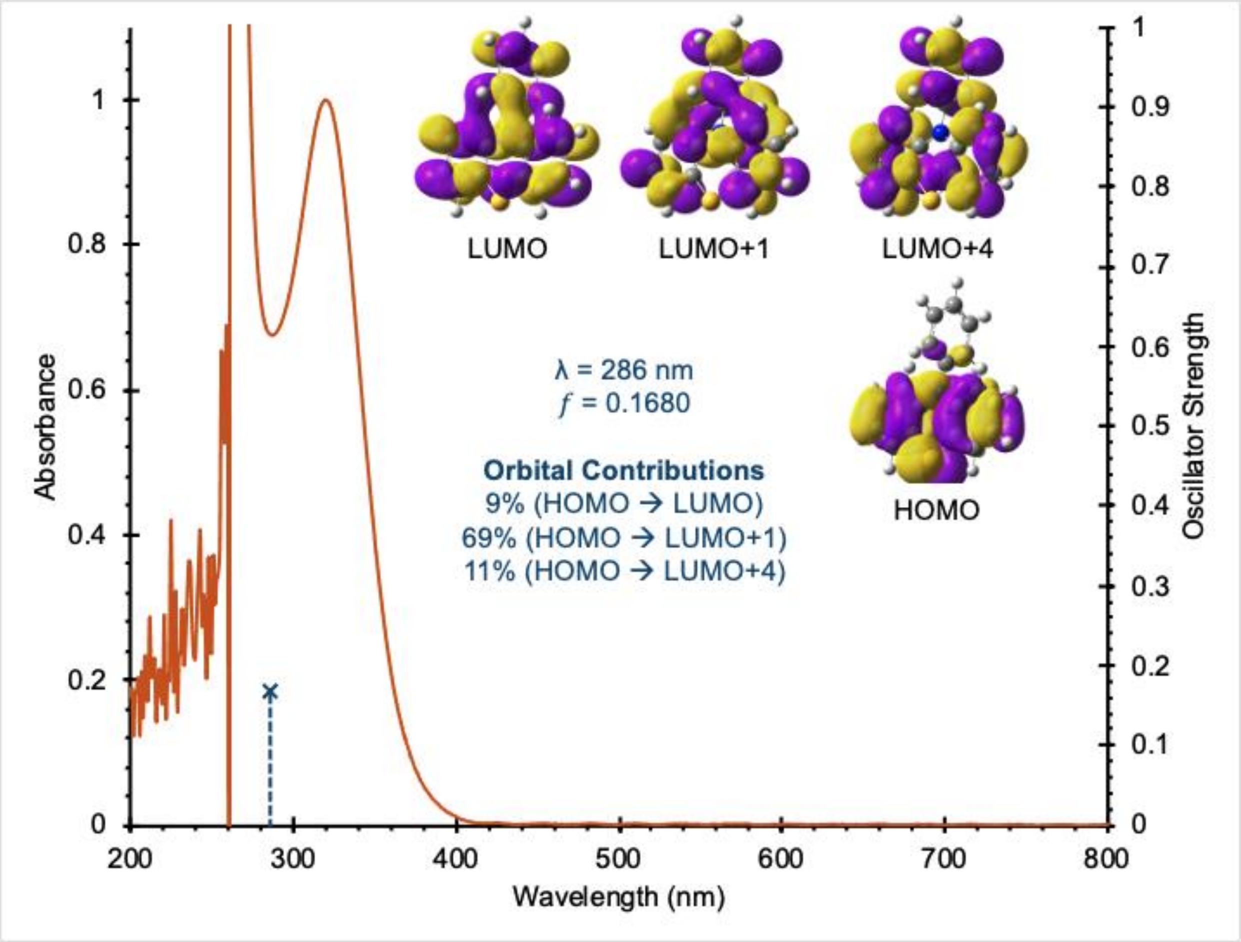

Figure S16. Computationally predicted electronic transition of **2** (dashed line) overlaid with the experimental absorption spectrum (solid line). Insets include orbital contributions for this transition and depictions of the orbitals involved.

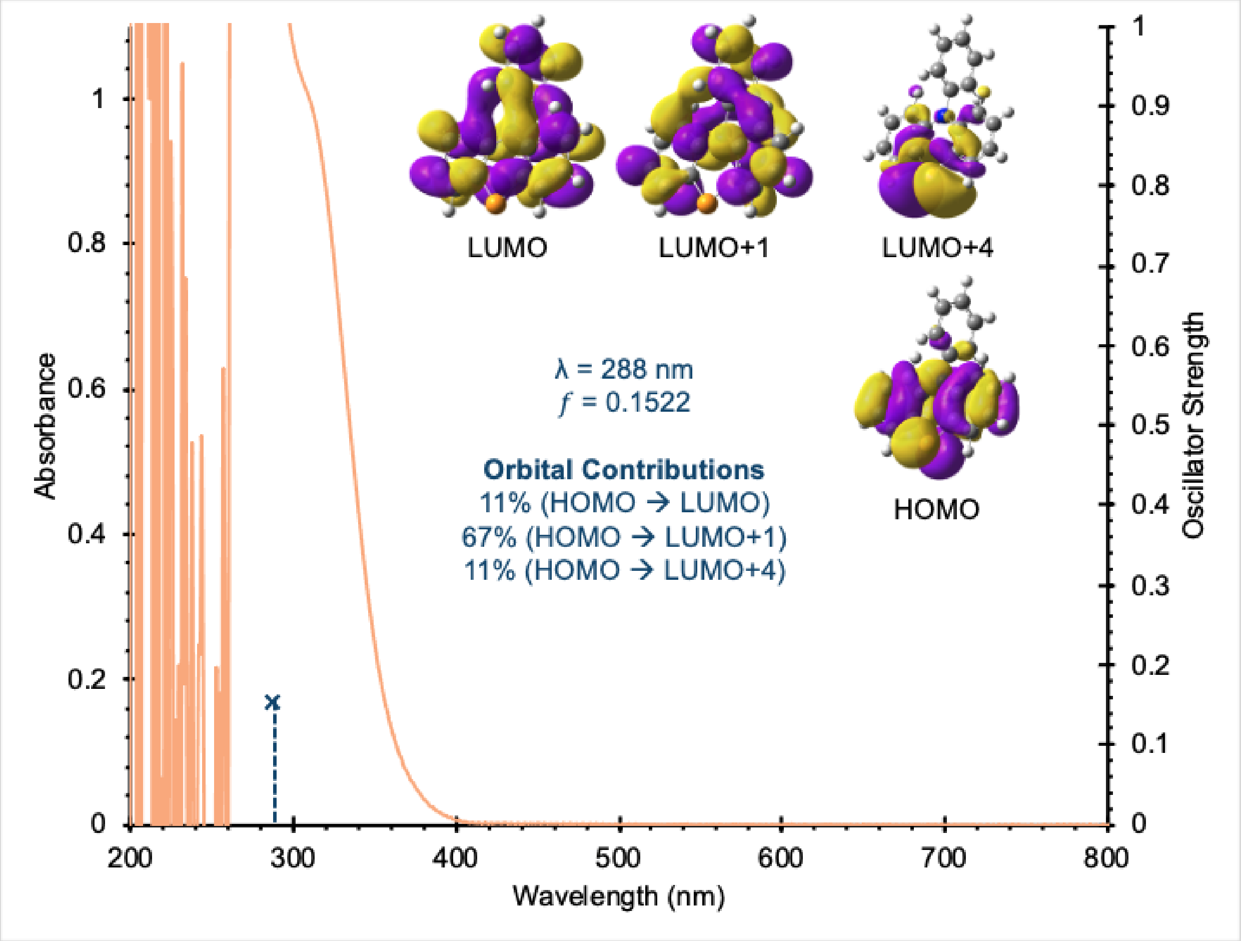

Figure S17. Computationally predicted electronic transition of **3** (dashed line) overlaid with the experimental absorption spectrum (solid line). Insets include orbital contributions for this transition and depictions of the orbitals involved.

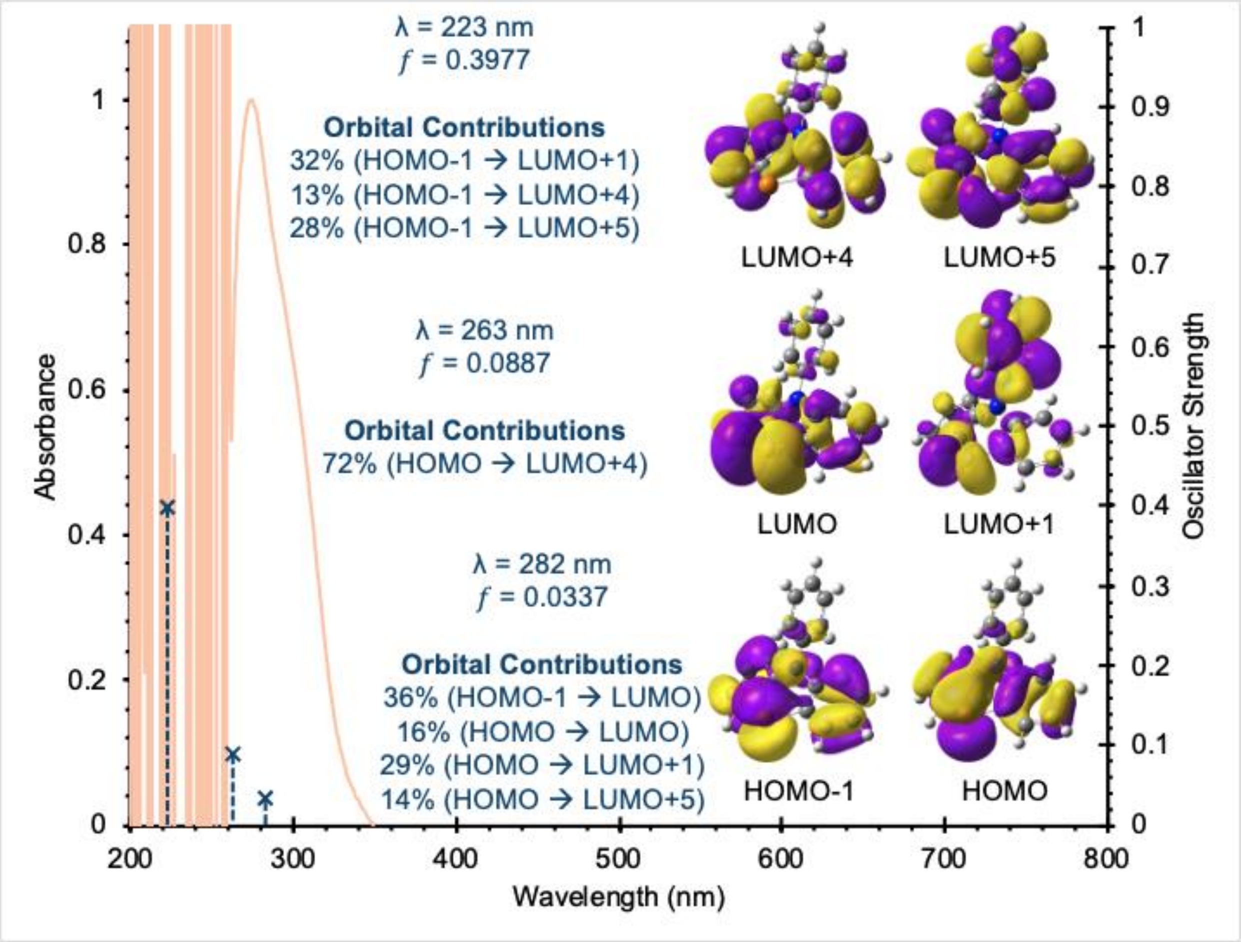

Figure S18. Computationally predicted electronic transition of **4** (dashed line) overlaid with the experimental absorption spectrum (solid line). Insets include orbital contributions for this transition and depictions of the orbitals involved.

4. Steady State Emission Spectroscopy

Fluorescence Spectroscopy

Solutions of each PC were prepared in three solvents of decreasing polarity (DMAc > tetrahydrofurane [THF] > 1-hexene) at an approximate concentration of 0.1 mM. The emission spectra of each solution were then measured. Where necessary, signal averaging (10 scans) was used to decrease noise in the emission spectra.

For PCs **3** and **4**, it was observed that the emission spectrum varied depending on the wavelength of excitation ($\lambda_{max,abs}$ or 355 nm). The cause of this variation is unknown and could simply be due to interference from the detection of the excitation light source. Nevertheless, the emission spectra of **1** and **2** were also collected with 355 nm excitation, although these spectra appeared consistent with those collected by excitation at $\lambda_{max,abs}$.

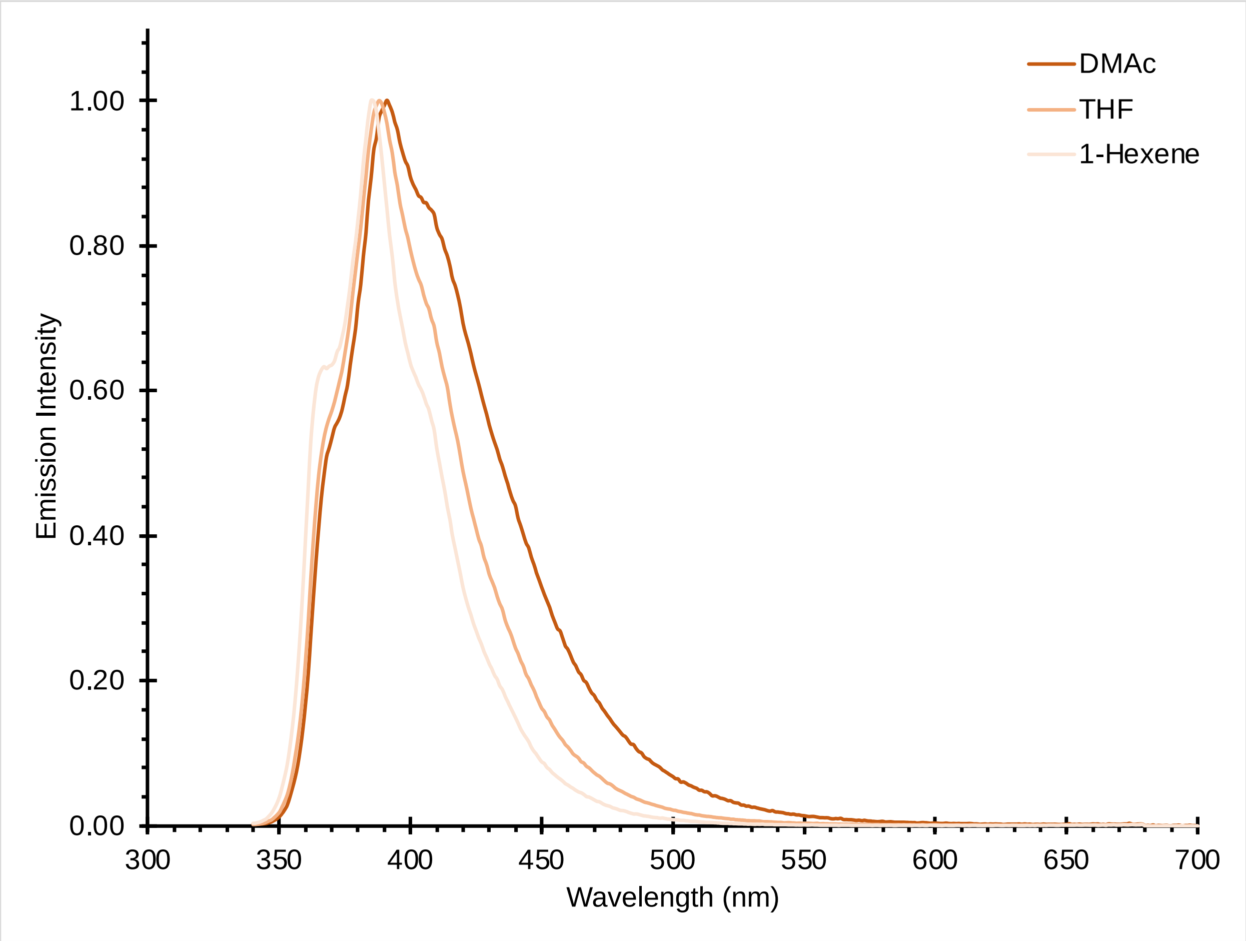

Figure S19. Fluorescence spectra of **1** excited at $\lambda_{max,abs}$.

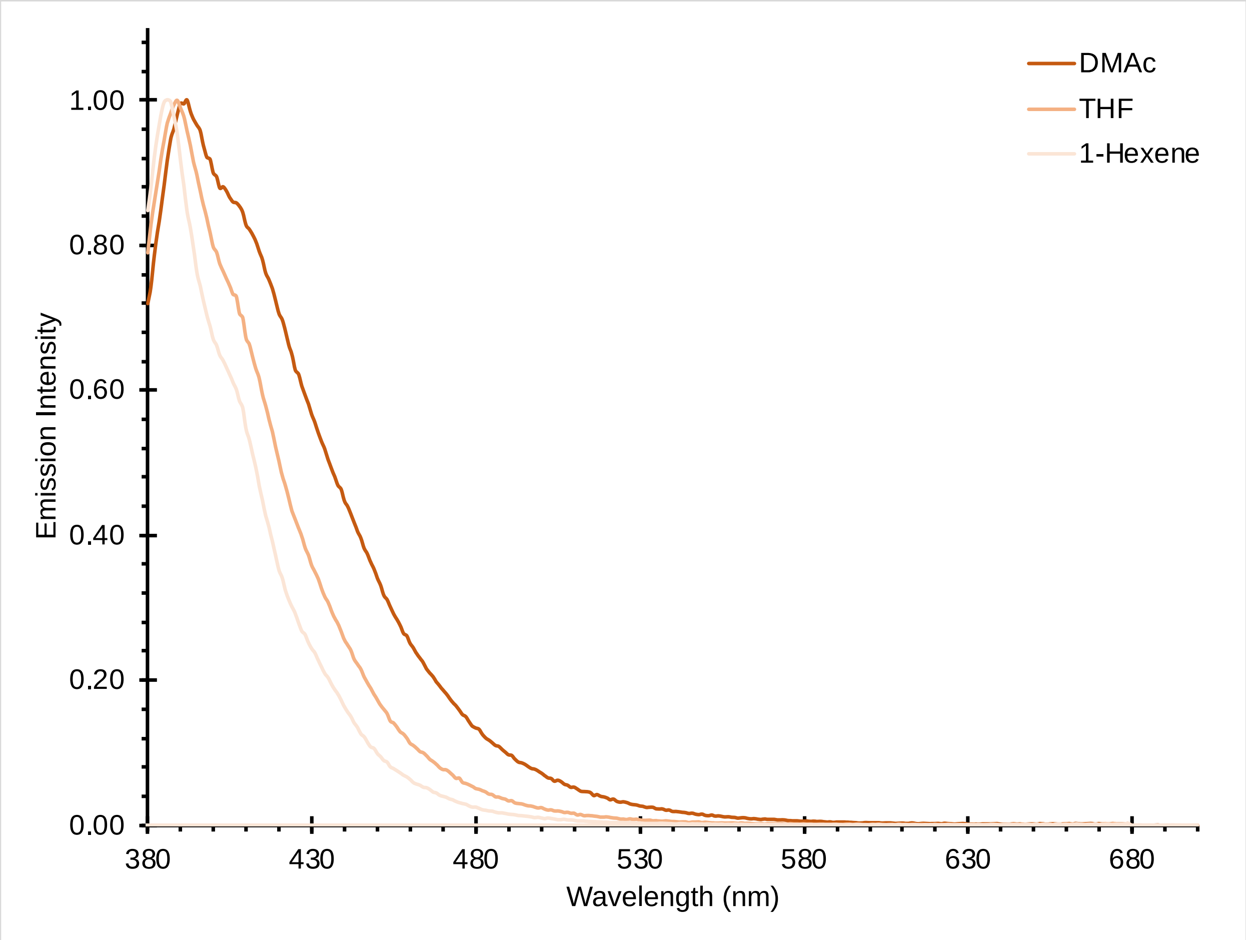

Figure S20. Fluorescence spectra of **1** excited at 355 nm.

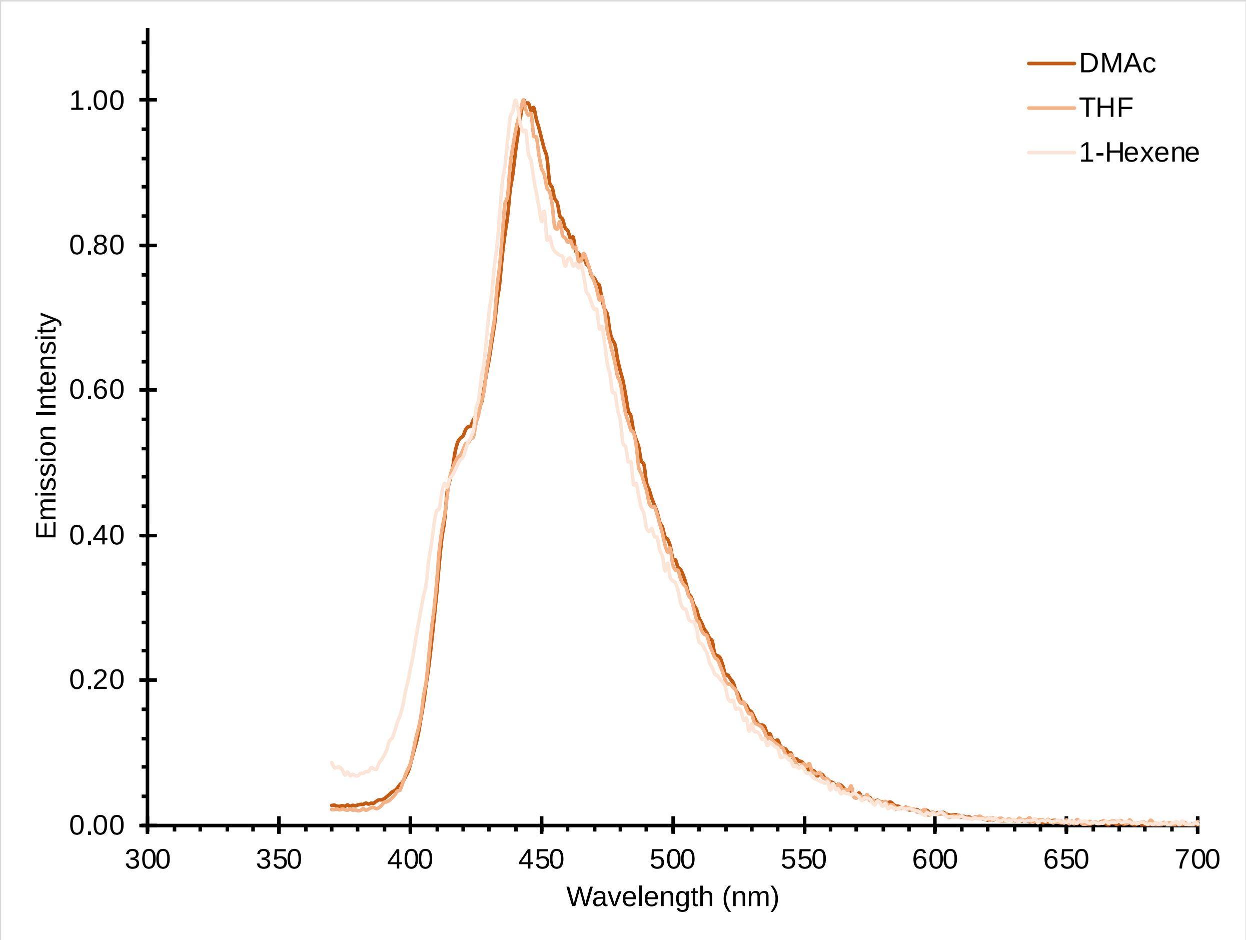

Figure S21. Fluorescence spectra of **2** excited at $\lambda_{max,abs}$.

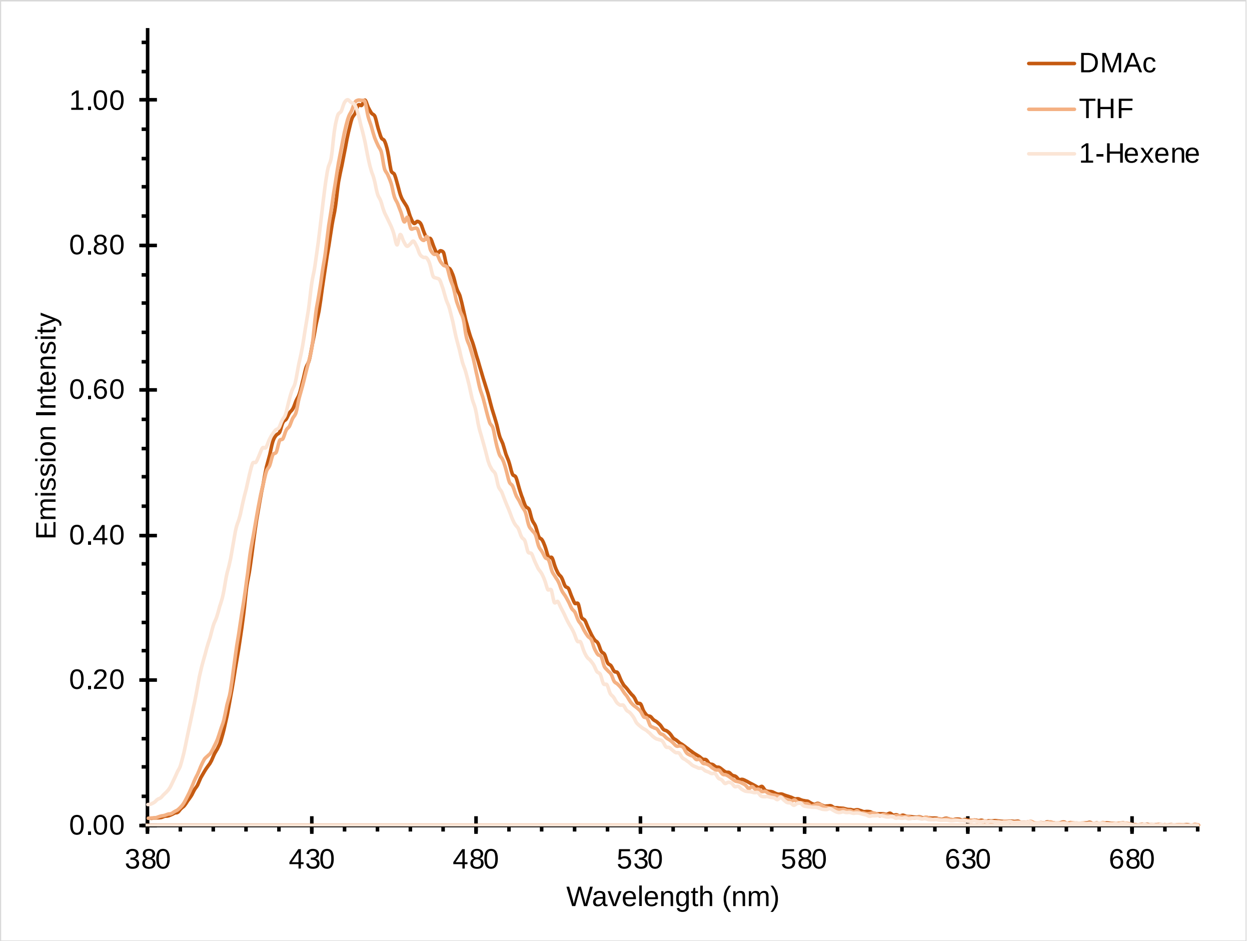

Figure S22. Fluorescence spectra of **2** excited at 355 nm.

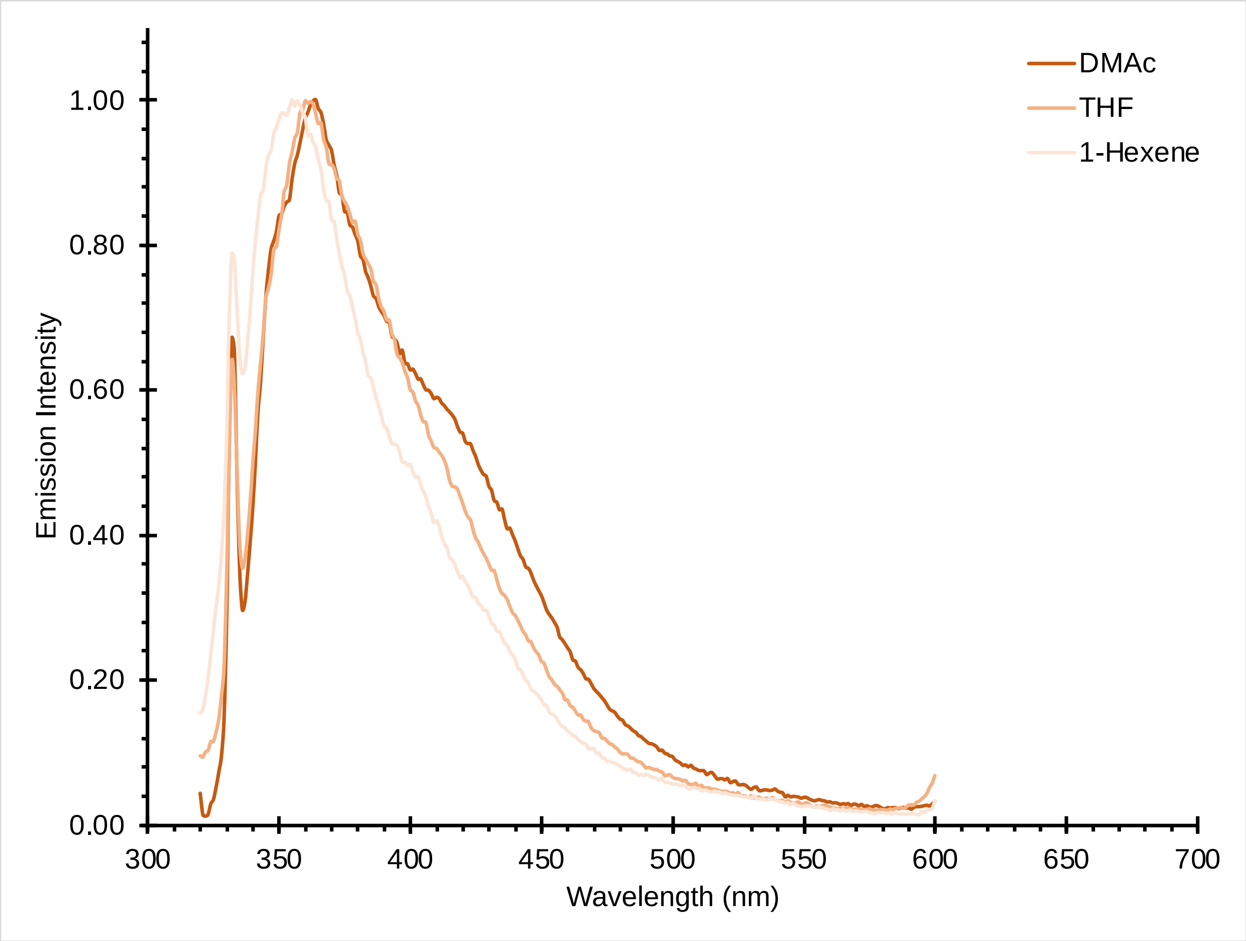

Figure S23. Fluorescence spectra of **3** excited at 310 nm.

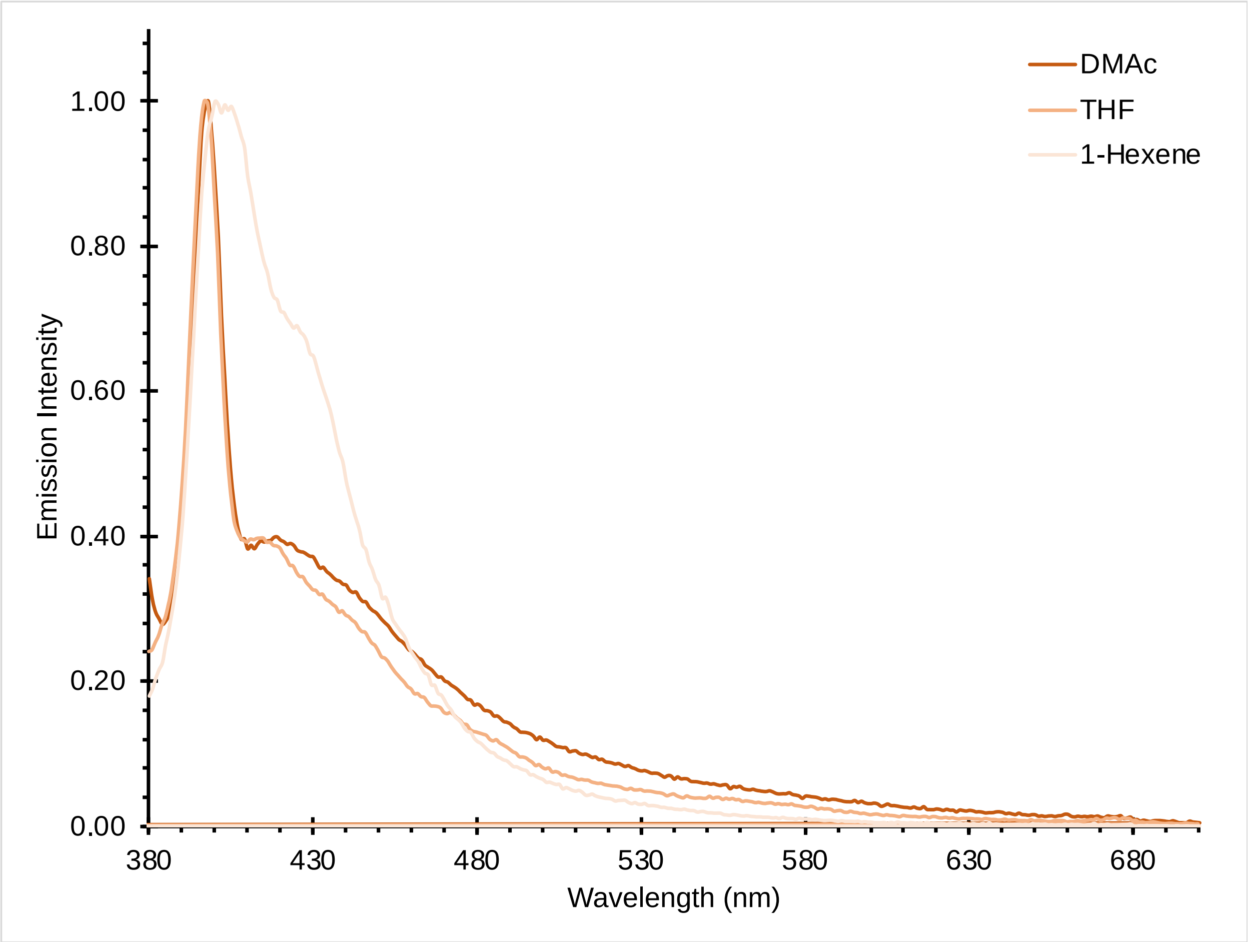

Figure S24. Fluorescence spectra of **3** excited at 355 nm.

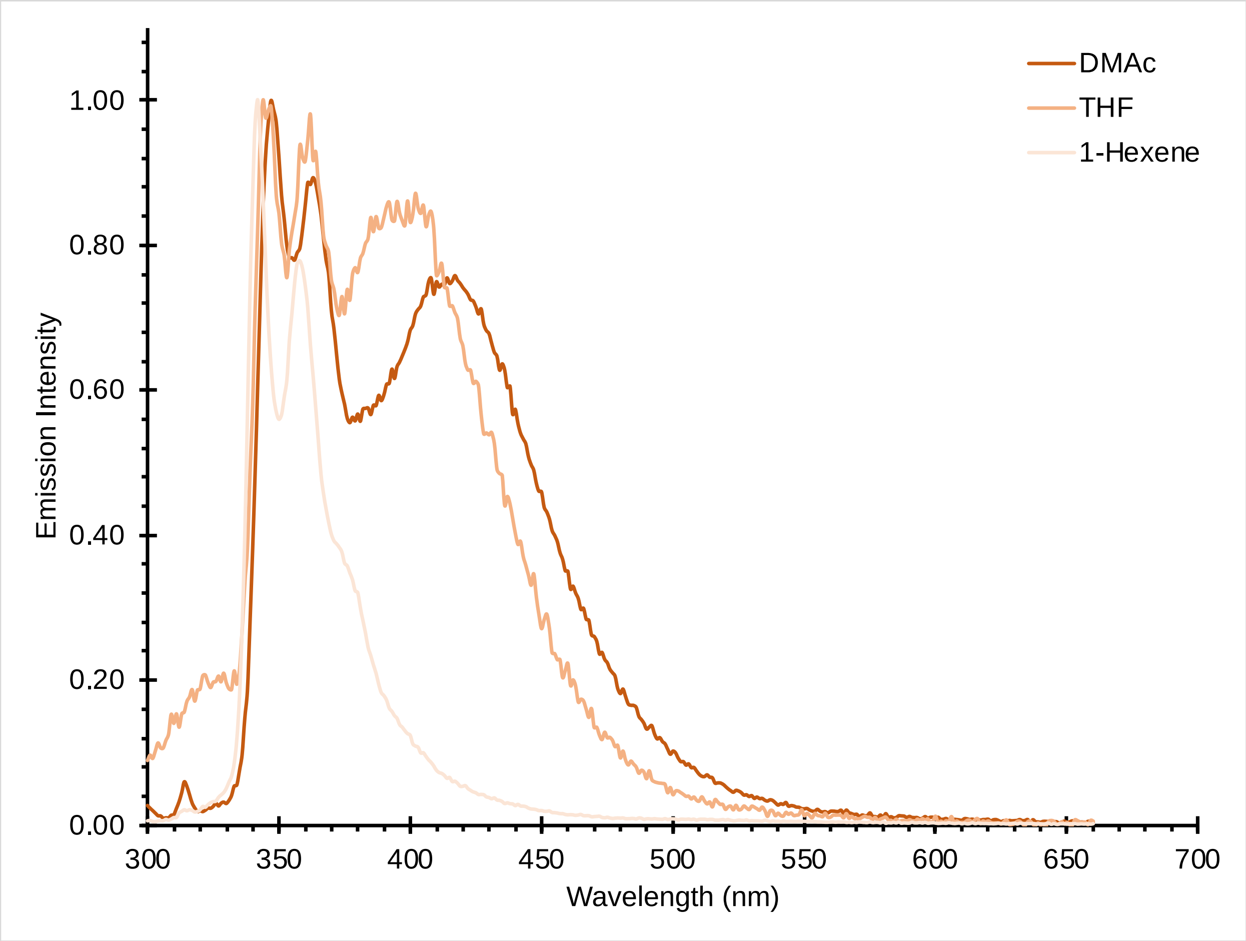

Figure S25. Fluorescence spectra of **4** excited at 288 nm.

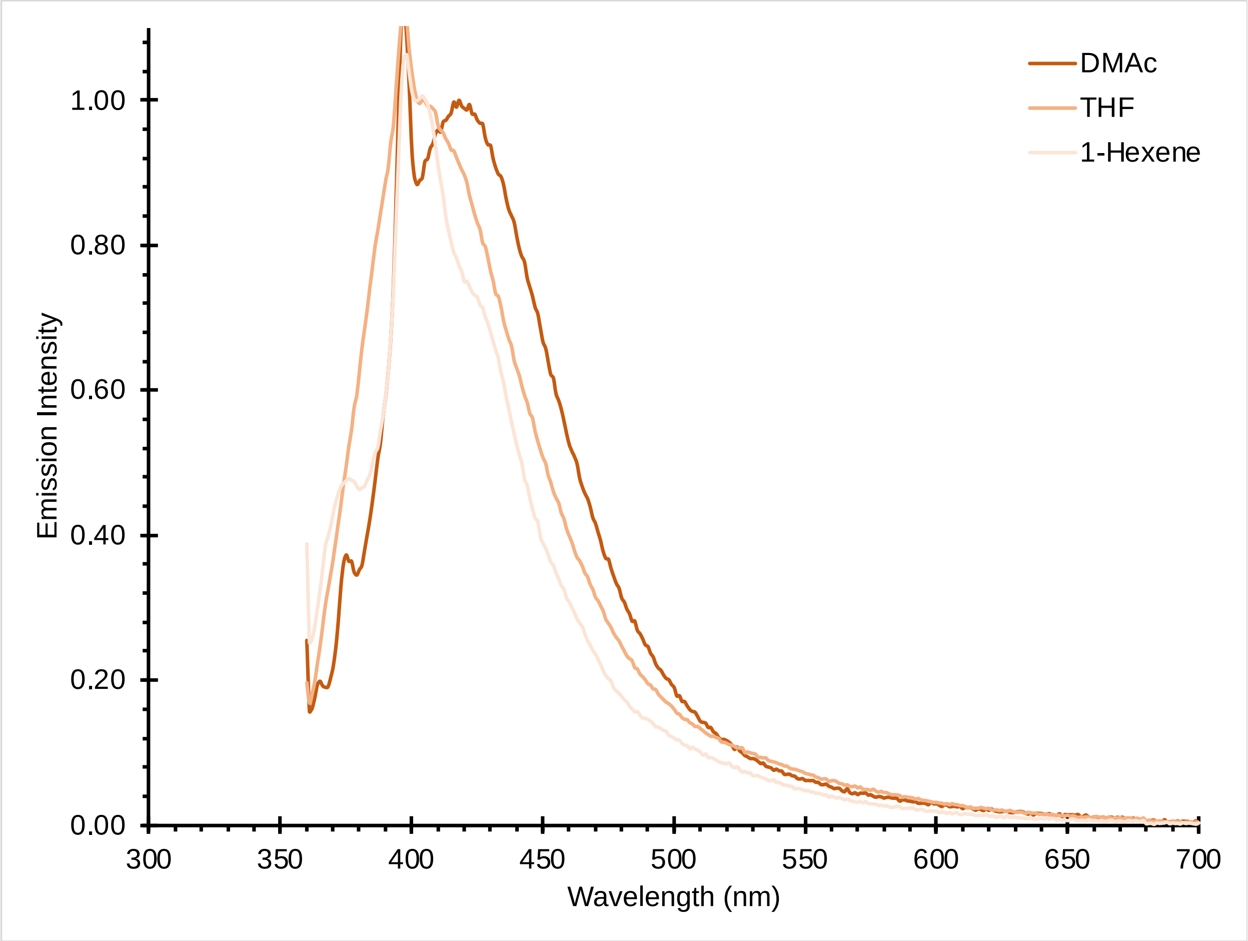

Figure S26. Fluorescence spectra of **4** excited at 355 nm.

Phosphorescence Spectroscopy

The phosphorescence spectra of PC **1 – 4** were collected in the following manner: a 0.1 mM solution of each PC was prepared in *N,N*-dimethylformamide (DMF) in a nitrogen filled glovebox and transferred to an NMR tube. The tube was then sealed, the cap wrapped with parafilm to minimize oxygen diffusion into the solution, and the sample was removed from the glovebox. The sample was excited with a 355 nm laser and its emission spectrum measured at room temperature with a 0 ns gate delay (100 ns gate width) or a 1 ms gate delay (30 ms gate width). In every case, measurements with a 1 ms gate delay at room temperature showed no emission signal, consistent with a lack of phosphorescence at room temperature. The samples were then cooled to 77 K and remeasured under the same conditions (with a 0 ns or 1 ms gate delay).

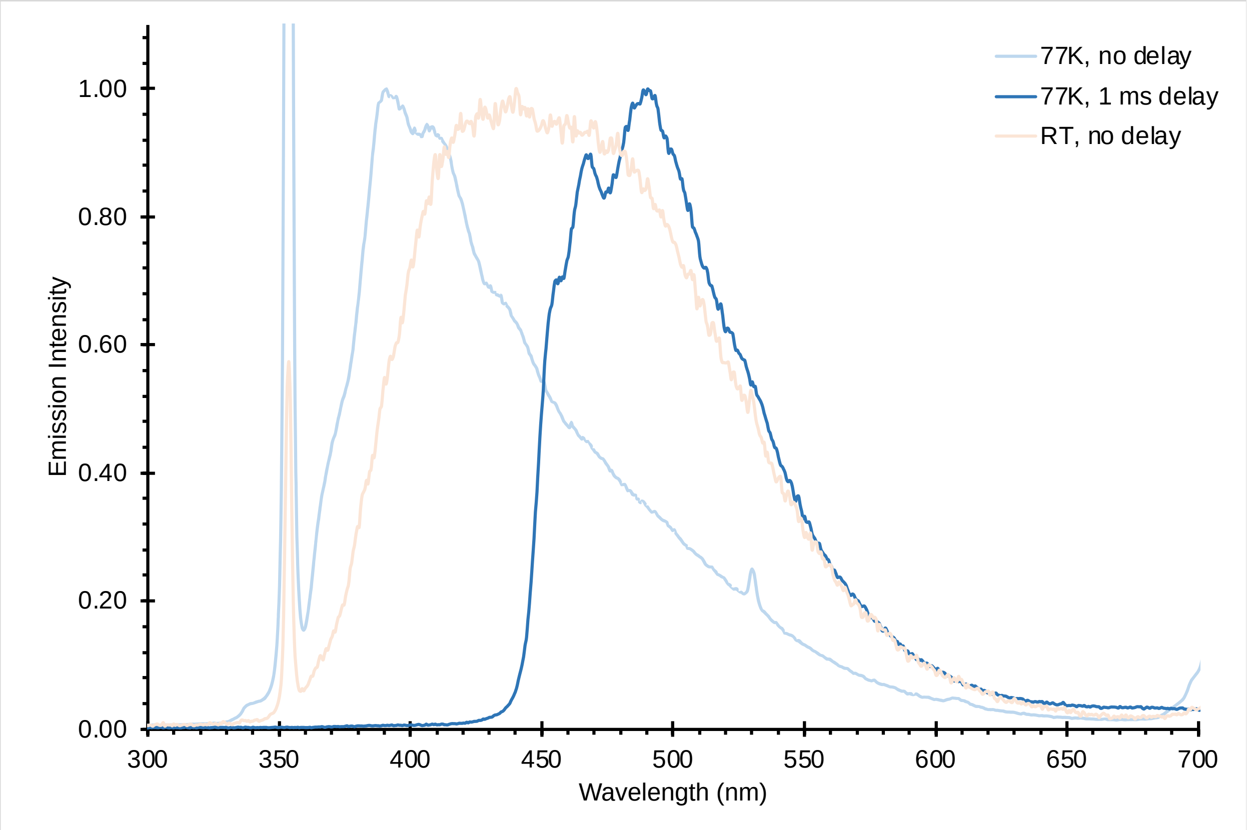

Figure S27. Room temperature fluorescence (orange), low temperature fluorescence (light blue), and low temperature phosphorescence (dark blue) spectra of PC **1**.

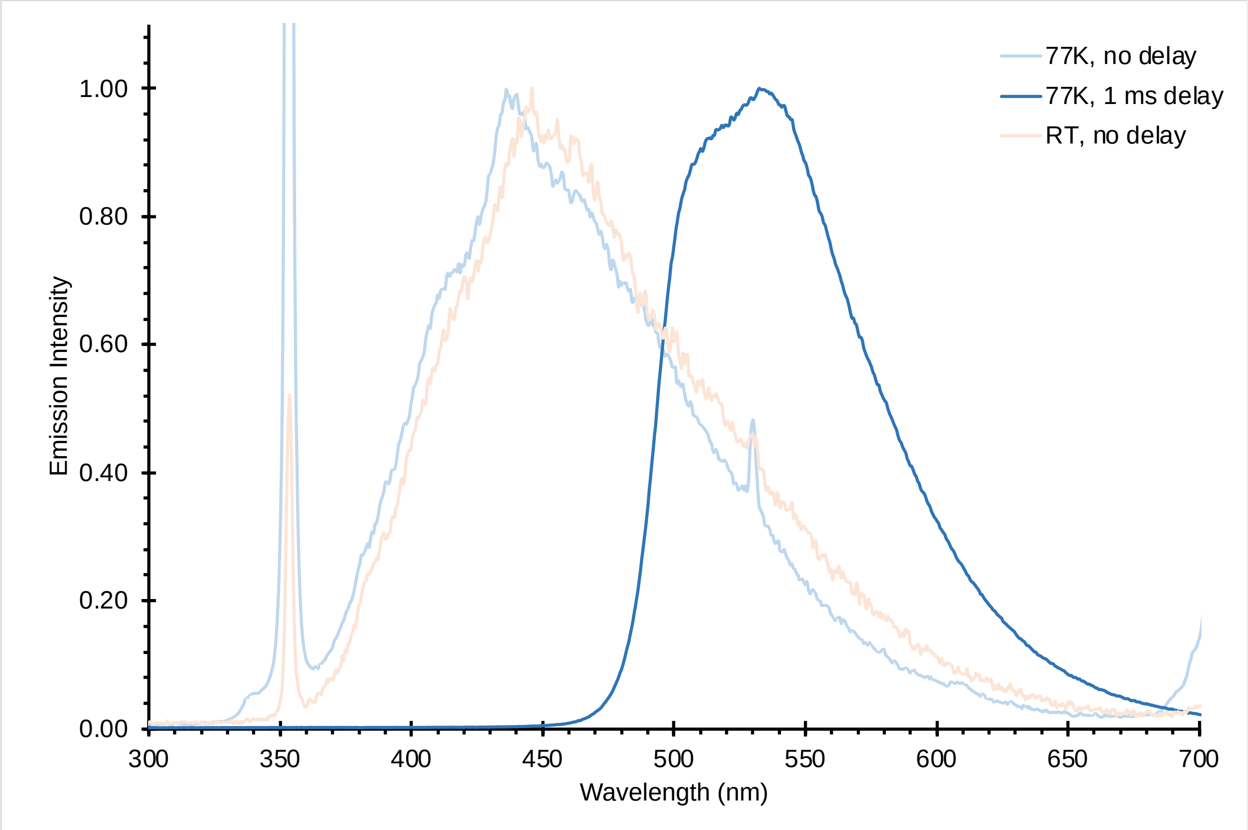

Figure S28. Room temperature fluorescence (orange), low temperature fluorescence (light blue), and low temperature phosphorescence (dark blue) spectra of PC **2**.

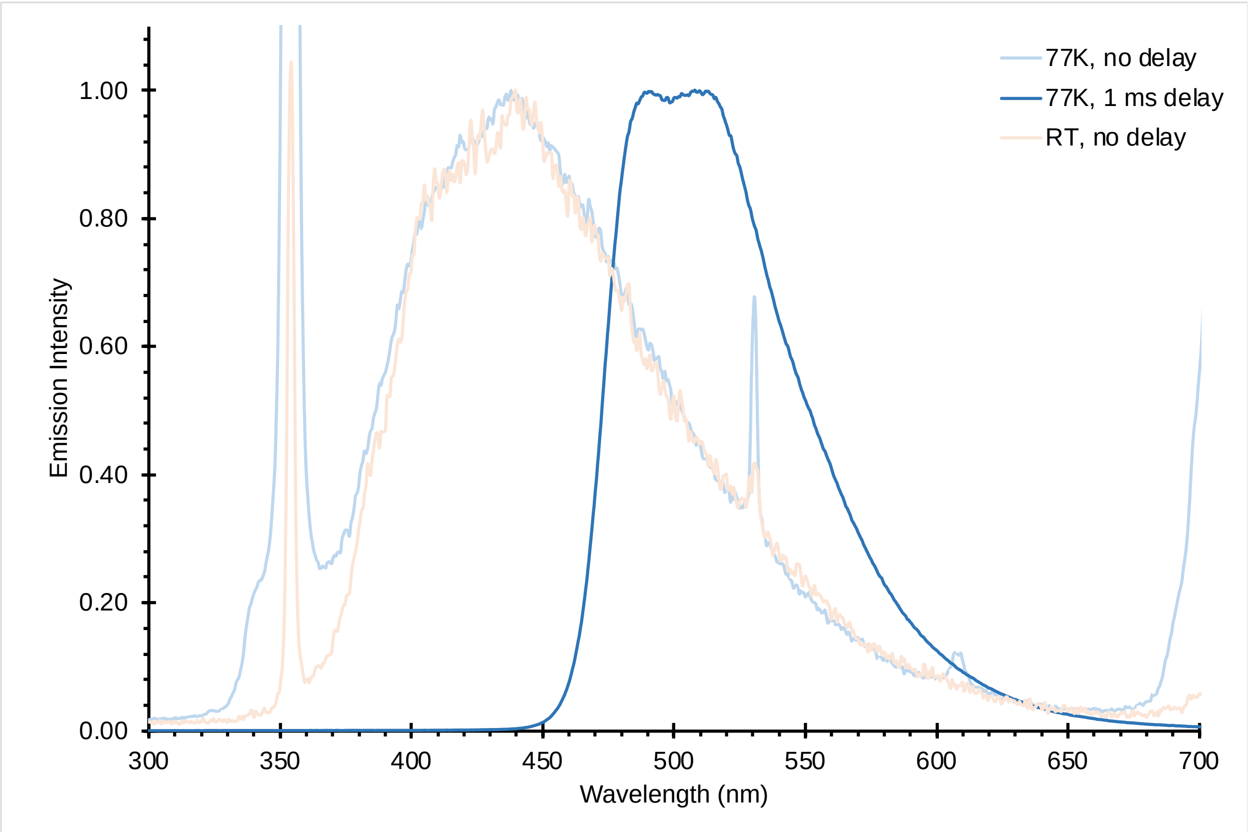

Figure S29. Room temperature fluorescence (orange), low temperature fluorescence (light blue), and low temperature phosphorescence (dark blue) spectra of PC **3**.

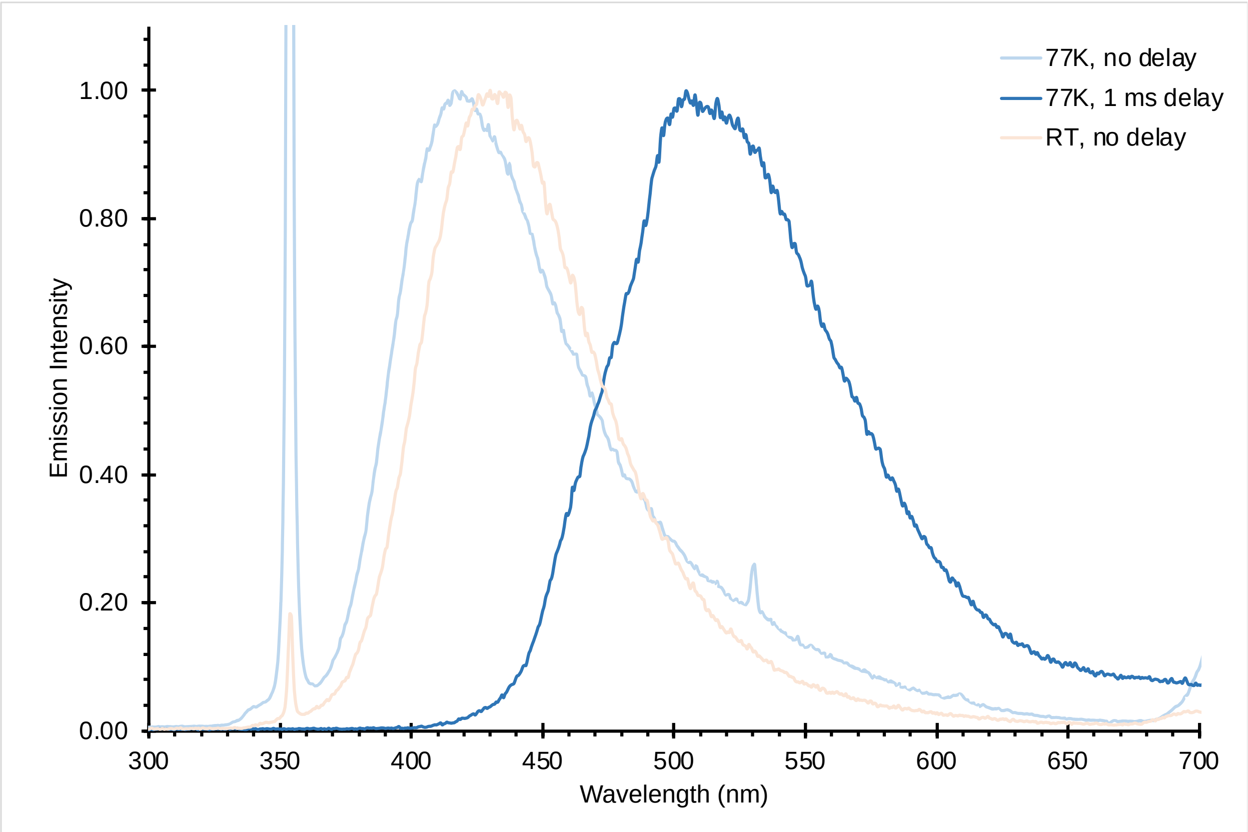

Figure S30. Room temperature fluorescence (orange), low temperature fluorescence (light blue), and low temperature phosphorescence (dark blue) spectra of PC **4**.

Stern-Volmer Quenching Experiments

To measure the rate of activation from the singlet excited state, steady-state Stern-Volmer quenching experiments were performed. In each case, a 0.1 mM solution of the PC was prepared in DMAc in a nitrogen filled glovebox. A part of this solution (3 mL) was transferred to an air-free cuvette equipped with a Kontes valve. For measurements in the presence of quencher, 100 to 400 equivalents of DBMM was added to the cuvette using a Hamilton syringe. The cuvette was then sealed, inverted to stir the solution, and transported to the fluorescence spectrometer for measurement. For each PC, this process was repeated in triplicate at each concentration of quencher. The data was then analyzed according to the Stern-Volmer relationship (Equation S1).

$$\frac{I^o}{I} = 1 + k_q \tau_o [Q] \qquad \text{Eq. (S1)}$$

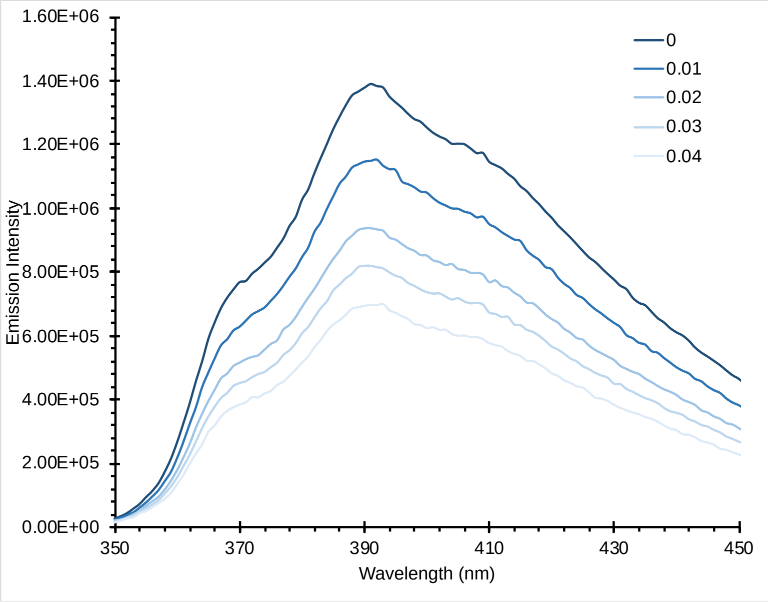

Figure S31. Emission spectra of PC **1** in the presence of 0 to 400 equivalents (0 M to 0.04 M) of DBMM as a quencher.

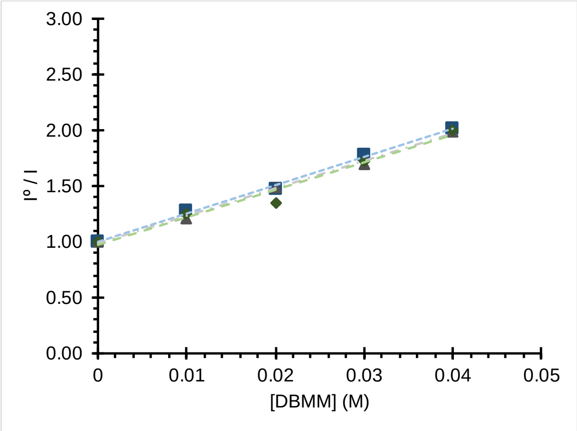

Figure S32. Stern-Volmer analysis for PC **1**.

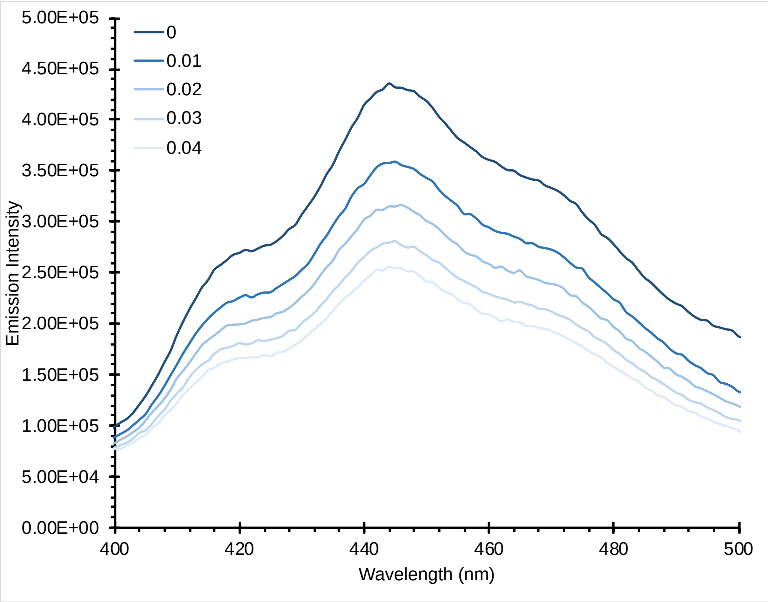

Figure S33. Emission spectra of PC **2** in the presence of 0 to 400 equivalents (0 M to 0.04 M) of DBMM as a quencher.

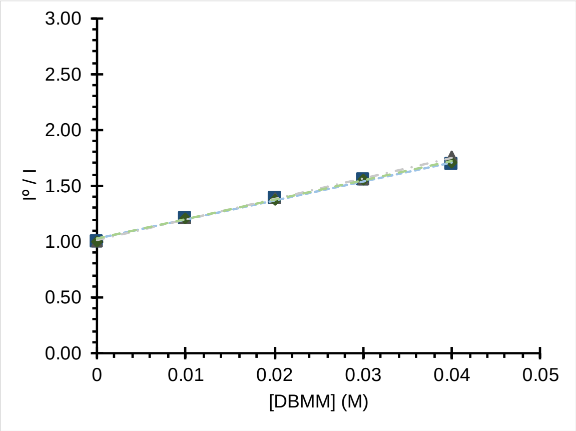

Figure S34. Stern-Volmer analysis for PC **2**.

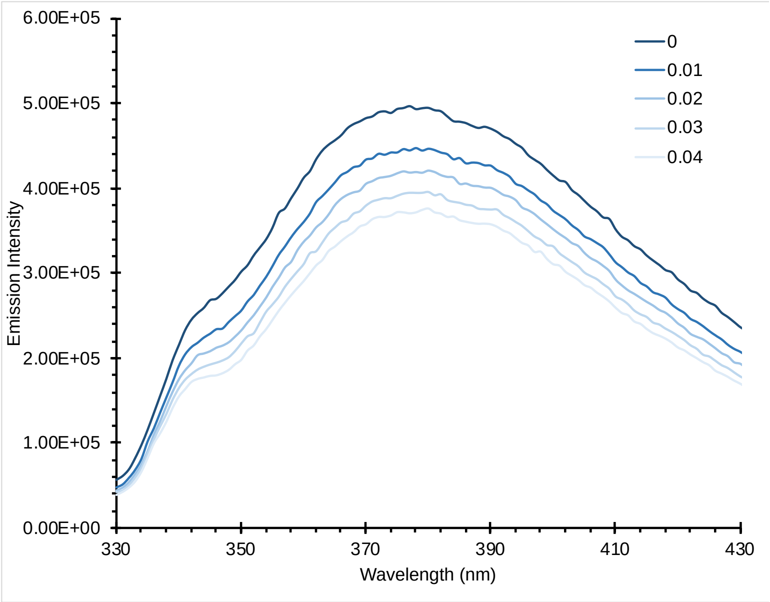

Figure S35. Emission spectra of PC **3** in the presence of 0 to 400 equivalents (0 M to 0.04 M) of DBMM as a quencher.

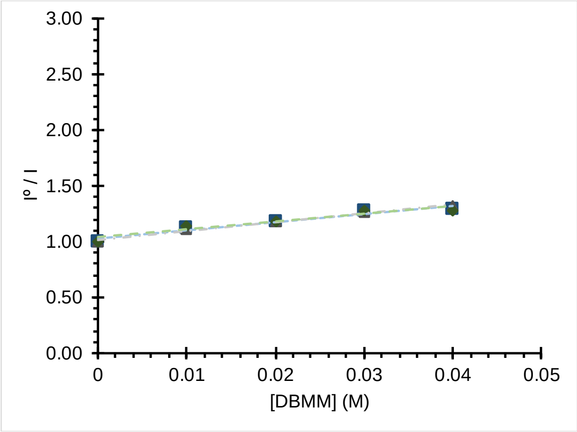

Figure S36. Stern-Volmer analysis for PC **3**.

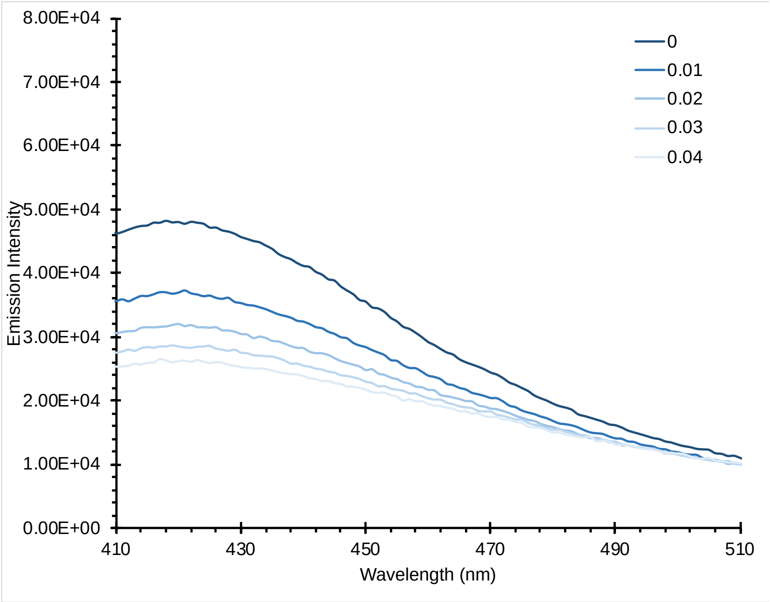

Figure S37. Emission spectra of PC **4** in the presence of 0 to 400 equivalents (0 M to 0.04 M) of DBMM as a quencher.

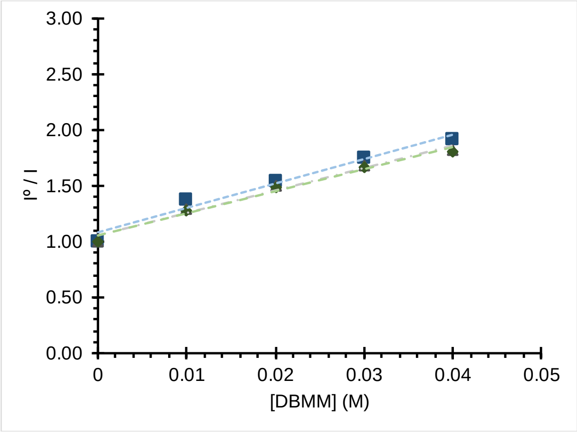

Figure S38. Stern-Volmer analysis for PC **4**.

5. Fluorescence Quantum Yield Measurements

Fluorescence quantum yield measurements were performed using the direct excitation method with an integrating sphere. In each case, solutions of PCs **1 – 4** were prepared with A < 0.2 to avoid the inner filter effect. Both PC solutions and solvent blanks were prepared in a nitrogen filled glovebox with DMAc as the solvent. Unless otherwise noted, the $\lambda_{max,abs}$ was used as the excitation wavelength.

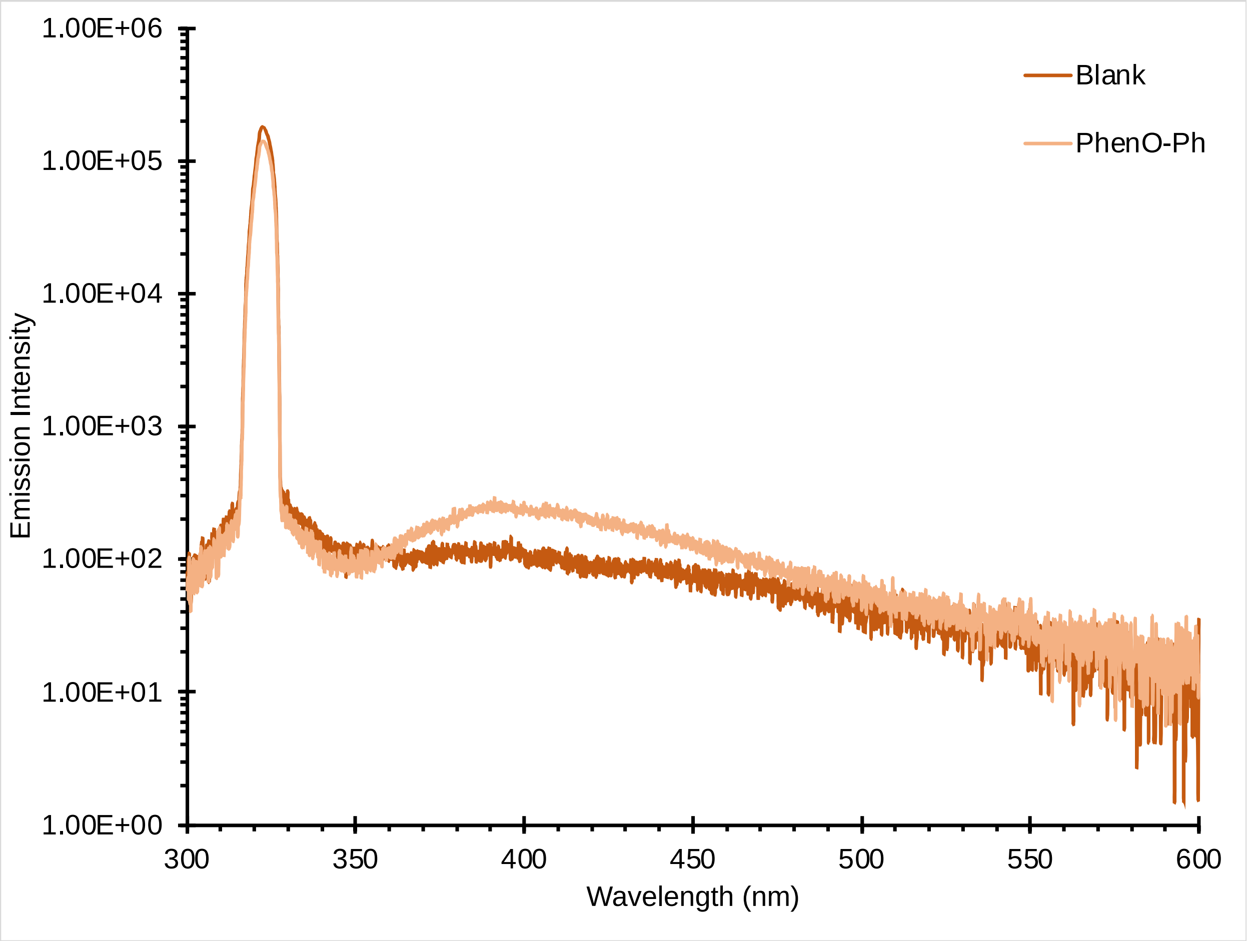

Figure S39. Emission spectra of **1** used for the measurement of fluorescence quantum yield.

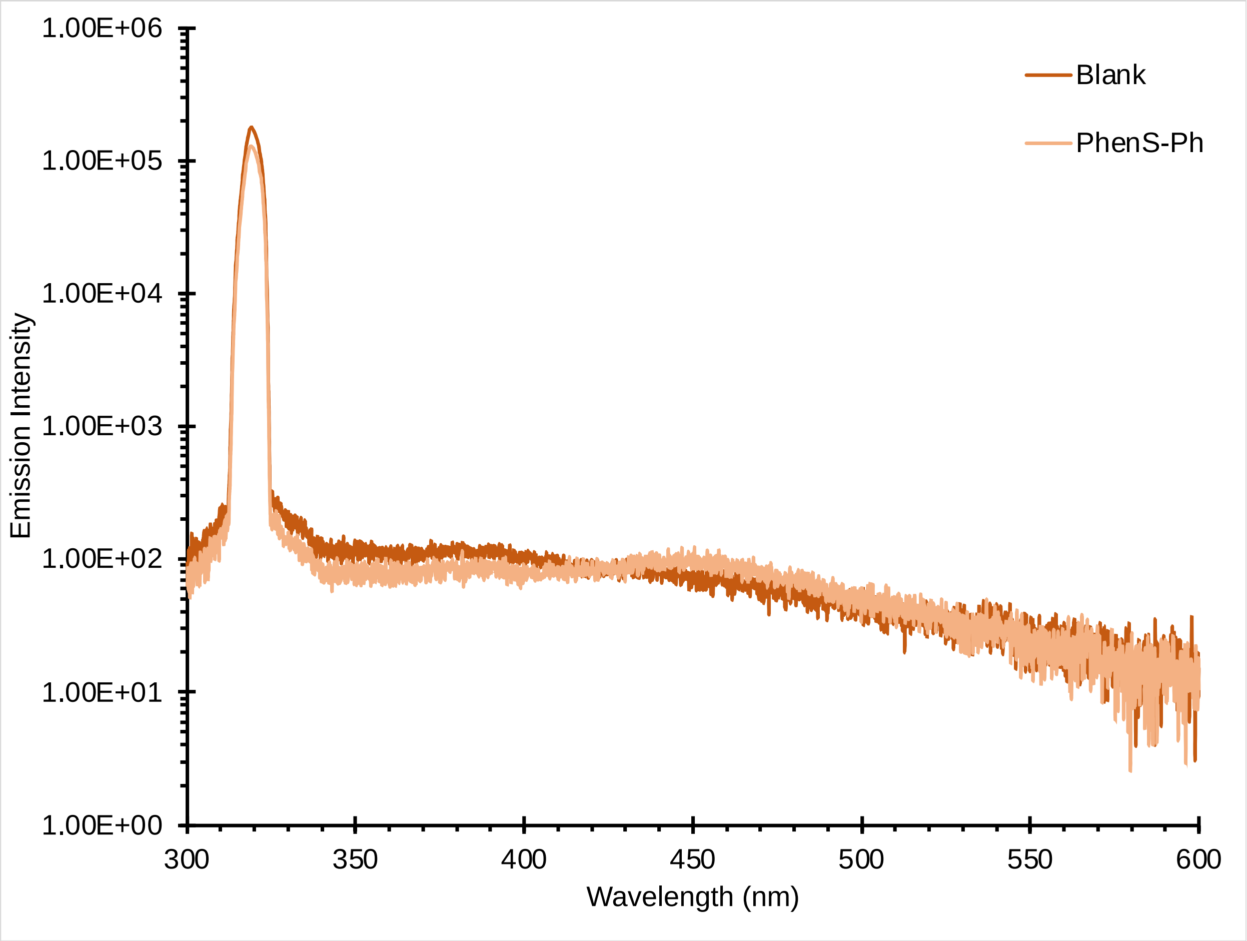

Figure S40. Emission spectra of **2** used for the measurement of fluorescence quantum yield.

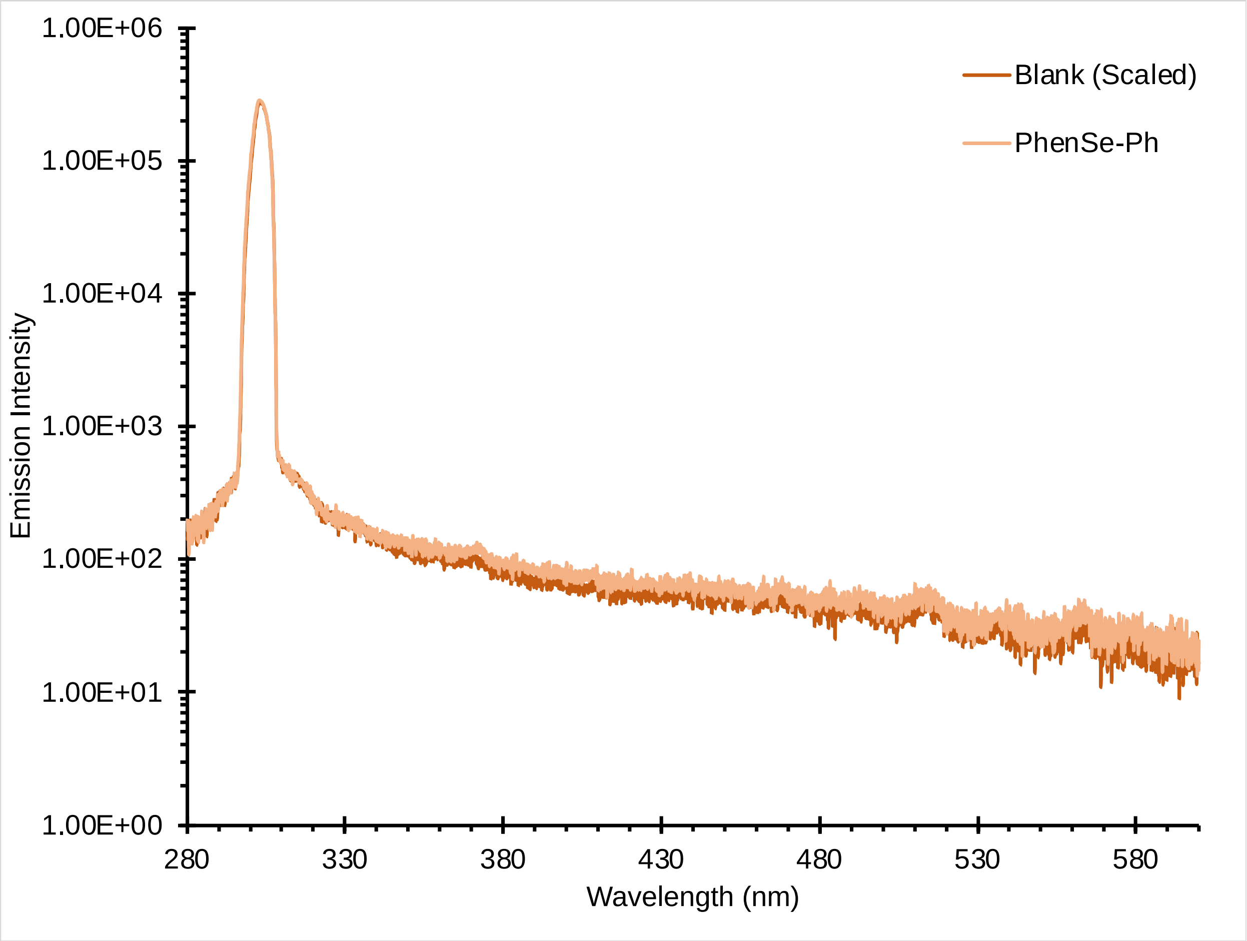

Figure S41. Emission spectra of **3** used for the measurement of fluorescence quantum yield.

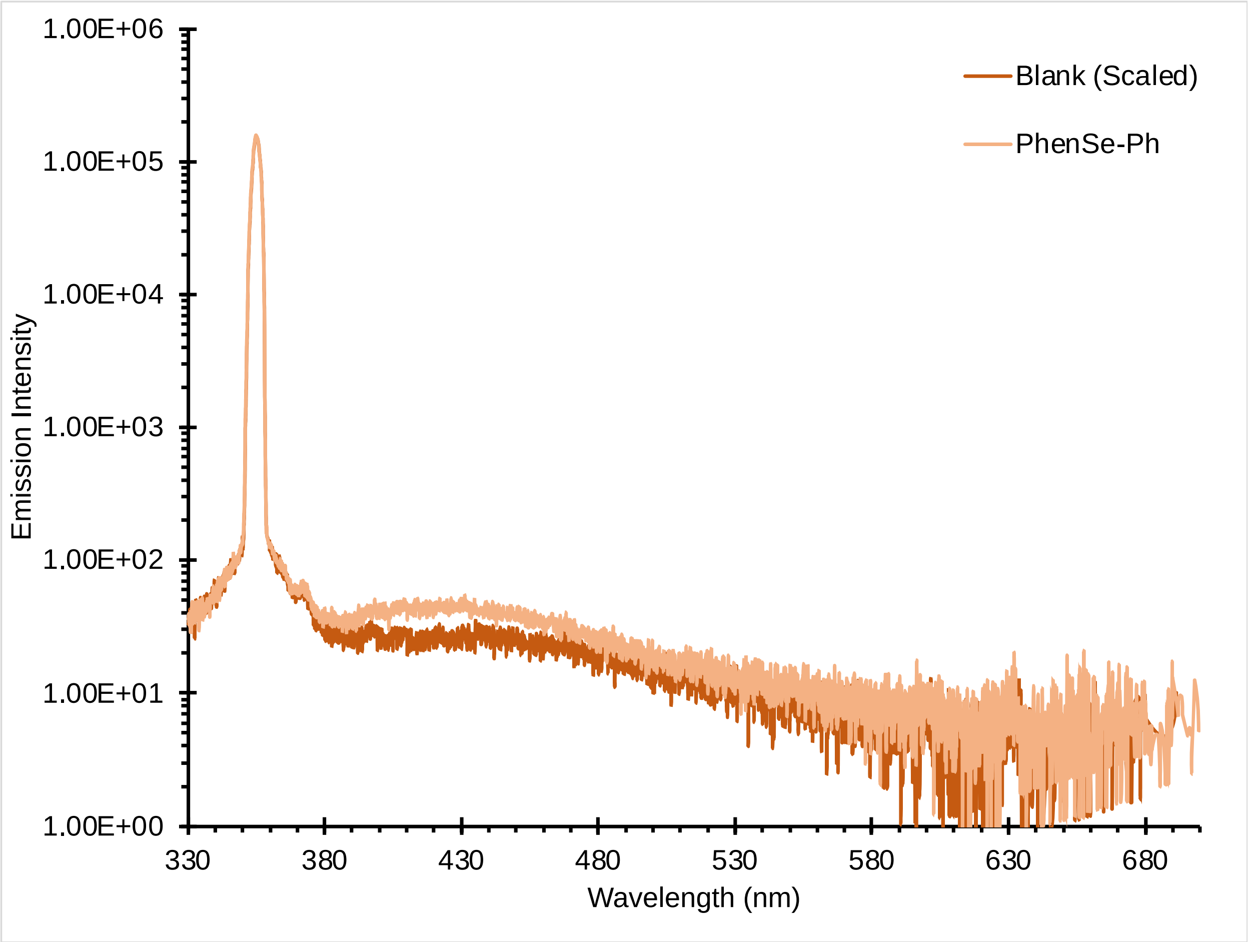

Figure S42. Emission spectra of **4** used for the measurement of fluorescence quantum yield. Sample was excited at 355 nm.

6. Time Correlated Single Photon Counting

Singlet excited state lifetimes for PCs **1 – 4** were measured by TCSPC. In each case, PC solutions were prepared in DMAc in a nitrogen filled glovebox in air-free cuvettes equipped with Kontes valves. Measurements were performed until a maximum count value of 10,000 counts was achieved, and the data were fit with exponential tail fits excluding the instrument response region. Measurements were repeated in triplicate so average lifetimes could be reported, but only one representative set of data per PC is shown below. For details related to each measurements, such as the excitation wavelength, the emission wavelength followed for the measurement, and [PC], please see the figure captions below.

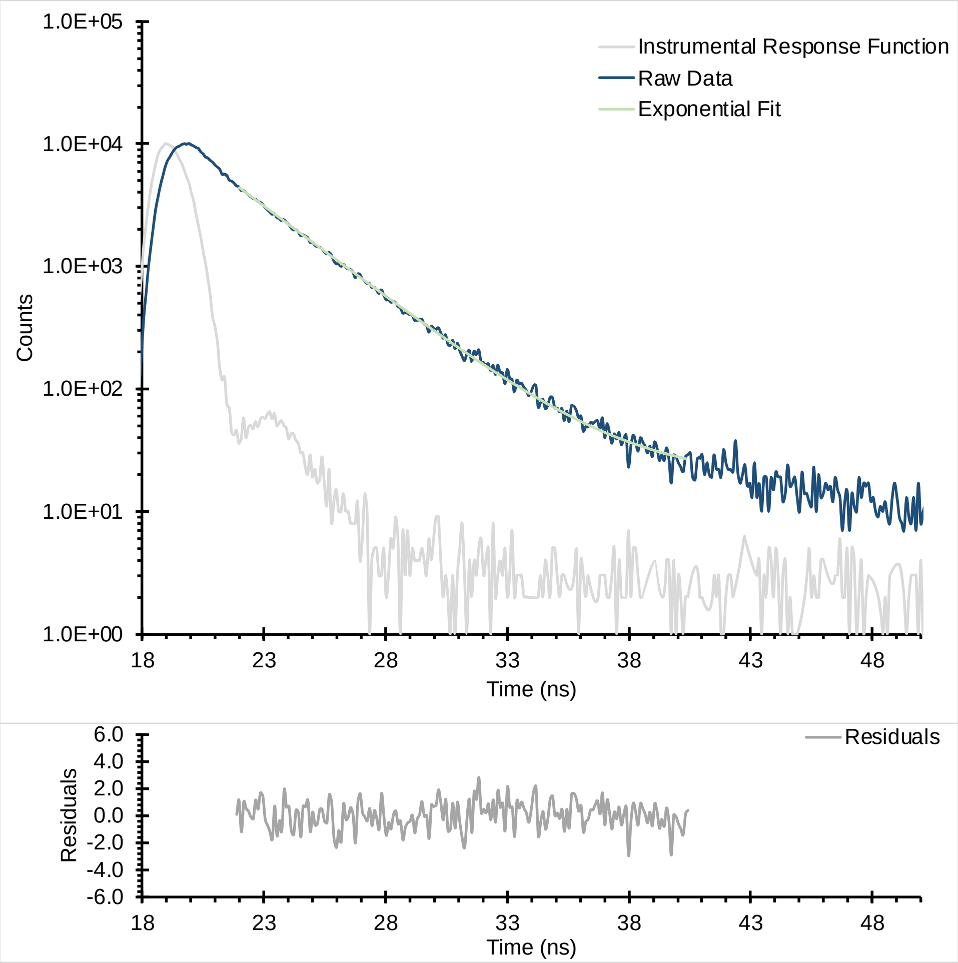

Figure S43. Fluorescence decay curve of **1** (blue), along with the instrument response function (light grey) and exponential fit of the decay data (green). The data on the bottom (dark grey) shows the residuals from the exponential fit of the data. [**1**] = 13 uM, λ_{ex} = 294 nm, λ_{em} = 391 nm, τ_{S1} = 2.92 ns, χ^2 = 1.0867.

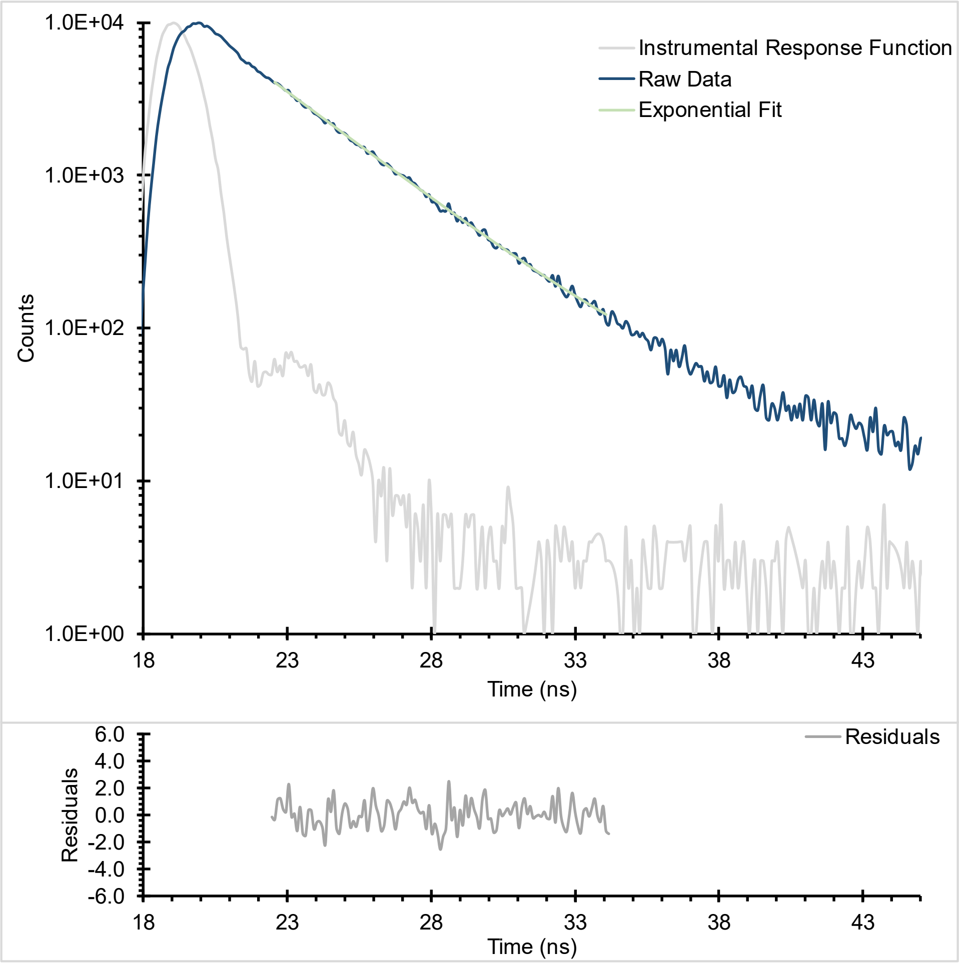

Figure S44. Fluorescence decay curve of **2** (blue), along with the instrument response function (light grey) and exponential fit of the decay data (green). The data on the bottom (dark grey) shows the residuals from the exponential fit of the data. [**2**] = 26 uM, λ_{ex} = 294 nm, λ_{em} = 444 nm, τ_{S1} = 3.07 ns, χ^2 = 1.1995.

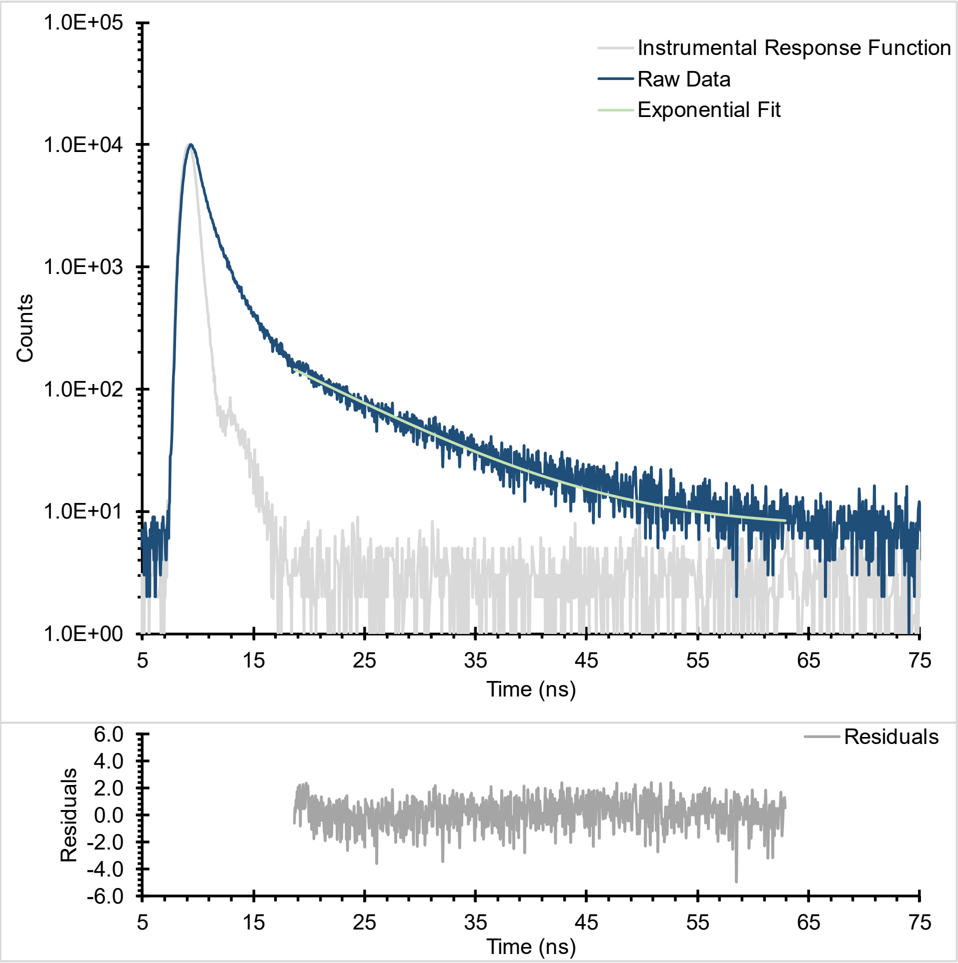

Figure S45. Fluorescence decay curve of **3** (blue), along with the instrument response function (light grey) and exponential fit of the decay data (green). The data on the bottom (dark grey) shows the residuals from the exponential fit of the data. [**3**] = 130 uM, λ_{ex} = 364 nm, λ_{em} = 400 nm, τ_{S1} = 9.19 ns, χ^2 = 1.1463.

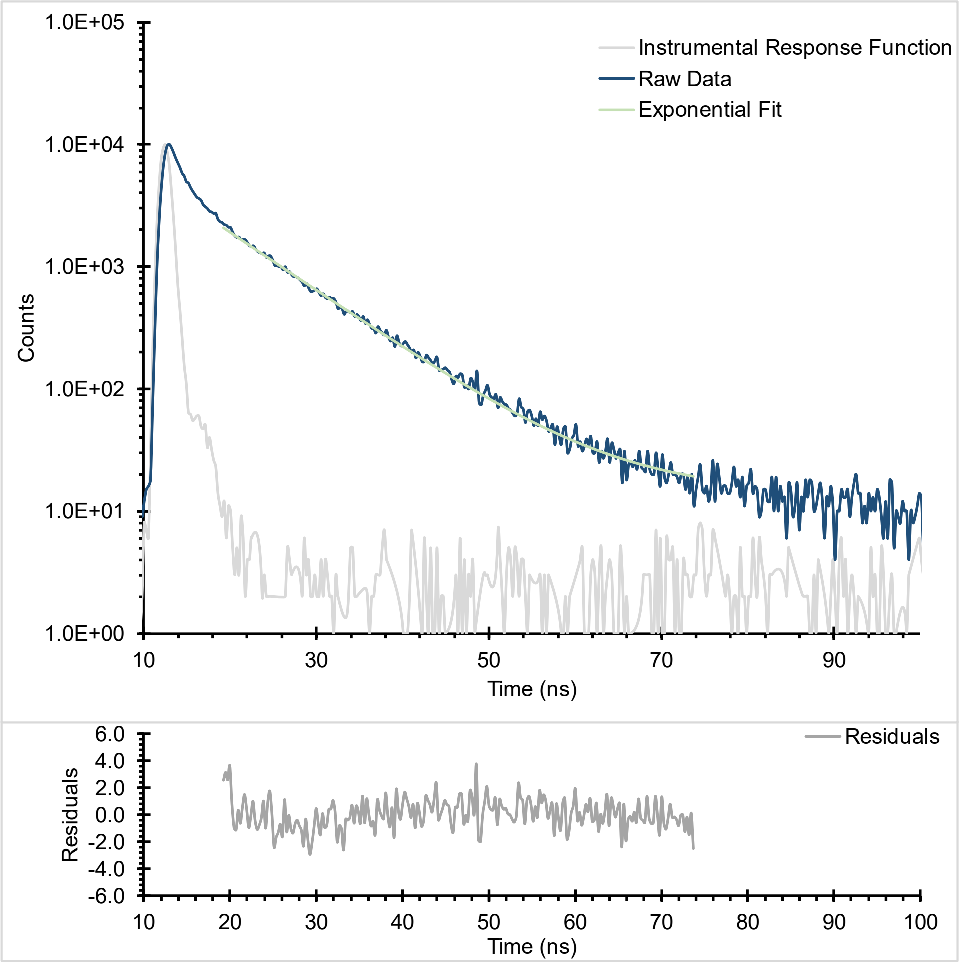

Figure S46. Fluorescence decay curve of **4** (blue), along with the instrument response function (light grey) and exponential fit of the decay data (green). The data on the bottom (dark grey) shows the residuals from the exponential fit of the data. [**4**] = 65 uM, λ_{ex} = 364 nm, λ_{em} = 418 nm, τ_{S1} = 9.03 ns, χ^2 = 1.2949.

Table S5. TCSPC results for PCs **1 – 4**.

**PC**	$\tau_{singlet}$ **(ns)**	χ^2	**Average τ (ns)**
	2.92	1.0867	
**1**	2.95	1.1767	2.95 ± 0.03
	2.98	1.0739	
	3.07	1.1995	
**2**	3.17	1.1361	3.15 ± 0.08
	3.22	1.1475	
	9.19	1.1463	
**3**	9.34	1.1583	9.10 ± 0.30
	8.77	1.0035	
	9.57	1.0162	
**4**	9.03	1.2949	9.33 ± 0.27
	9.39	1.2770	

7. Electrochemical Characterization

Samples were characterized by cyclic voltammetry in two solvents: DMAc and DCM. In each case, the supporting electrolyte was 0.1 M tetra-*n*-butylammonium hexafluorophosphate (Bu_4NPF_6), the working electrode was a glassy carbon disk, the counter electrode was a Pt disk, and the reference electrode was Ag/$AgNO_3$ (0.01 M in acetonitrile with 0.1 M Bu_4NPF_6 supporting electrolyte). For experiments at a scan rate of 10,000 mV s^{-1}, a 100 µm Pt disk micro-electrode was used as the working electrode. Potentials were converted versus the saturated calomel electrode (SCE) by adding 0.29 V to the potentials versus Ag/$AgNO_3$. Prior to measurement, the solutions were sparged with N_2 for 10-15 minutes, and measurements were performed under a positive pressure of N_2. In DMAc, both oxidation and reduction scans were performed, but no reduction events were observed.

Cyclic Voltammetry in *N,N*-Dimethylacetamide

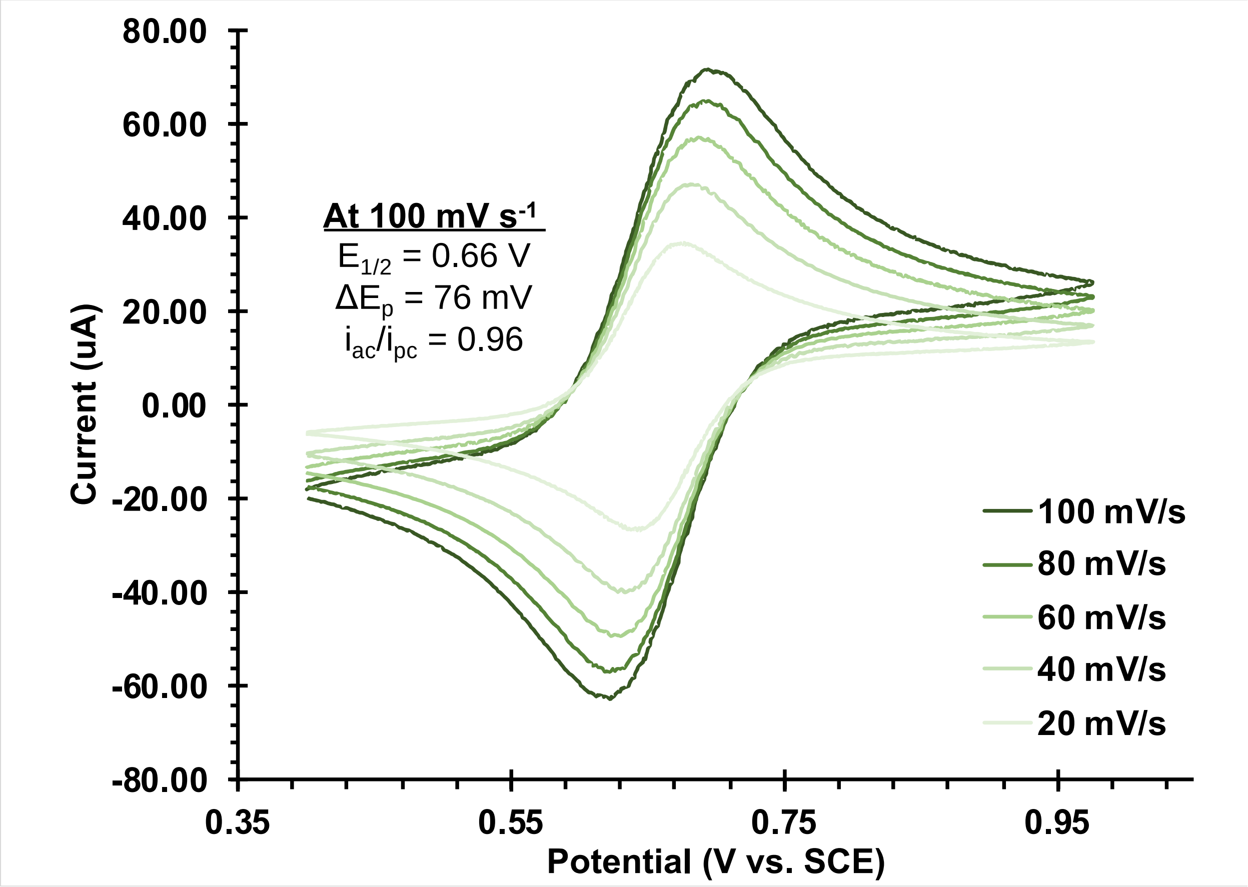

Figure S47. Cyclic voltammograms of **1** in DMAc at various scan rates.

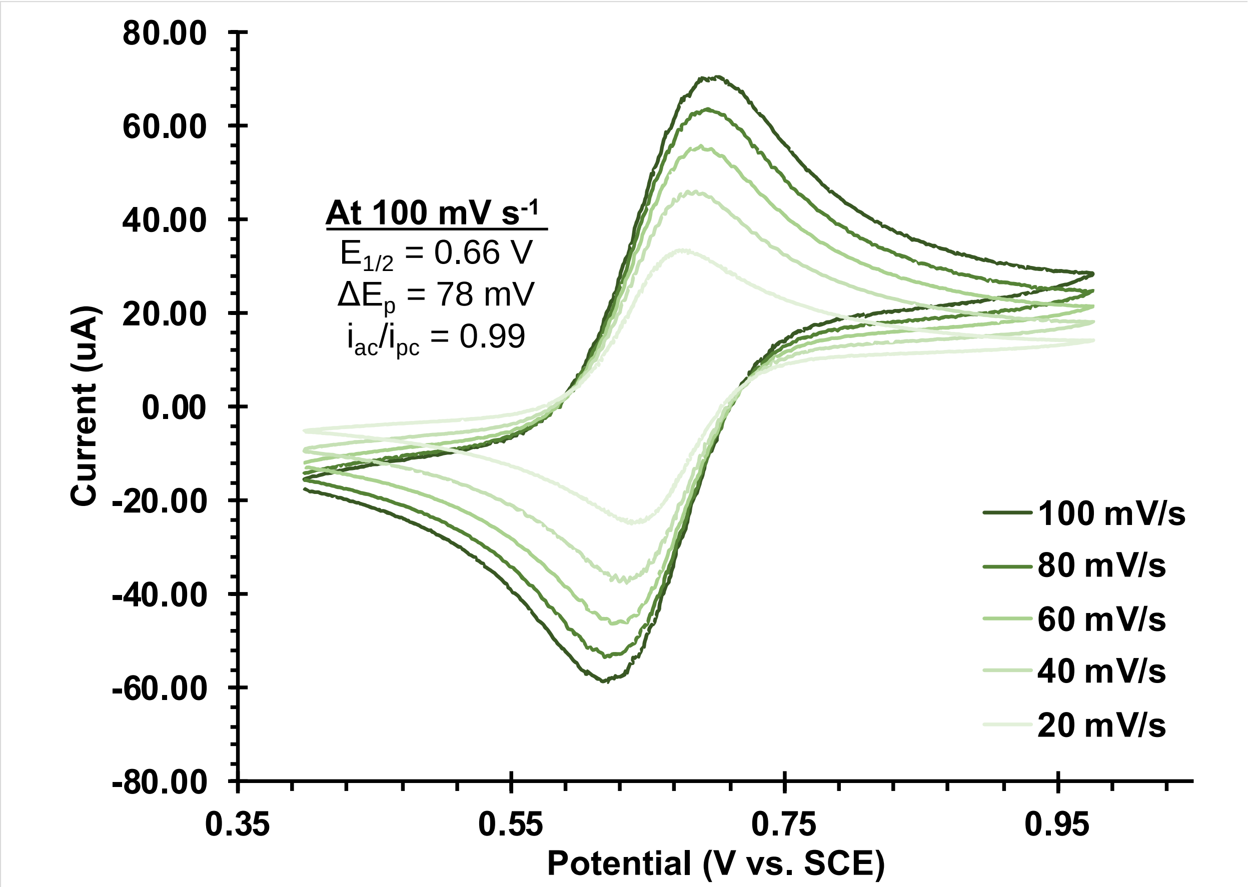

Figure S48. Cyclic voltammograms of **2** in DMAc at various scan rates.

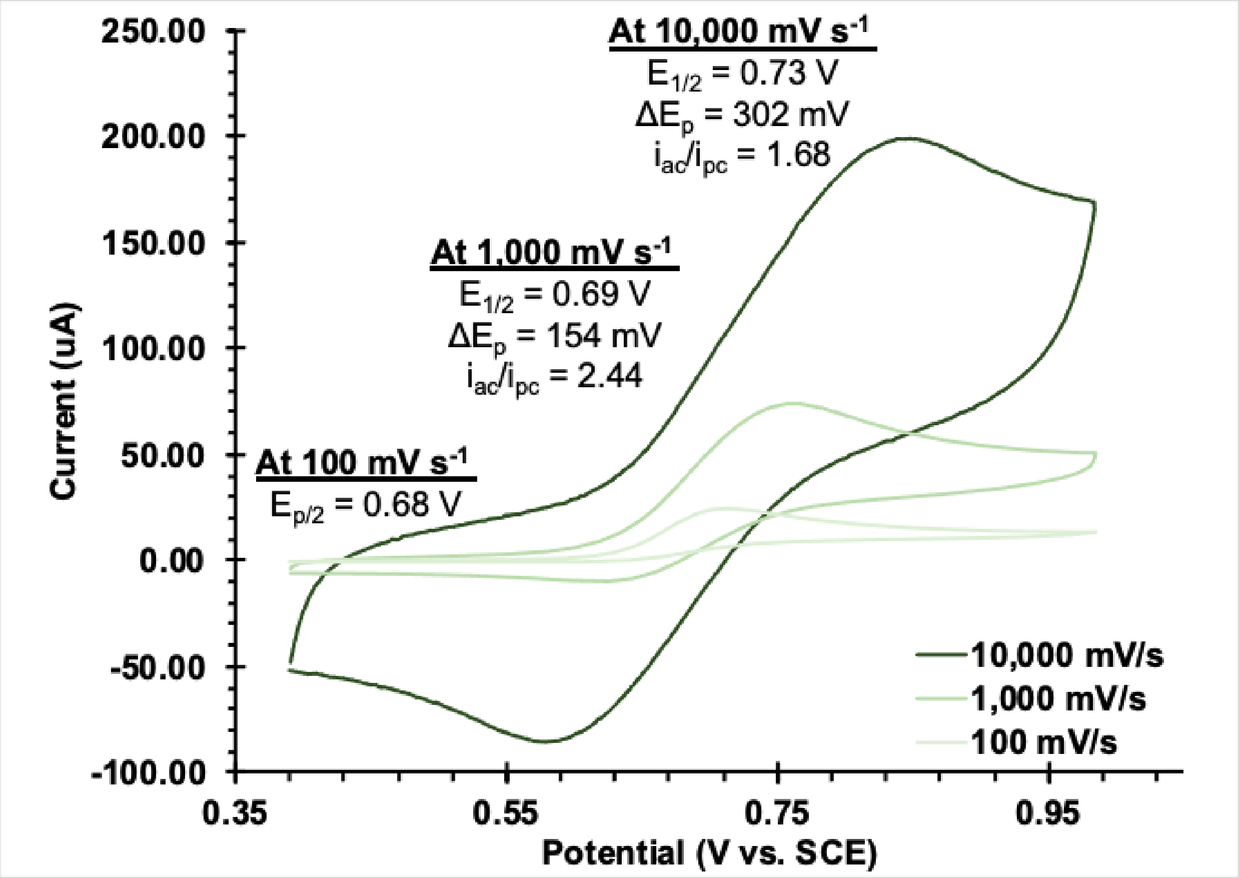

Figure S49. Cyclic voltammograms of **3** in DMAc at various scan rates.

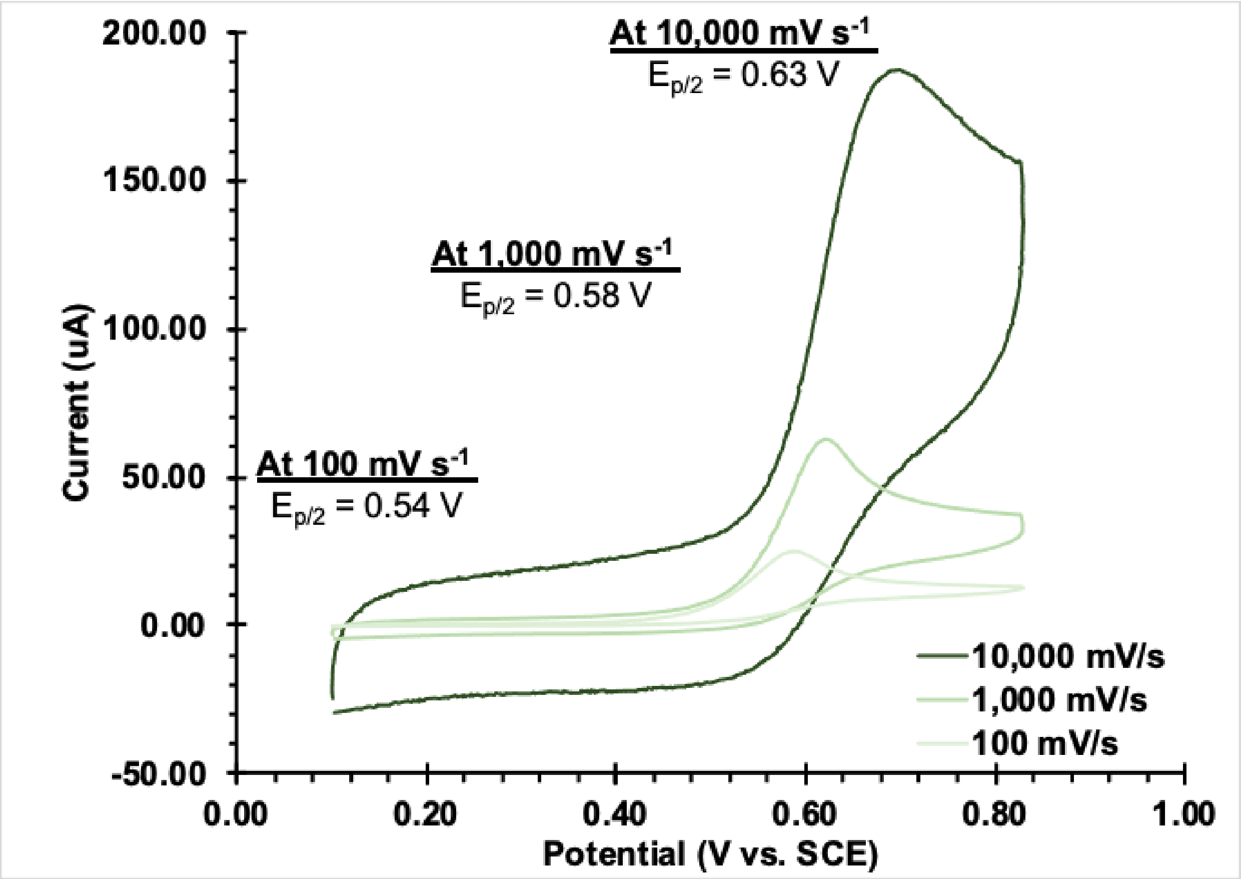

Figure S50. Cyclic voltammograms of **4** in DMAc at various scan rates.

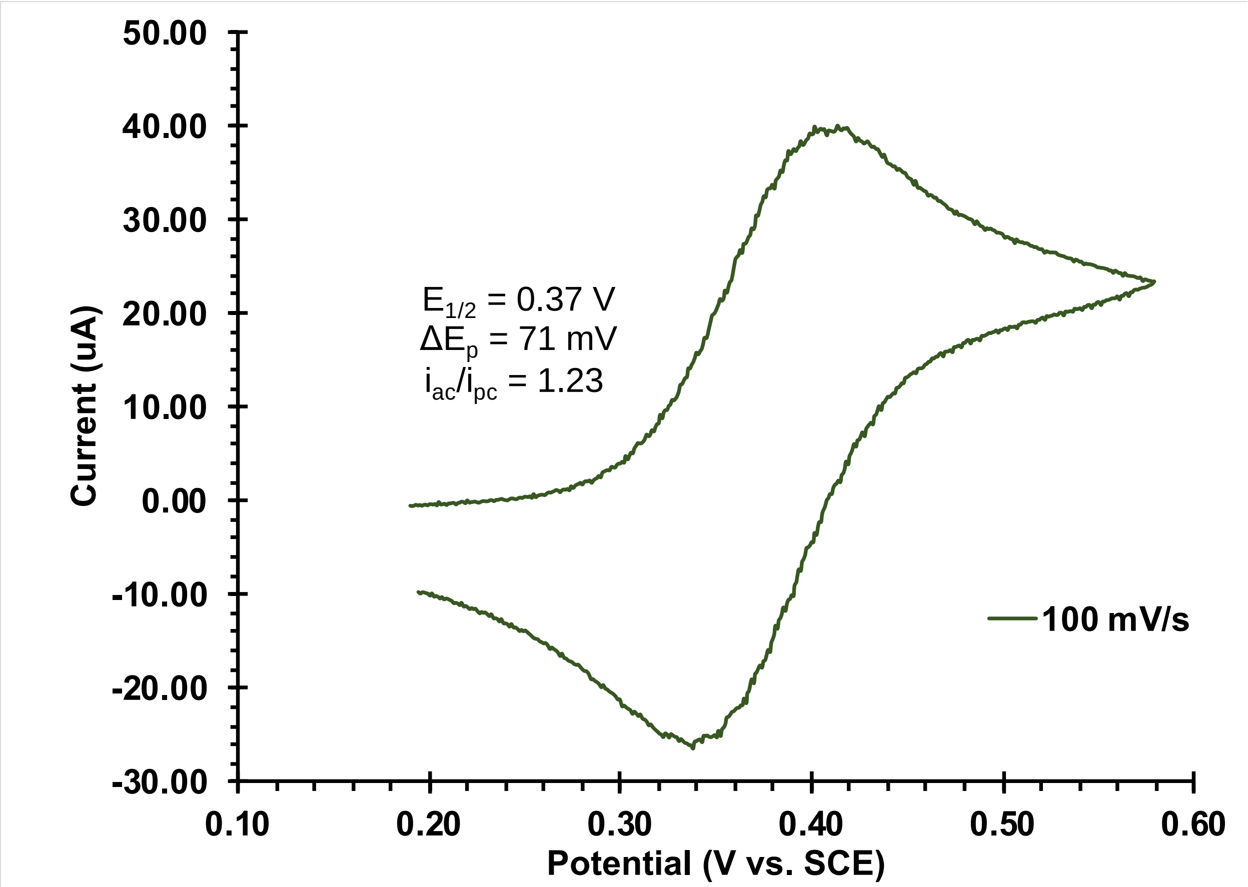

Figure S51. Cyclic voltammogram of ferrocene under the same conditions used to measure PCs **1** – **4** in DMAc.

Cyclic Voltammetry in Dichloromethane

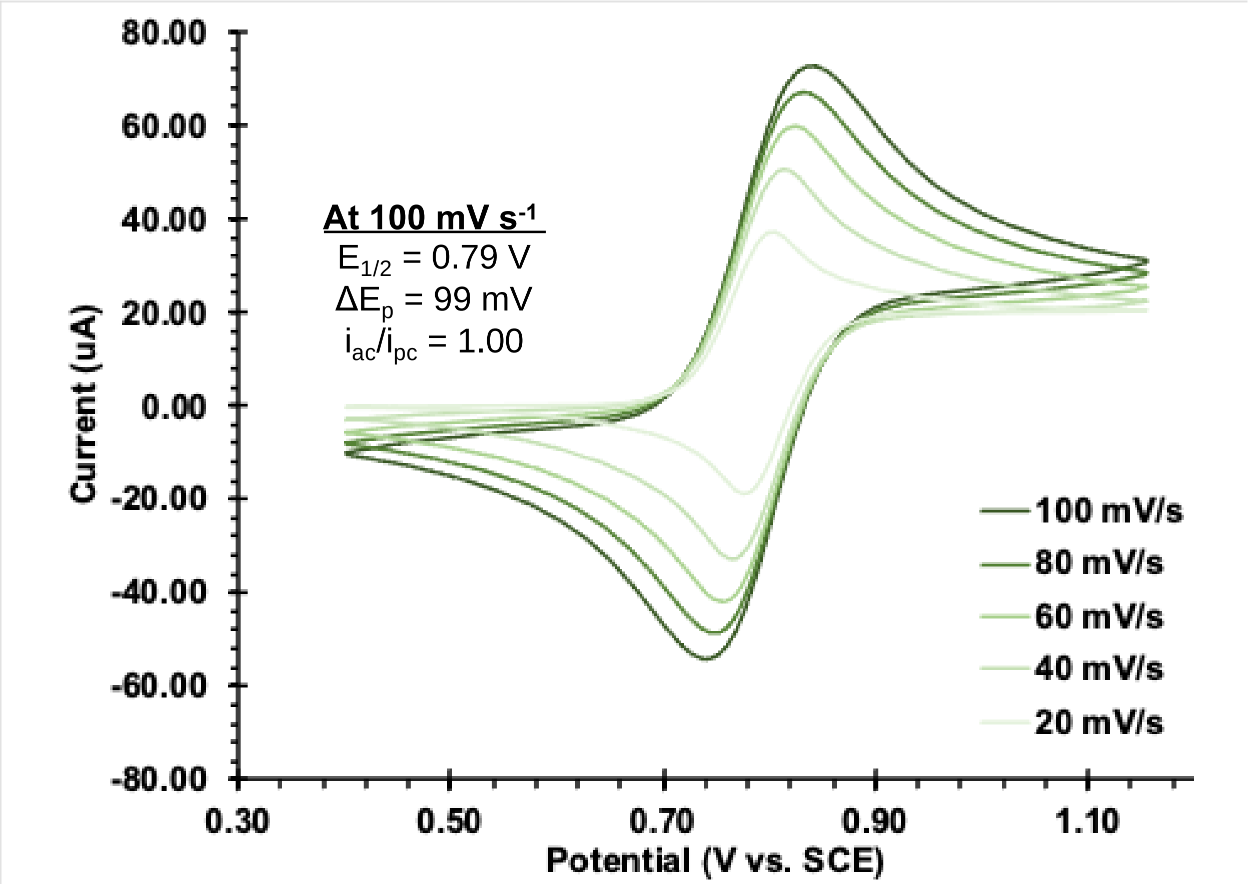

Figure S52. Cyclic voltammograms of **1** in DCM at various scan rates.

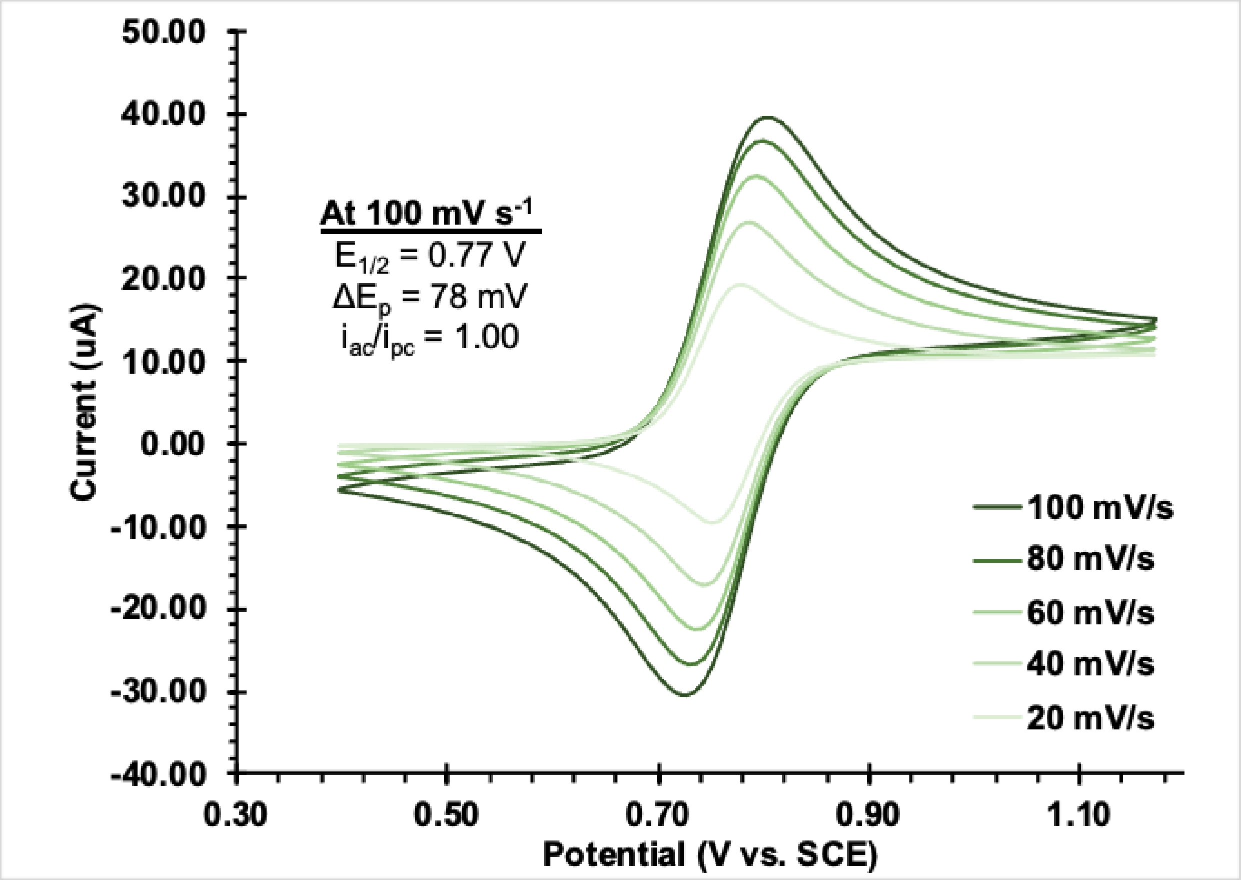

Figure S53. Cyclic voltammograms of **2** in DCM at various scan rates.

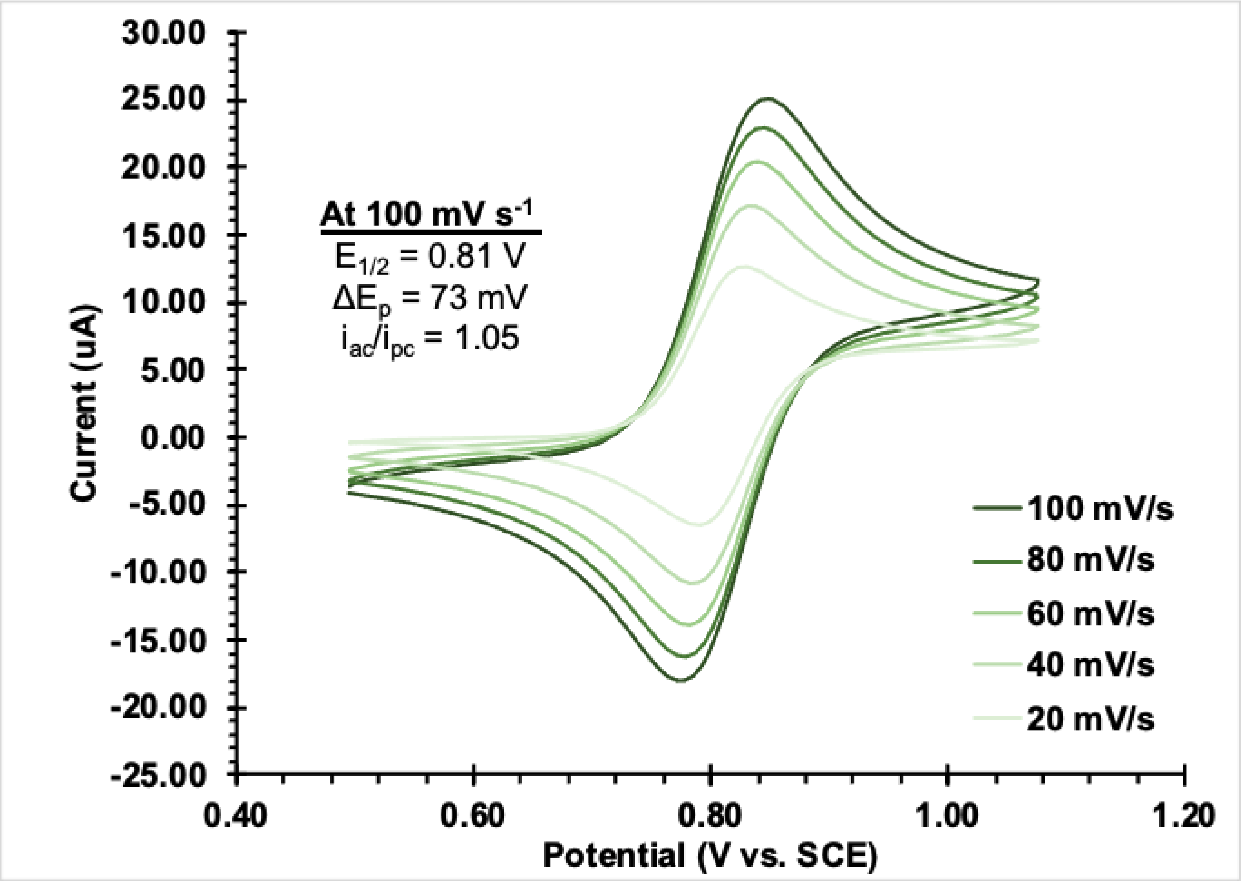

Figure S54. Cyclic voltammograms of **3** in DCM at various scan rates.

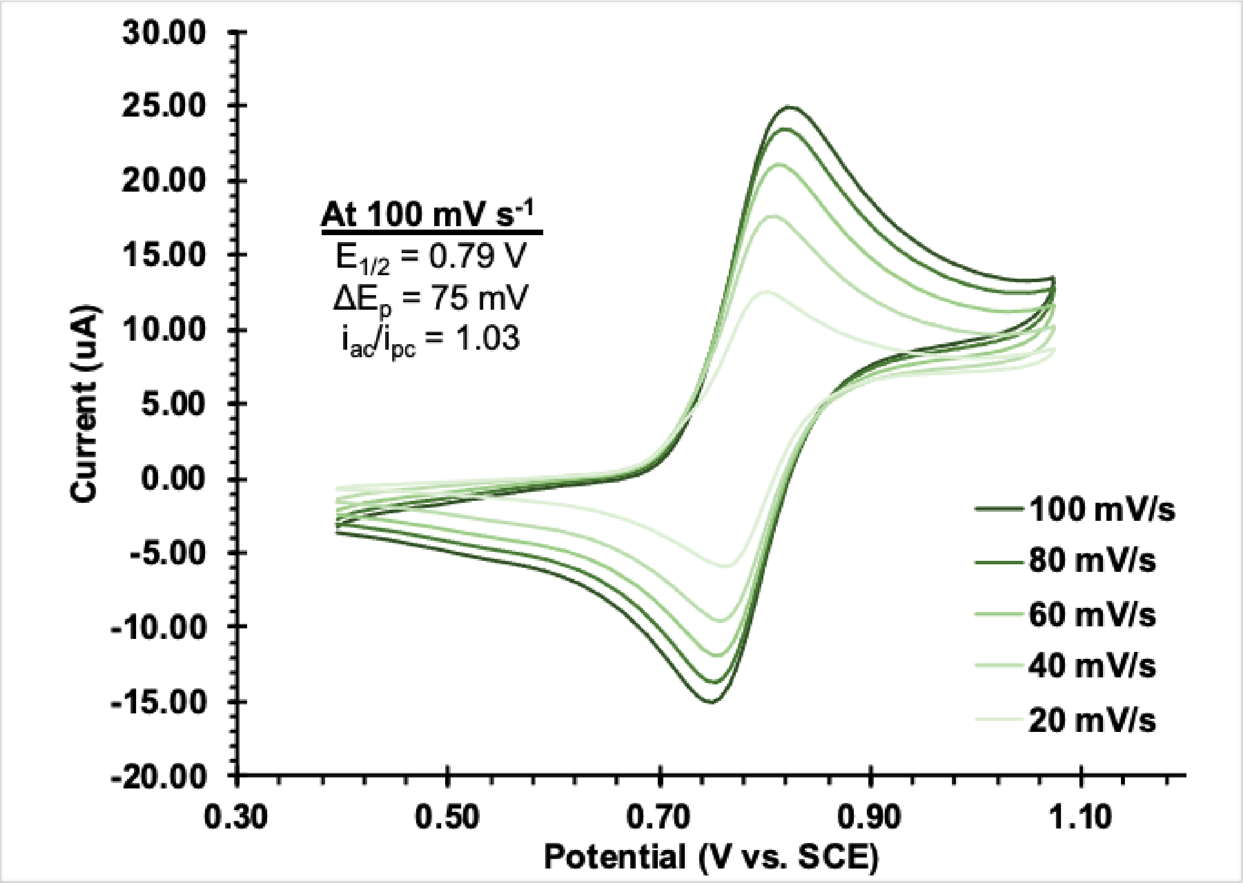

Figure S55. Cyclic voltammograms of **4** in DCM at various scan rates.

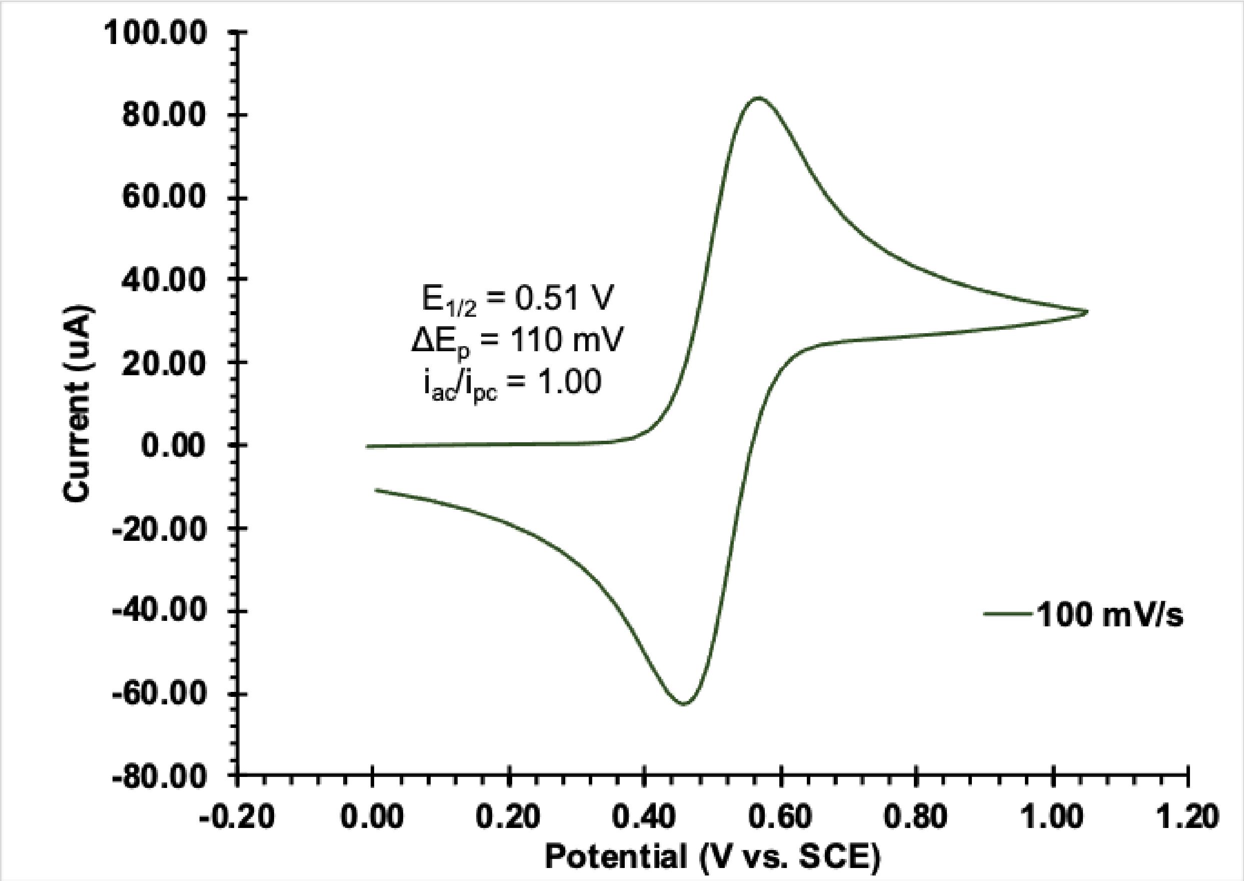

Figure S56. Cyclic voltammogram of ferrocene under the same conditions used to measure PCs **1 – 4** in DCM.

Estimation of $E_{1/2}$ in *N,N*-Dimethylacetamide From Data in Dichloromethane

Since the reversibility of PCs **3** and **4** was much better in DCM than in DMAc, this data was used to estimate the $E_{1/2}$ of PCs **3** and **4** in DMAc. The ferrocene/ferrocenium (Fc/Fc^+) redox couple is often used as a standard in organic electrochemistry, and many have recommended referencing electrochemical data to this system as a means of standardizing electrochemical data that is obtained under a wide range of conditions.[7,8] As such, it was reasoned the Fc/Fc^+ redox couple could be used to convert the $E_{1/2}$ of a PC from DCM to DMAc, since both the Fc/Fc^+ and $PC/PC^{\bullet+}$ redox couples would shift in different solvents, but the distance between these redox couples should remain the same. Indeed, when these values were compared for PCs **1** and **2**, which showed good reversibility in both DMAc and DCM, it was found that their values of $E_{1/2}$ were consistently about 0.28 V above the $E_{1/2}$ of ferrocene, regardless of solvent (Table S6).

Table S6. Oxidation potentials of ferrocene and PCs **1** and **2** in DMAc and DCM.

Compound	Solvent	$E_{1/2}$ (V vs. SCE)	$E_{Ferrocene}$ - E_{PC} (V)
Ferrocene	DMAc	0.37	-
	DCM	0.51	-
**1**	DMAc	0.66	0.29
	DCM	0.79	0.28
**2**	DMAc	0.66	0.29
	DCM	0.77	0.26

This property can be summarized mathematically as Equation S2:

$$E_{1/2}(\text{PC vs. Fc/Fc}^+) = E_{1/2}(\text{PC in DCM}) - E_{1/2}(\text{Fc in DCM}) = E_{1/2}(\text{PC in DMAc}) - E_{1/2}(\text{Fc in DMAc}) \quad \text{Eq. (S2)}$$

Rearranging this equation, we get Equation S3, which allows for the conversion of $E_{1/2}$ between solvents based on the shift in the ferrocene redox couple:

$$E_{1/2}(\text{PC in DMAc}) = E_{1/2}(\text{PC in DCM}) - E_{1/2}(\text{Fc in DCM}) + E_{1/2}(\text{Fc in DMAc}) \quad \text{Eq. (S3)}$$

Since the $E_{1/2}$ of PCs **1** and **2** could be measured reliably in DMAc, these values are reported in Table 2 of this manuscript. Instead, the method described above was used to estimate the $E_{1/2}$ of PCs **3** and **4** in DMAc, and these estimated values are reported in Table 2 of this manuscript.

8. Supplemental Polymerization Data

Procedure for the Analysis of Kinetics and Molecular Weight Growth

To monitor polymerizations, 0.1 mL aliquots were removed periodically using a nitrogen purged syringe and needle. Aliquots were quenched in a deuterated chloroform containing 250 ppm butylated hydroxytoluene (BHT). These solutions were then transferred to an NMR tube for ^{1}H NMR analysis to determine the extent of monomer conversion. Afterwards, solutions were dried and dissolved in unstabilized THF for GPC analysis to obtain number average molecular weight and dispersity.

Supplemental Data

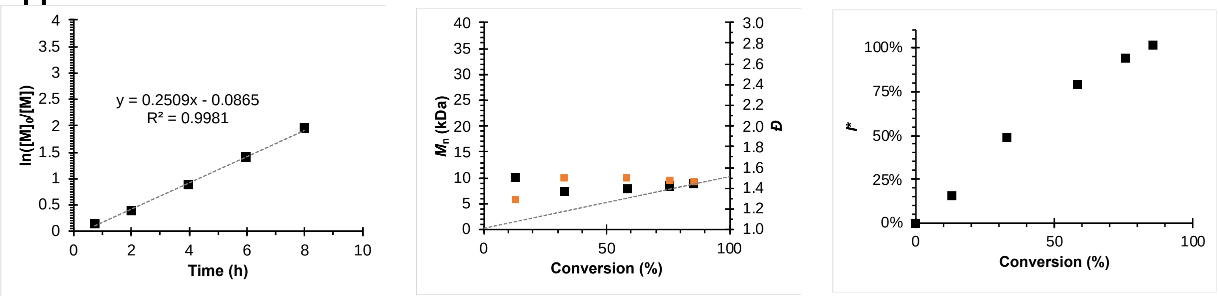

Figure S57. O-ATRP of MMA using **1**. [MMA]:[DBMM]:[**1**] = [1000]:[10]:[1]; 1 mL MMA, 1 mL DMAc; irradiated in a 365 nm LED beaker. (Left) Pseudo-first order kinetics plot. (Middle) Evolution of polymer molecular weight (black) and *Đ* (orange) as a function of monomer conversion; grey dashed line represents the theoretical molecular weight growth. (Right) Initiator efficiency as a function of monomer conversion.

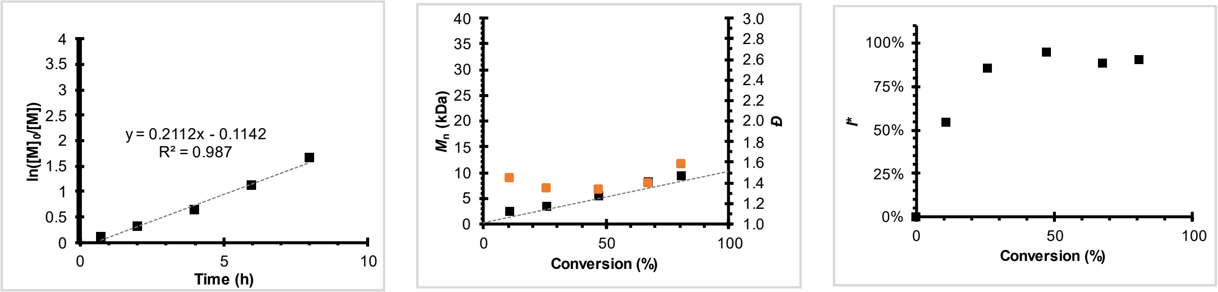

Figure S58. O-ATRP of MMA using **2**. [MMA]:[DBMM]:[**2**] = [1000]:[10]:[1]; 1 mL MMA, 1 mL DMAc; irradiated in a 365 nm LED beaker. (Left) Pseudo-first order kinetics plot. (Middle) Evolution of polymer molecular weight (black) and *Đ* (orange) as a function of monomer conversion; grey dashed line represents the theoretical molecular weight growth. (Right) Initiator efficiency as a function of monomer conversion.

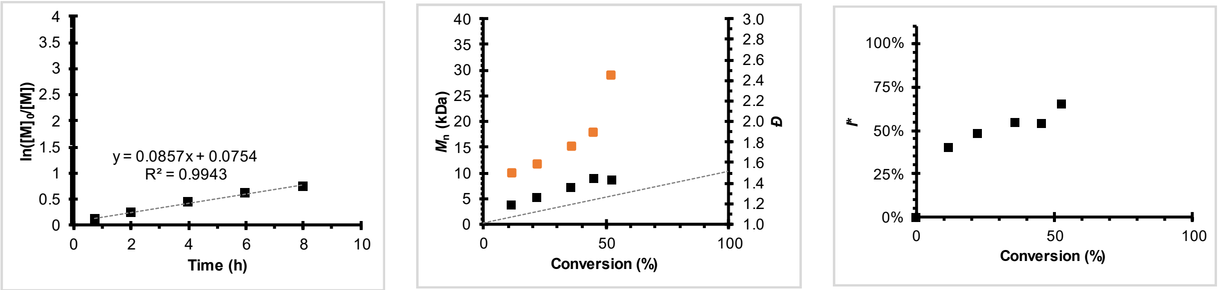

Figure S59. O-ATRP of MMA using **3**. [MMA]:[DBMM]:[**3**] = [1000]:[10]:[1]; 1 mL MMA, 1 mL DMAc; irradiated in a 365 nm LED beaker. (Left) Pseudo-first order kinetics plot. (Middle)

Evolution of polymer molecular weight (black) and *Đ* (orange) as a function of monomer conversion; grey dashed line represents the theoretical molecular weight growth. (Right) Initiator efficiency as a function of monomer conversion.

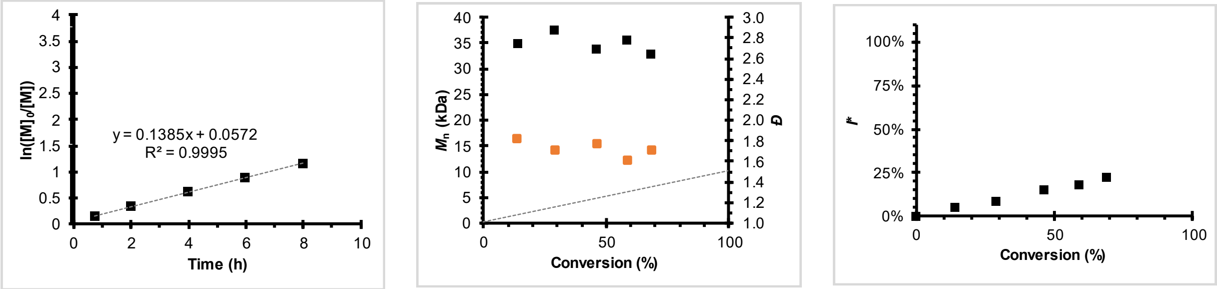

Figure S60. O-ATRP of MMA using **4**. [MMA]:[DBMM]:[**4**] = [1000]:[10]:[1]; 1 mL MMA, 1 mL DMAc; irradiated in a 365 nm LED beaker. (Left) Pseudo-first order kinetics plot. (Middle) Evolution of polymer molecular weight (black) and *Đ* (orange) as a function of monomer conversion; grey dashed line represents the theoretical molecular weight growth. (Right) Initiator efficiency as a function of monomer conversion.

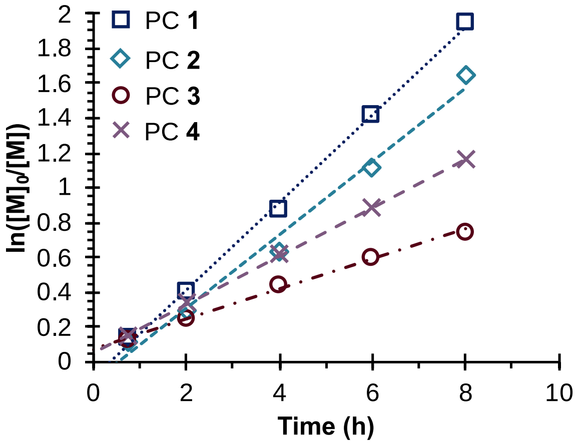

Figure S61. Overlaid polymerization kinetics for the O-ATRP of MMA using PCs **1** – **4**.

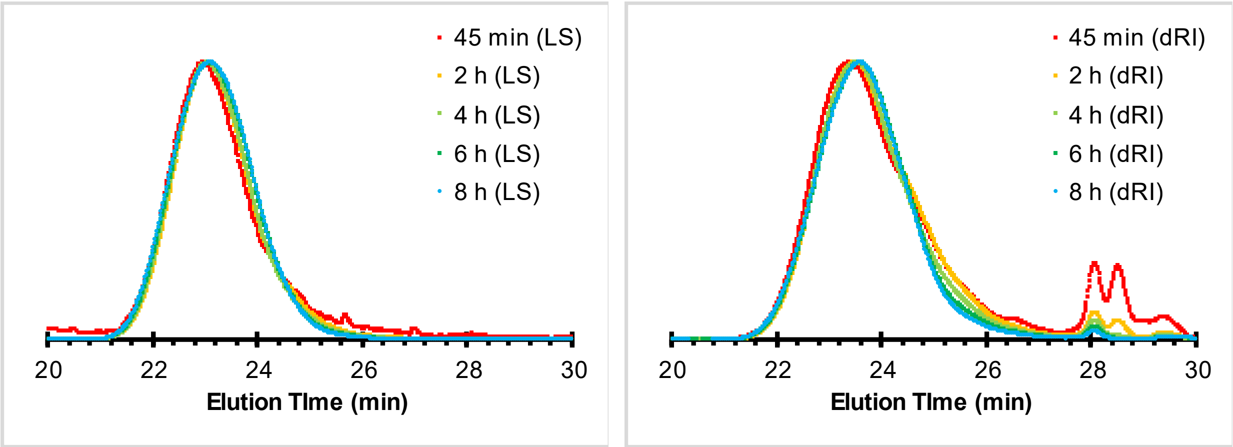

Figure S62. GPC traces for O-ATRP of MMA with **1**. [MMA]:[DBMM]:[**1**] = [1000]:[10]:[1]; 1 mL MMA, 1 mL DMAc; irradiated in a 365 nm LED beaker. Detectors: multi-angle light scattering (left) and differential refractive index (right).

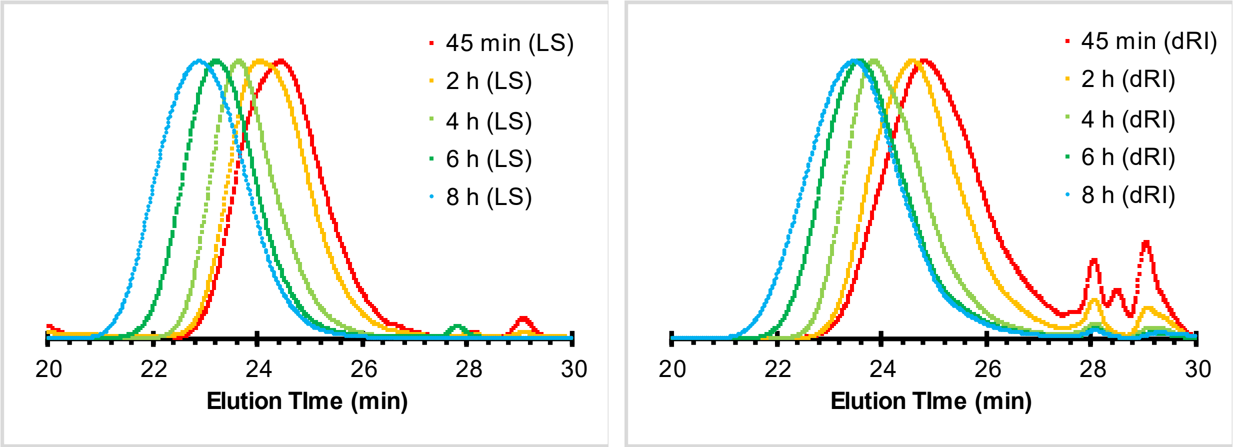

Figure S63. GPC traces for O-ATRP of MMA with **2**. [MMA]:[DBMM]:[**2**] = [1000]:[10]:[1]; 1 mL MMA, 1 mL DMAc; irradiated in a 365 nm LED beaker. Detectors: multi-angle light scattering (left) and differential refractive index (right).

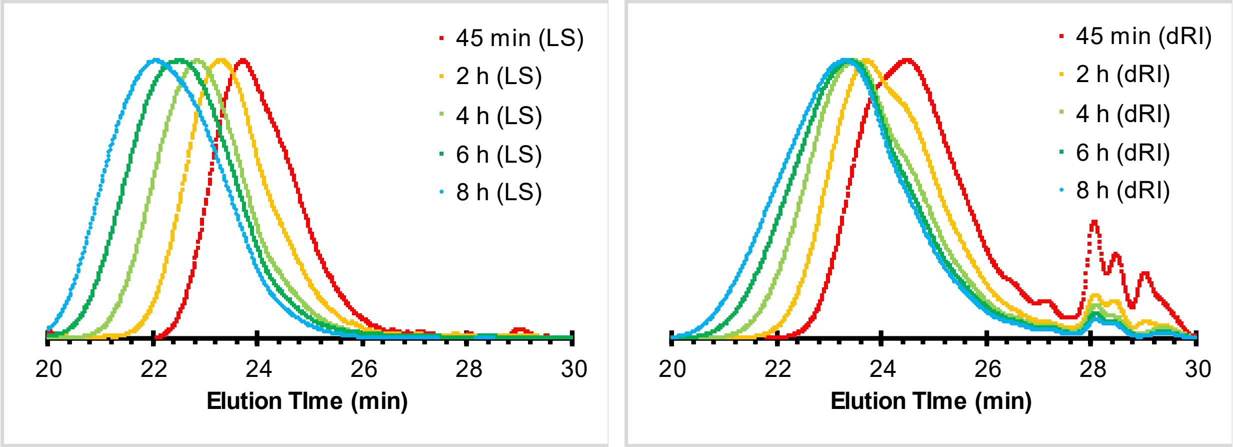

Figure S64. GPC traces for O-ATRP of MMA with **3**. [MMA]:[DBMM]:[**3**] = [1000]:[10]:[1]; 1 mL MMA, 1 mL DMAc; irradiated in a 365 nm LED beaker. Detectors: multi-angle light scattering (left) and differential refractive index (right).

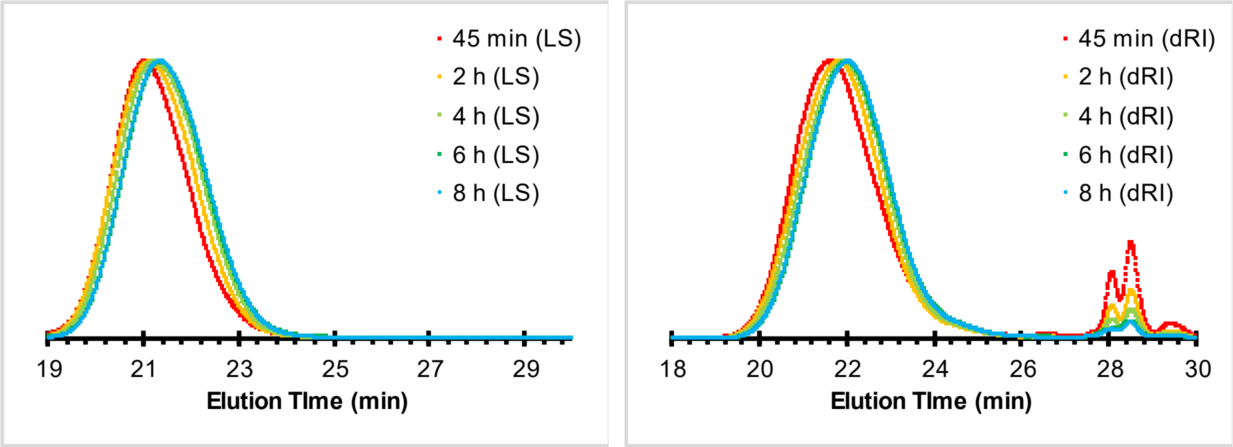

Figure S65. GPC traces for O-ATRP of MMA with **4**. [MMA]:[DBMM]:[**4**] = [1000]:[10]:[1]; 1 mL MMA, 1 mL DMAc; irradiated in a 365 nm LED beaker. Detectors: multi-angle light scattering (left) and differential refractive index (right).

Table S7. Single time-point data at the end of polymerizations with PCs **1 – 4**, first run.

PC	Time (h)	Conv. (%)	$M_{n,theo}$ (kDa)	$M_{n,exp}$ (kDa)[a]	$Đ^a$	I^* (%)[b]
**1**	8	86	8.83	8.72	1.46	101
**2**	8	81	8.34	9.26	1.58	90
**3**	8	52	5.50	8.48	2.45	65
**4**	8	69	7.13	32.7	1.70	22

[a] Determined by GPC. [b] Initiator efficiency (I^*) = ($M_{n,\ theo}$ / $M_{n,\ exp}$)•100%.

Table S8. Single time-point data at the end of polymerizations with PCs **1 – 4**, second run.

PC	Time (h)	Conv. (%)	$M_{n,theo}$ (kDa)	$M_{n,exp}$ (kDa)[a]	$Đ^a$	I^* (%)[b]
**1**	8	80	8.25	8.44	1.53	98
**2**	8	75	7.77	8.97	1.44	87
**3**	8	48	5.02	8.47	1.92	59
**4**	8	64	6.68	37.4	1.66	18

[a] Determined by GPC. [b] Initiator efficiency (I^*) = ($M_{n,\ theo}$ / $M_{n,\ exp}$)•100%.

Table S9. Single time-point data at the end of polymerizations with PCs **1 – 4**, third run.

PC	Time (h)	Conv. (%)	$M_{n,theo}$ (kDa)	$M_{n,exp}$ (kDa)[a]	$Đ^a$	I^* (%)[b]
**1**	8	77	7.91	9.03	1.51	88
**2**	8	66	6.86	7.38	1.39	93
**3**	8	46	4.88	7.67	2.00	64
**4**	8	58	6.02	42.4	1.67	14

[a] Determined by GPC. [b] Initiator efficiency (I^*) = ($M_{n,\ theo}$ / $M_{n,\ exp}$)•100%.

Table S10. Averaged single time-point polymerization data for PCs **1 – 4**.

PC	Time (h)	Conv. (%)	$M_{n,avg}$ (kDa)[a]	$Đ_{avg}{}^a$	I_{avg}^* (%)[b]
**1**	8	81 ± 5	8.73 ± 0.30	1.50 ± 0.04	96 ± 7
**2**	8	74 ± 7	8.54 ±1.01	1.47 ± 0.10	90 ± 3
**3**	8	49 ± 3	8.21 ± 0.46	2.12 ± 0.29	63 ± 3
**4**	8	64 ± 6	37.5 ± 4.85	1.68 ± 0.02	18 ± 4

[a] Determined by GPC. [b] Initiator efficiency (I^*) = ($M_{n,\ theo}$ / $M_{n,\ exp}$)•100%.

Table S11. Single time-point data at the end of polymerizations with PCs **1** – **4** for control experiments. Control experiments were performed using the same polymerization procedure described herein, but without light. No conversion was observed.

PC	Time (h)	Conv. (%)	$M_{n,theo}$ (kDa)	$M_{n,exp}$ (kDa)[a]	$Đ$[a]	I^* (%)[b]
**1**	8	0	-	-	-	-
**2**	8	0	-	-	-	-
**3**	8	0	-	-	-	-
**4**	8	0	-	-	-	-

[a] Determined by GPC. [b] Initiator efficiency (I^*) = ($M_{n,\ theo}$ / $M_{n,\ exp}$)•100%.

9. Supplemental Photooxidation Data

Procedure for Reaction Analysis

Once reactions were complete, they were removed from the photoreactor and dried under air. The crude product was redissolved in deuterated chloroform (0.4 mL) containing dibromomethane (3.5 uL) as an internal standard. The solution was transferred to an NMR tube and analyzed by ^{1}H NMR to determine product yield.

Reaction NMR Spectra

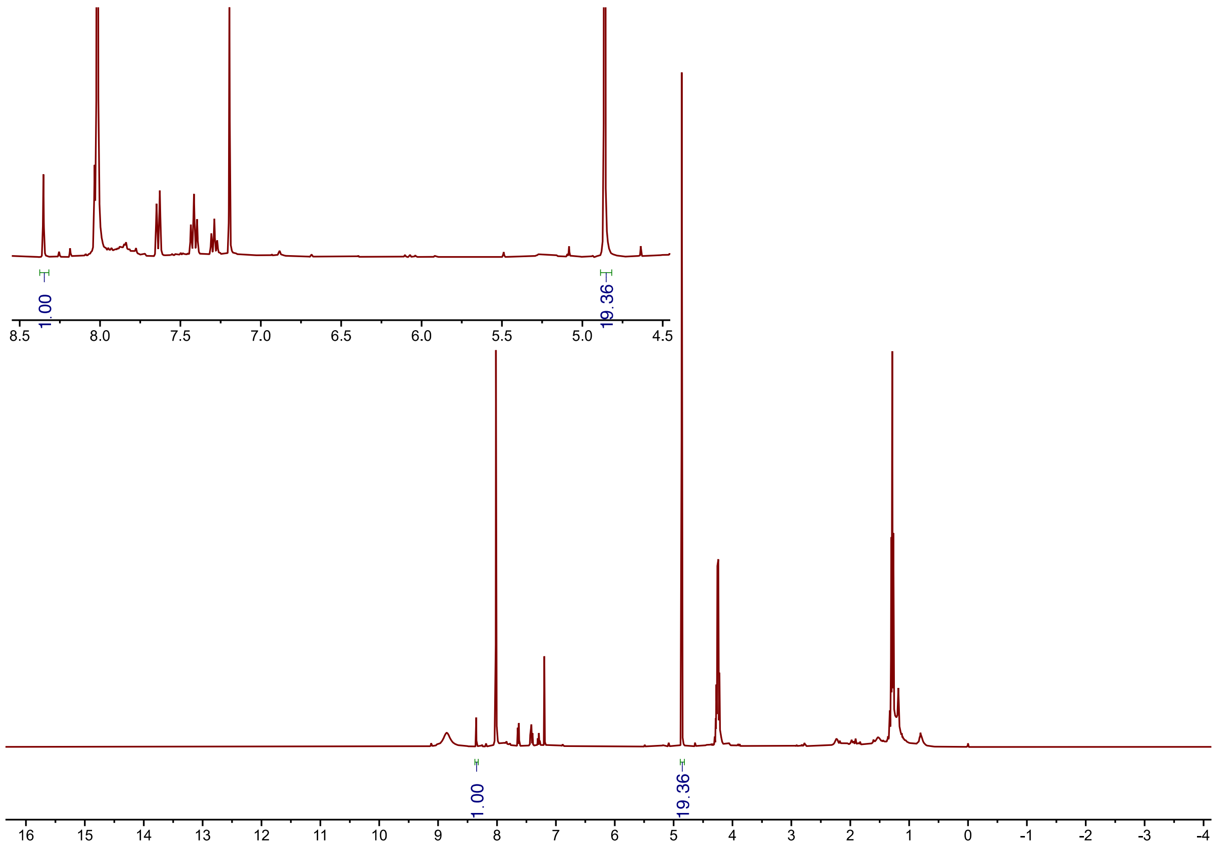

Figure S66. ^{1}H NMR spectrum of the photooxidation reaction mediated by PC **1** (yield = 10%).

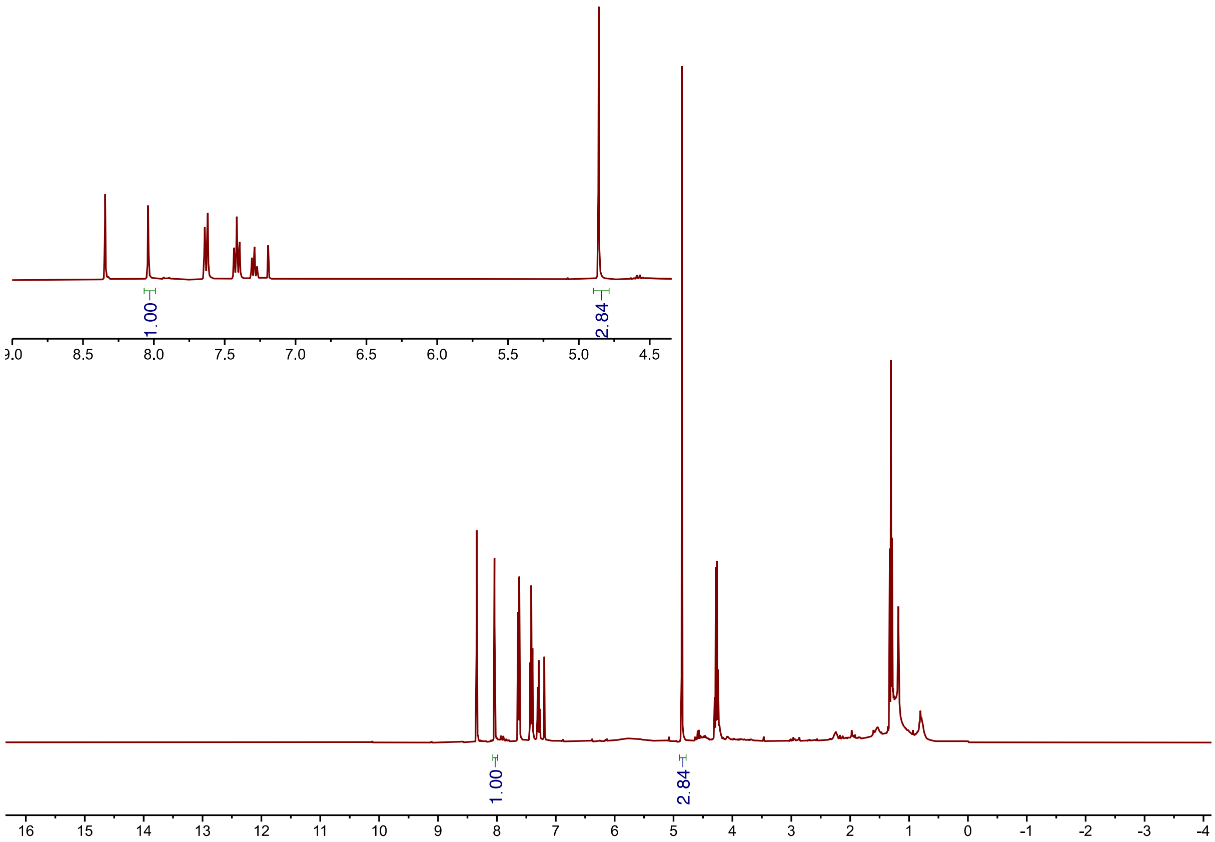

Figure S67. ^{1}H NMR spectrum of the photooxidation reaction mediated by PC **2** run simultaneously with that performed using **1** (yield = 69%).

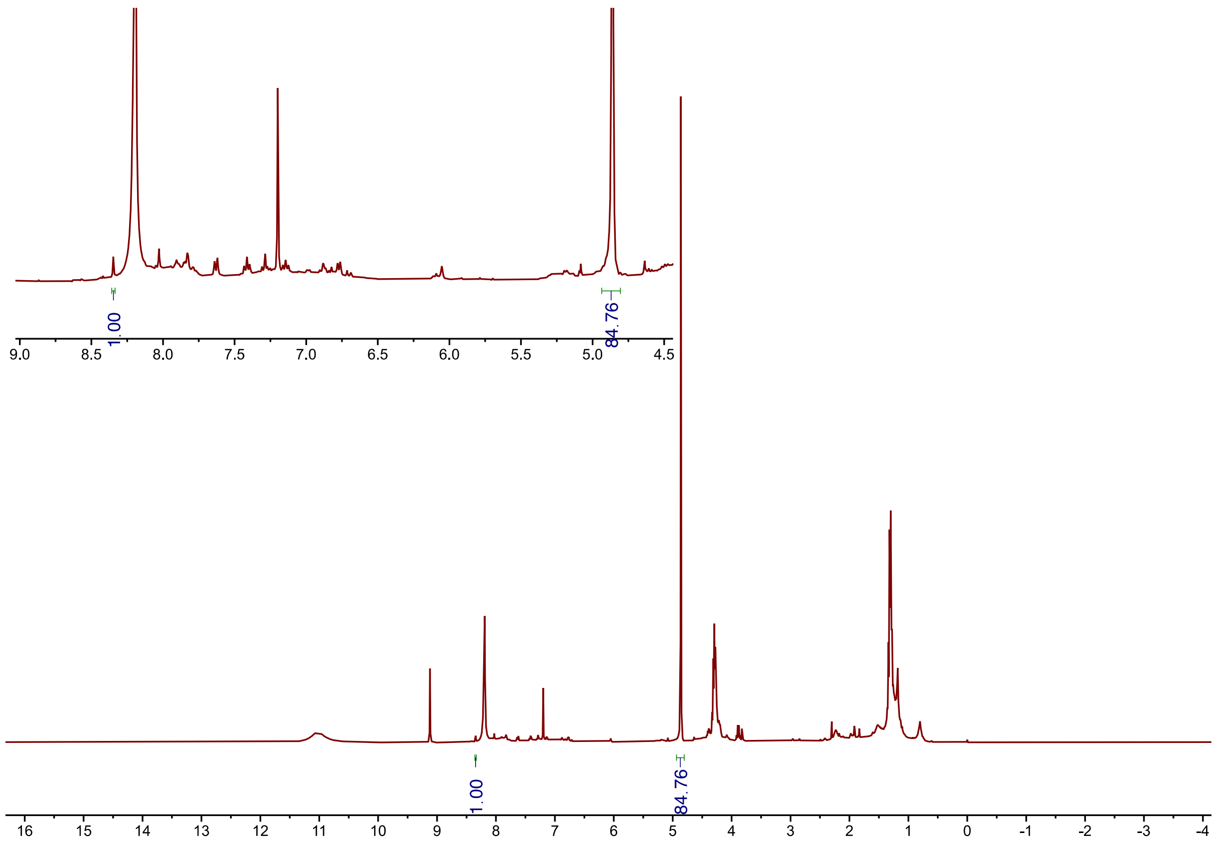

Figure S68. ^{1}H NMR spectrum of the photooxidation reaction mediated by PC **3** (yield = 2%).

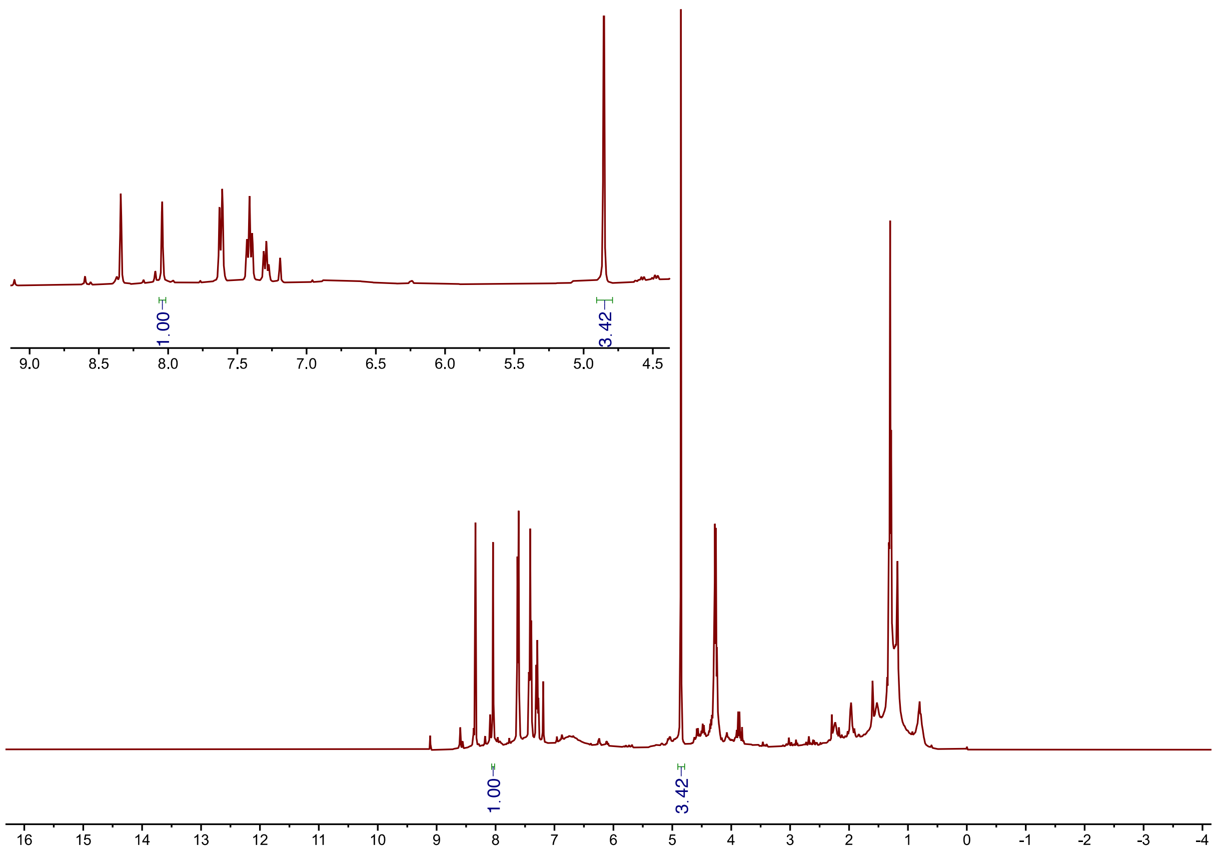

Figure S69. ^{1}H NMR spectrum of the photooxidation reaction mediated by PC **2** run simultaneously with that performed using **3** (yield = 57%).

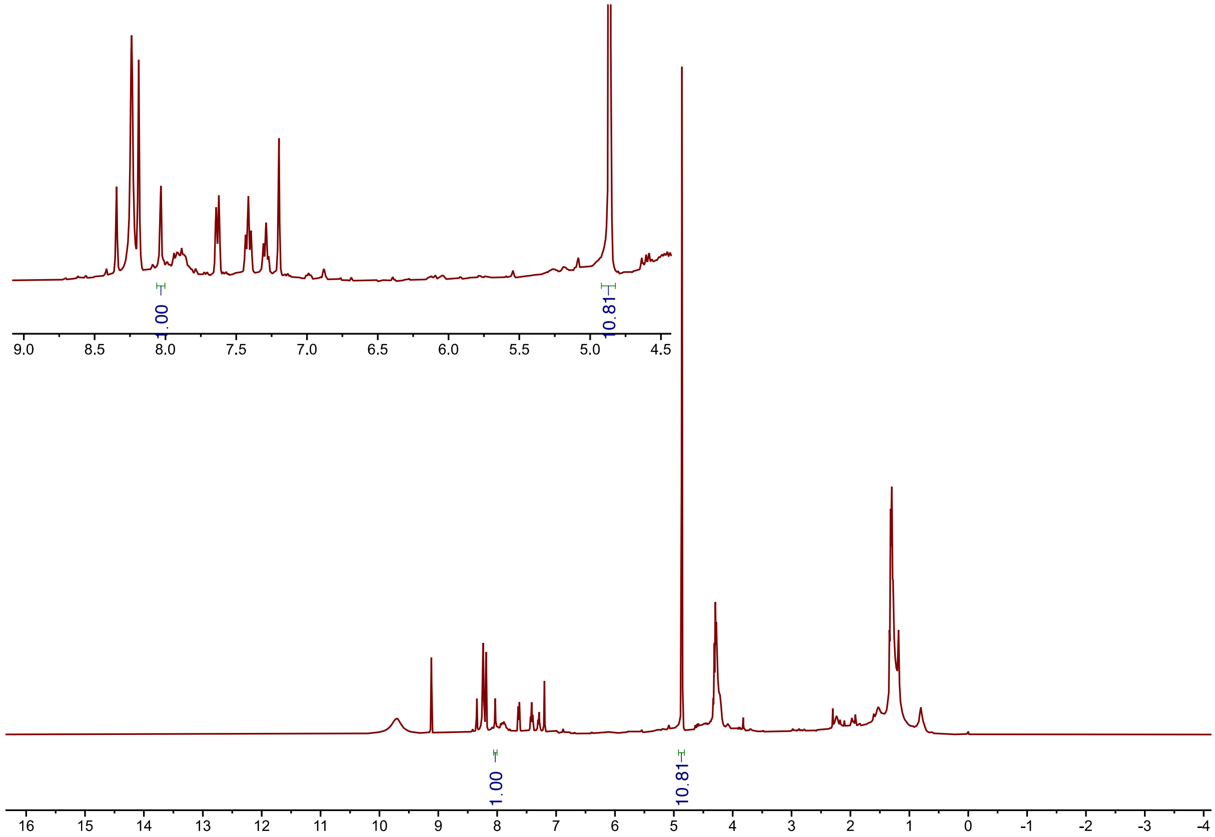

Figure S70. ^1H NMR spectrum of the photooxidation reaction mediated by PC **4** (yield = 18%).

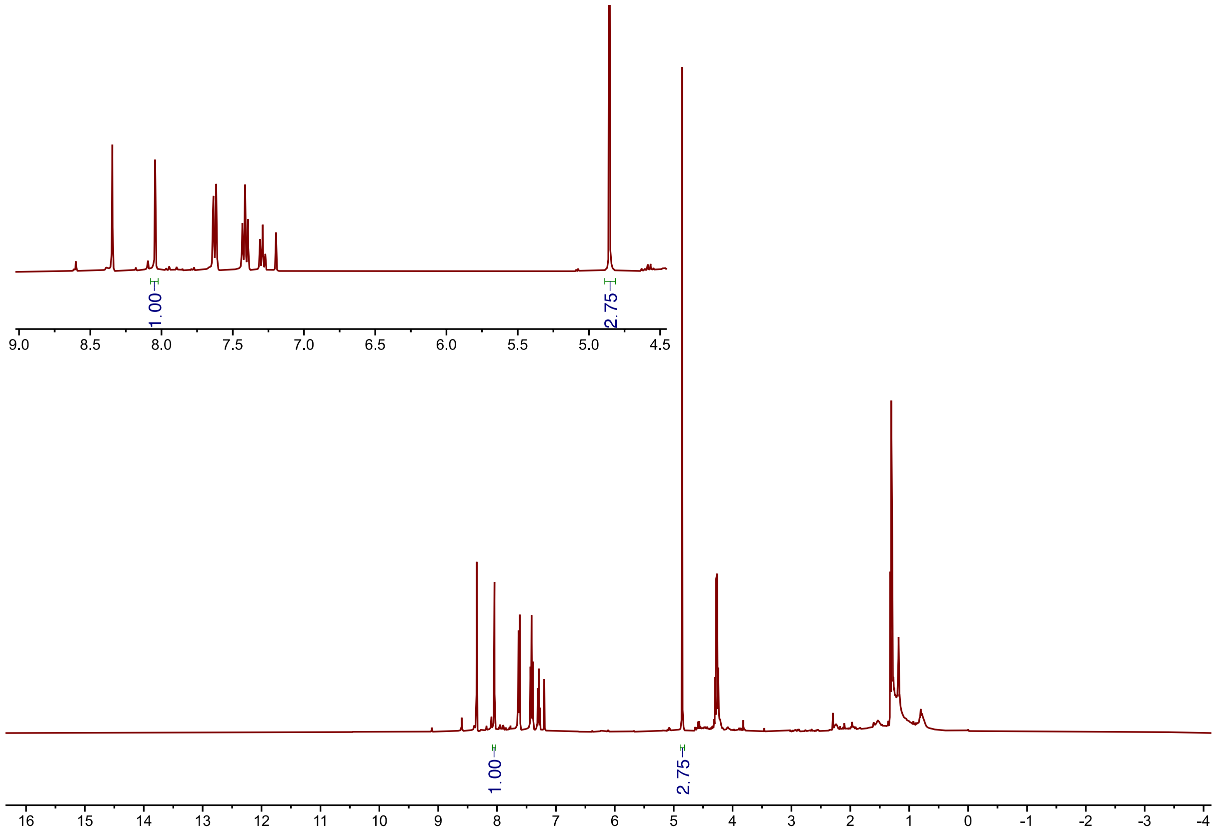

Figure S71. ^{1}H NMR spectrum of the photooxidation reaction mediated by PC **2** run simultaneously with that performed using **4** (yield = 64%).

Control NMR Spectra

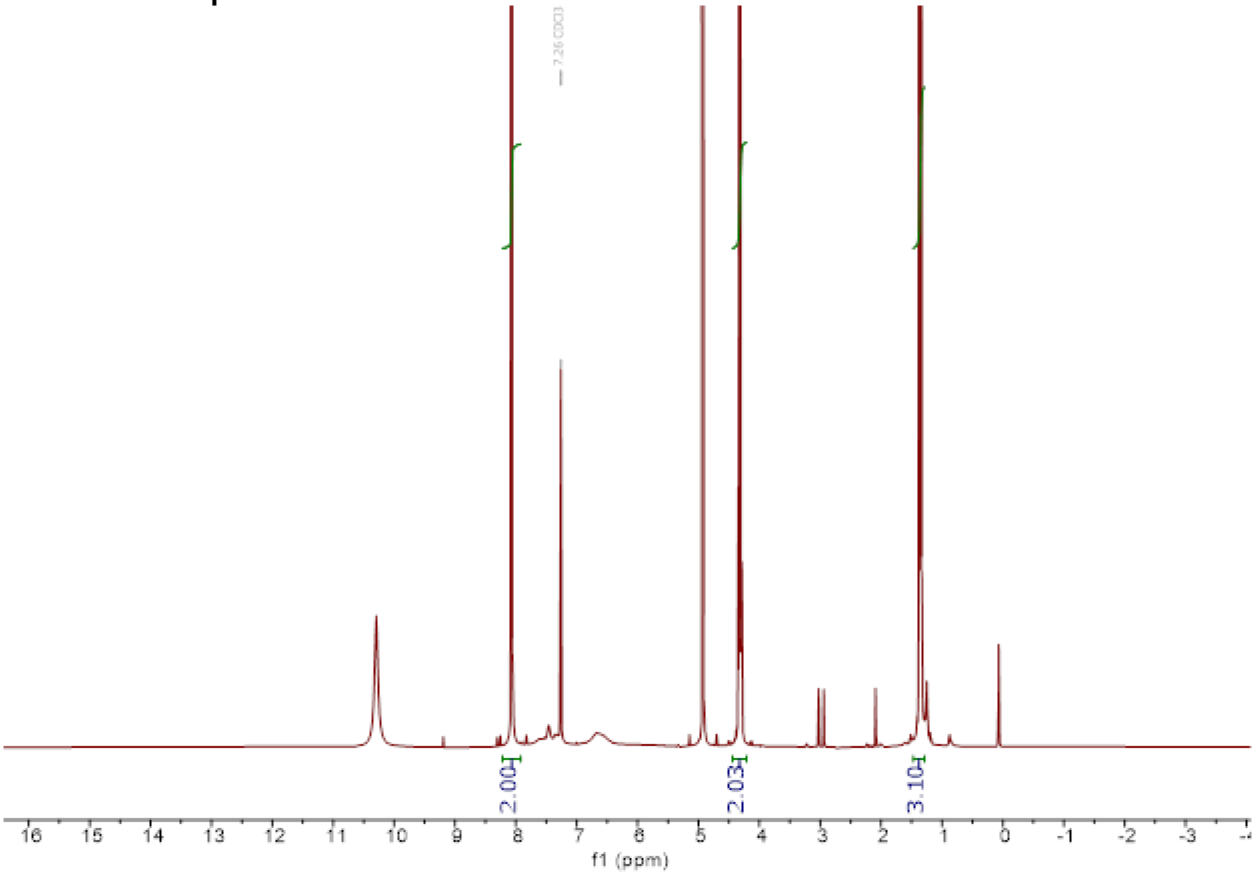

Figure S72. ^{1}H NMR spectrum of the photooxidation control reaction mediated by PC **1.** Run without light (yield = 0%).

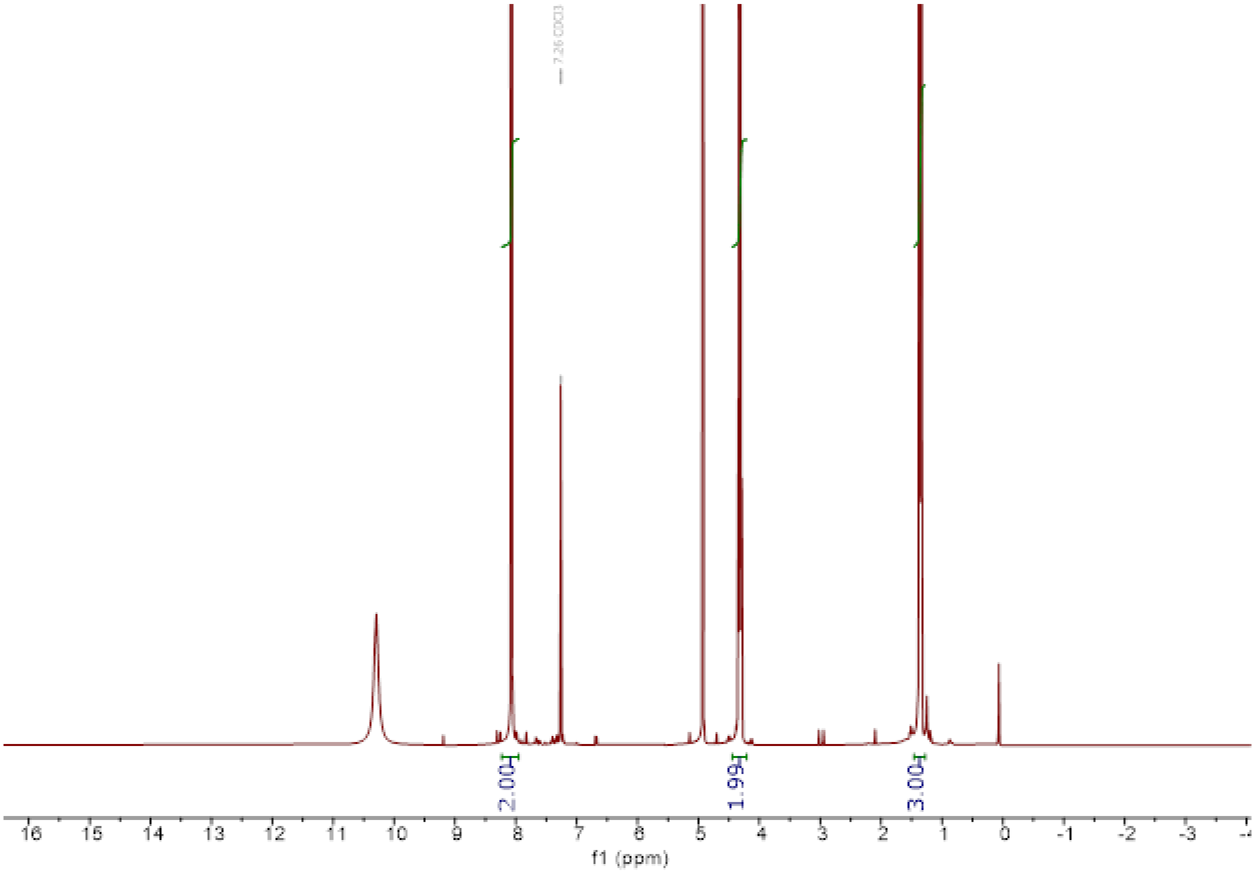

Figure S73. ^{1}H NMR spectrum of the photooxidation control reaction mediated by PC **2.** Run without light (yield = 0%).

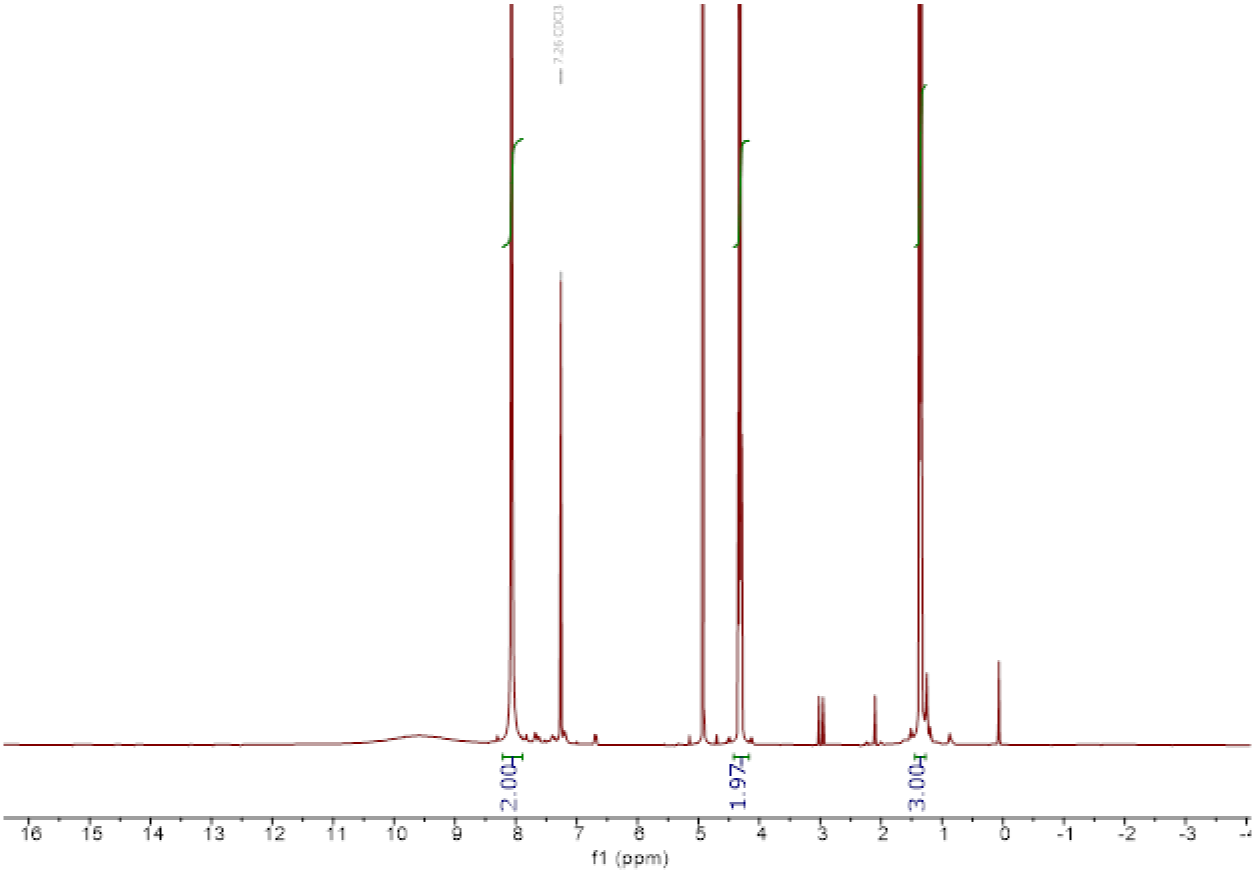

Figure S74. ^{1}H NMR spectrum of the photooxidation control reaction mediated by PC **3.** Run without light (yield = 0%).

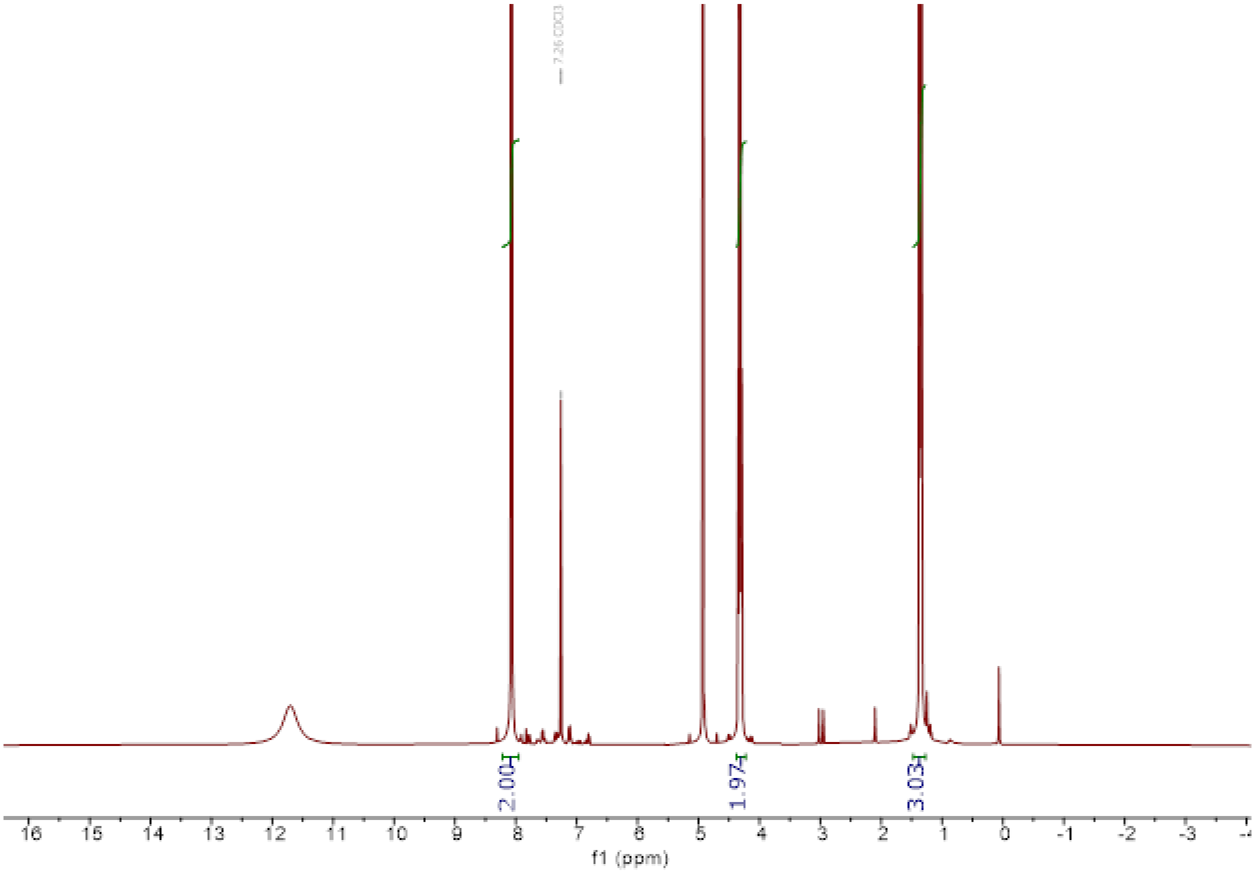

Figure S75. ^{1}H NMR spectrum of the photooxidation control reaction mediated by PC **4.** Run without light (yield = 0%).

10. Computational Details and Data

All DFT calculations in this work were performed using resources provided by the Extreme Science and Engineering Discovery Environment (XSEDE). Resources used in this work include the Comet and Expanse supercomputers run by the San Diego Supercomputer Center. Calculations were performed using the computational chemistry software package Gaussian 16 version C.01.[9]

Computational Details

All geometries were computed at uM06/LANL2DZ/CPCM-H_2O or uM06/6-31+G(d,p)/CPCM-H_2O level of theory.[10-13] Since the 6-31+G(d,p) basis set is not suitable for Te, LANL2DZ was used predominately in this work. For Se and Te, the atomic radii had to be defined and were set as 1.03 Å and 1.23 Å, respectively. For structures optimized using the 6-31+G(d,p) basis set, energy calculations were performed at uM06/6-311+G(d,p)/CPCM-H_2O to improve accuracy.[14]

Using the structures optimized at uM06/LANL2DZ/CPCM-H_2O, singly occupied molecular orbitals (SOMOs) and other molecular orbitals were computed using single point energy calculations at uM06/LANL2DZ/CPCM-H_2O/CHELPG. These calculations were then used to generate molecular orbitals for ^{1}PC, SOMOs for ^{3}PC*, and electrostatic potential (ESP) maps for ^{1}PC and ^{3}PC*. For ESP maps, total electron densities were first plotted and then mapped with ESP derived charges to show the distribution of charges on each PC.

Reorganization energies for electron transfer from various PC states were calculated according to a modified literature procedure.[15] Using the structures optimized at uM06/LANL2DZ/CPCM-H_2O, single point energy calculations were performed at uM06/LANL2DZ/CPCM-H_2O for ^{3}PC*, ^{2}PC$^{•+}$, ^{3}PC* using the optimized structure for ^{2}PC$^{•+}$, ^{2}PC$^{•+}$ using the optimized structure for ^{3}PC*, ^{1}PC, ^{1}PC using the optimized structure for ^{2}PC$^{•+}$, and ^{2}PC$^{•+}$ using the optimized structure for ^{1}PC. Reorganization energies were then calculation according to Equation S4, where E_0^+ is the radical cation energy calculated using the neutral state's optimized geometry, E_+^+ is the radical cation energy calculated using the optimized radical cation geometry, E_+^0 is the neutral state's energy calculated at the optimized radical cation geometry, and E_0^0 is the neutral state's energy calculated at its own optimized geometry.

$$\lambda\,[kcal\ mol^{-1}] = \left(E_0^{+}-E_{+}^{+}\right) + \left(E_{+}^{0}-E_{0}^{0}\right)\cdot 627.51\,[kcal\ mol^{-1}\ hartrees^{-1}] \qquad \text{Eq. (S4)}$$

Finally, UV-visible absorption spectra and transitions were computed at rCAM-B3LYP/LANL2DZ/CPCM-H_2O.[16]

Results

Crystal Structure

Computed Structure

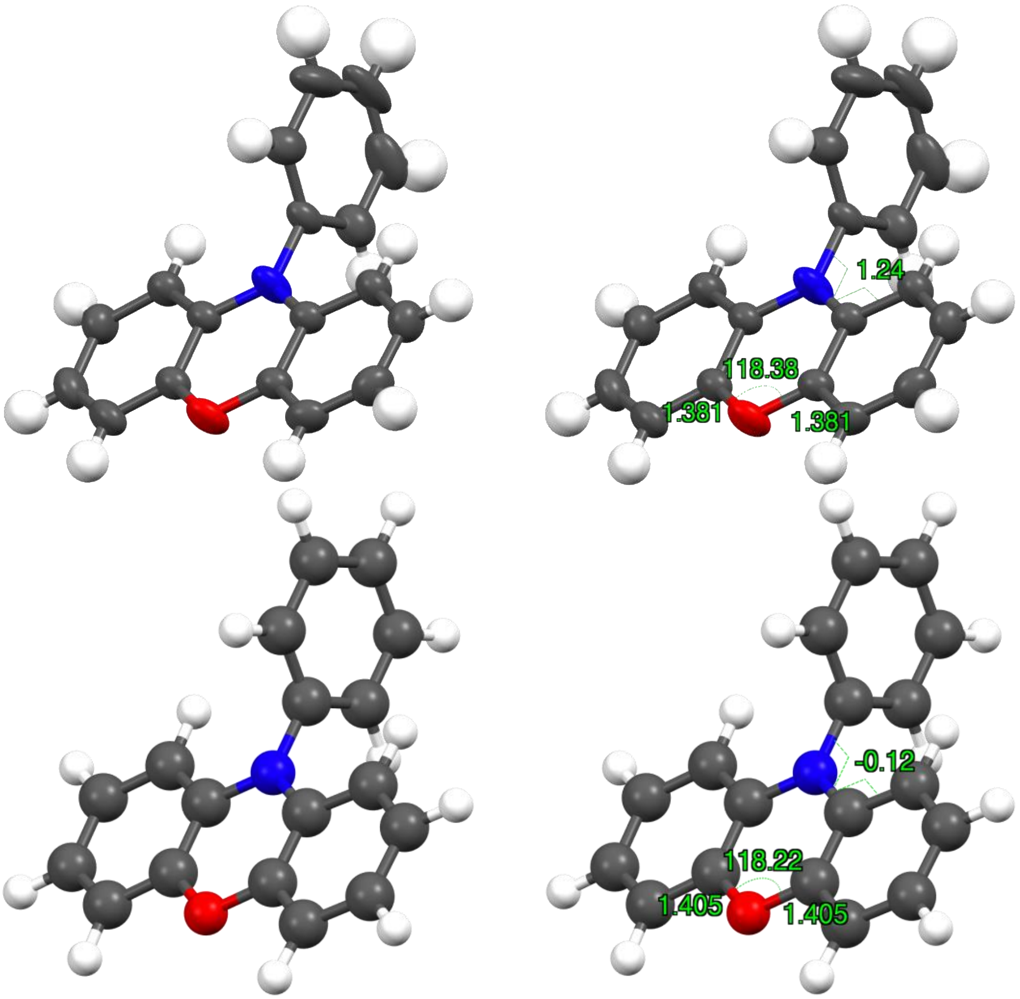

Figure S76. Comparison of the crystal structure (top) and computed structure (bottom) of PC **1**.

Crystal Structure

Computed Structure

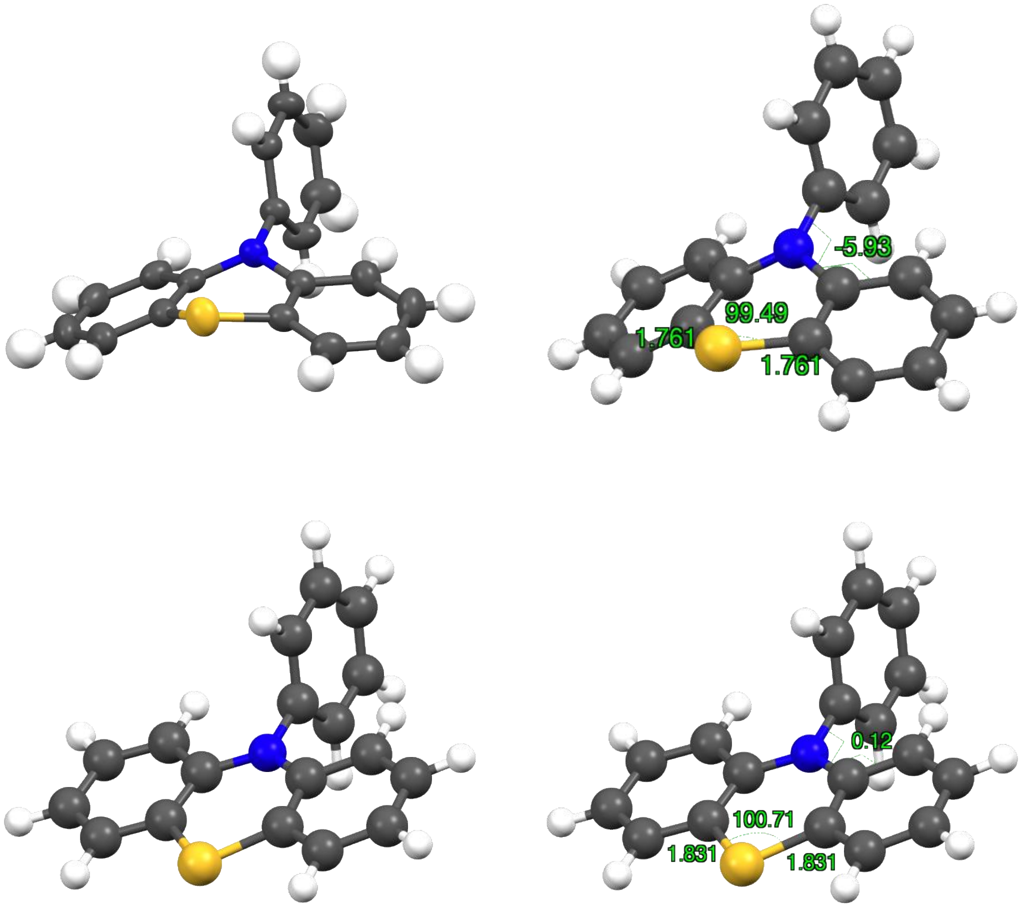

Figure S77. Comparison of the crystal structure (top) and computed structure (bottom) of PC **2**.

Crystal Structure

Computed Structure

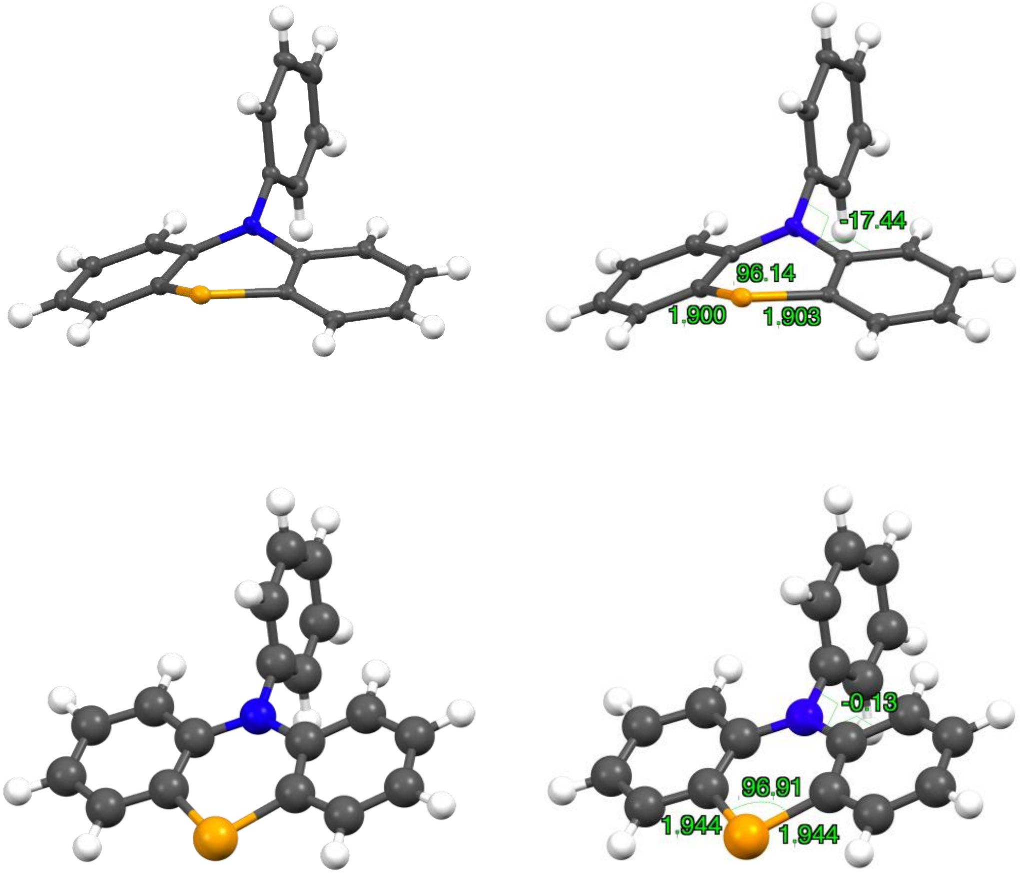

Figure S78. Comparison of the crystal structure (top) and computed structure (bottom) of PC **3**.

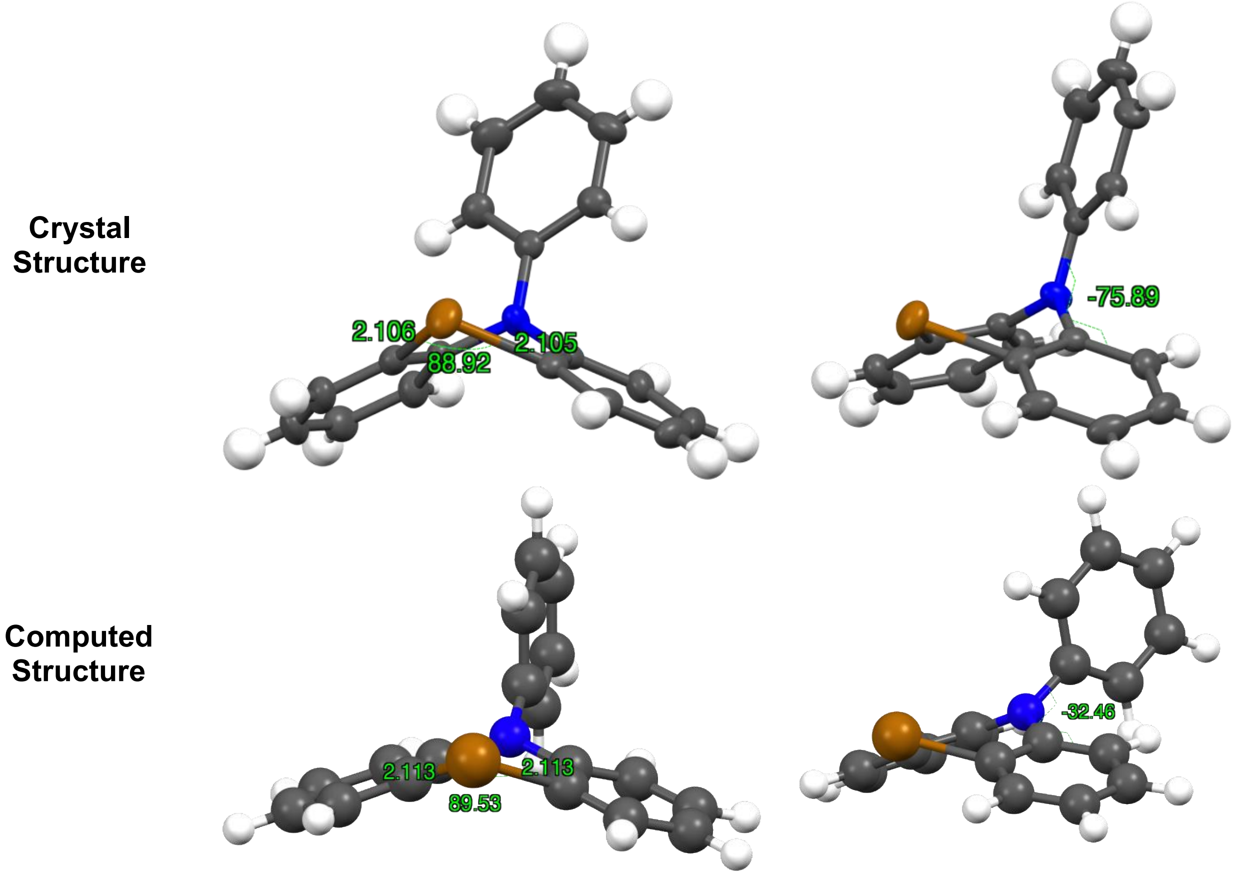

Figure S79. Comparison of the crystal structure (top) and computed structure (bottom) of PC **4**.

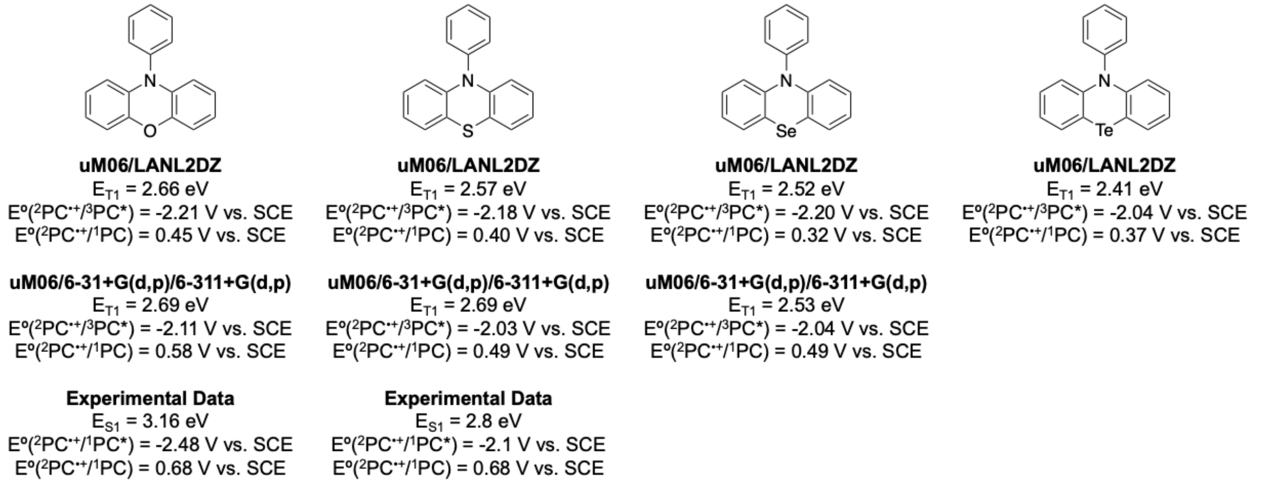

Figure S80. Computed redox properties for PCs **1** – **4** compared to previously reported computational[17] and experimental[17,18] results for PCs **1** and **2**.

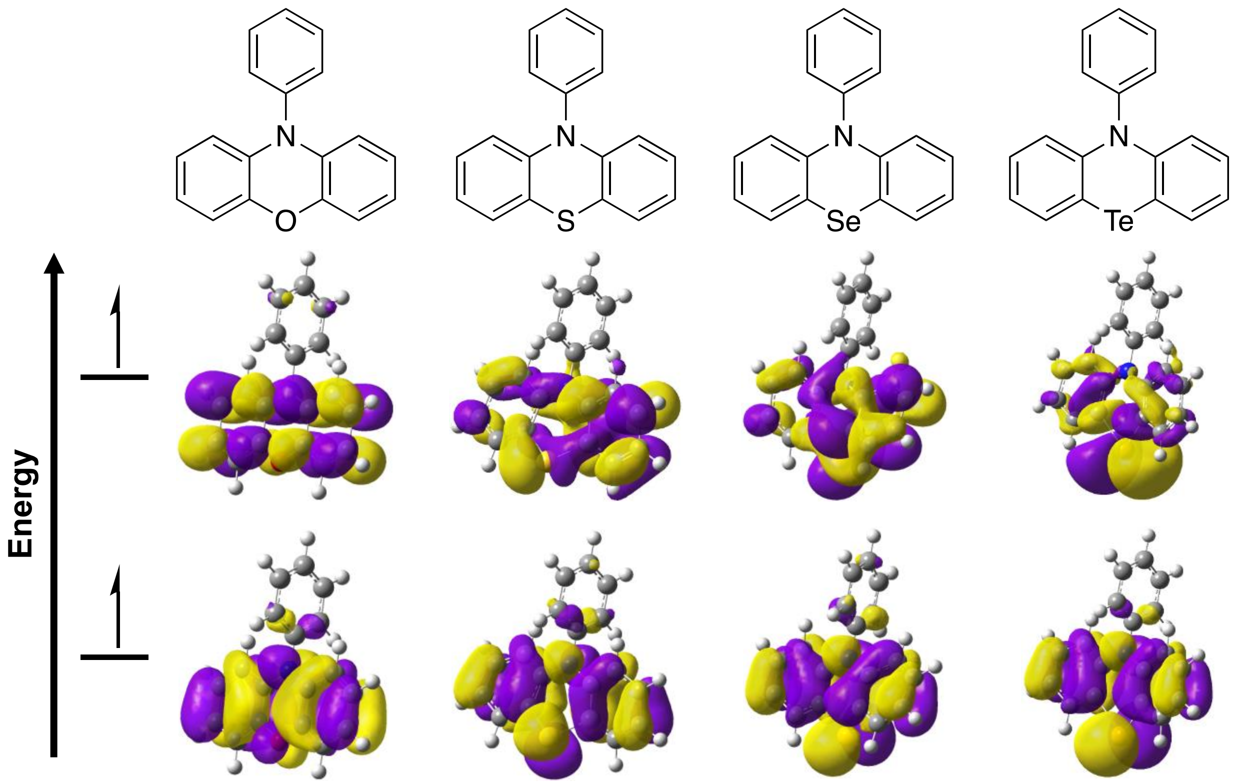

Figure S81. Computed SOMOs for PCs **1 – 4**.

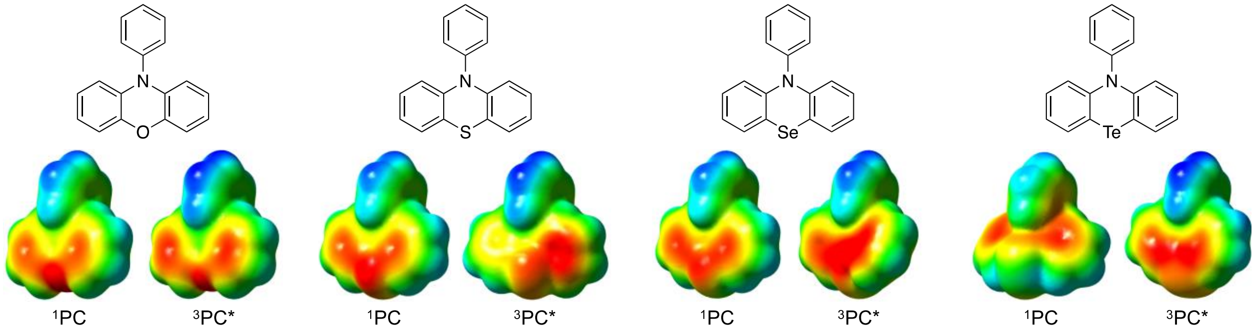

Figure S82. Computed ESP maps for PCs **1 – 4**.

Table S12. Single point energies used in the calculation of reorganization energies for PCs **1** – **4**.

PC	Transition	E_0^+	E_+^+	E_+^0	E_0^0	λ (kcal/mol)
**1**	$^3PC^* \rightarrow {}^2PC^{\bullet +}$	-822.814	-822.81811	-822.89308	-822.89719	5.2
	$^2PC^{\bullet +} \rightarrow {}^1PC$	-822.81492	-822.81811	-822.99845	-823.00176	4.1
**2**	$^3PC^* \rightarrow {}^2PC^{\bullet +}$	-757.69496	-757.71729	-757.7939	-757.79975	17.7
	$^2PC^{\bullet +} \rightarrow {}^1PC$	-757.71426	-757.71729	-757.89833	-757.90147	3.9
**3**	$^3PC^* \rightarrow {}^2PC^{\bullet +}$	-756.81528	-756.83756	-756.91133	-756.91945	19.1
	$^2PC^{\bullet +} \rightarrow {}^1PC$	-756.83456	-756.83756	-757.01609	-757.01916	3.8
**4**	$^3PC^* \rightarrow {}^2PC^{\bullet +}$	-755.64601	-755.66154	-755.73155	-755.75142	22.2
	$^2PC^{\bullet +} \rightarrow {}^1PC$	-755.64903	-755.66154	-755.8365	-755.84288	11.9

For computed UV-vis of PCs **1** – **4**, the first 10 excited states of each PC are reported below, with dominant transitions (i.e. exhibiting significant oscillator strengths, f values) highlighted in blue.

*For PC **1***

```
 Excited State   1:      Singlet-?Sym    3.9507 eV  313.83 nm  f=0.0329  <S**2>=0.000
      68 -> 69         0.60213
      68 -> 71         0.32693
      68 -> 73         0.12996

 Excited State   2:      Singlet-?Sym    4.3359 eV  285.95 nm  f=0.2323  <S**2>=0.000
      67 -> 69        -0.15122
      68 -> 69        -0.25463
      68 -> 71         0.56682
      68 -> 73        -0.25417

 Excited State   3:      Singlet-?Sym    4.4371 eV  279.43 nm  f=0.0003  <S**2>=0.000
      67 -> 70         0.10334
      68 -> 70         0.68390

 Excited State   4:      Singlet-?Sym    4.5762 eV  270.93 nm  f=0.0001  <S**2>=0.000
      63 -> 69         0.11428
      64 -> 71        -0.10376
      67 -> 74         0.12601
      68 -> 72         0.66008

 Excited State   5:      Singlet-?Sym    4.9593 eV  250.00 nm  f=0.0221  <S**2>=0.000
      67 -> 71         0.14625
      68 -> 69        -0.24546
      68 -> 71         0.20480
      68 -> 73         0.59852

 Excited State   6:      Singlet-?Sym    5.5089 eV  225.06 nm  f=0.0019  <S**2>=0.000
      63 -> 70         0.25374
```

65 -> 69 -0.33830
65 -> 71 0.24162
65 -> 73 0.25381
66 -> 70 0.44230

Excited State 7: Singlet-?Sym 5.7770 eV 214.62 nm f=1.1102 <S**2>=0.000
63 -> 74 0.11953
64 -> 72 0.10555
67 -> 69 0.52146
67 -> 71 0.33548
67 -> 73 0.10559
68 -> 71 0.13411
68 -> 73 -0.19915

Excited State 8: Singlet-?Sym 5.8715 eV 211.16 nm f=0.2177 <S**2>=0.000
65 -> 70 0.11381
67 -> 72 -0.20714
68 -> 74 0.64084

Excited State 9: Singlet-?Sym 6.2957 eV 196.93 nm f=0.0384 <S**2>=0.000
63 -> 72 -0.14244
64 -> 72 0.11374
66 -> 72 0.11421
67 -> 69 -0.30659
67 -> 71 0.50992
67 -> 73 -0.27751

Excited State 10: Singlet-?Sym 6.3012 eV 196.76 nm f=0.0022 <S**2>=0.000
63 -> 69 0.17402
63 -> 71 -0.11437
63 -> 73 -0.10353
65 -> 70 0.48209
66 -> 69 0.33154
66 -> 71 -0.22267
66 -> 73 -0.19965

*For PC **2***

Excited State 1: Singlet-?Sym 3.9132 eV 316.84 nm f=0.0333 <S**2>=0.000
67 -> 68 0.63042
67 -> 69 0.27900
67 -> 72 0.10657

Excited State 2: Singlet-?Sym 4.3312 eV 286.26 nm f=0.1680 <S**2>=0.000
66 -> 68 0.17503
67 -> 68 -0.21650
67 -> 69 0.58593
67 -> 72 -0.23428

Excited State 3: Singlet-?Sym 4.4401 eV 279.24 nm f=0.0234 <S**2>=0.000
62 -> 68 -0.10144

63 -> 69 0.10232
66 -> 73 -0.11272
67 -> 71 0.66855

Excited State 4: Singlet-?Sym 4.5271 eV 273.87 nm f=0.0000 <S**2>=0.000
66 -> 70 -0.15080
67 -> 70 0.67826

Excited State 5: Singlet-?Sym 4.9519 eV 250.38 nm f=0.0000 <S**2>=0.000
61 -> 74 0.10152
66 -> 74 0.21353
67 -> 74 0.66239

Excited State 6: Singlet-?Sym 5.0377 eV 246.11 nm f=0.0034 <S**2>=0.000
66 -> 69 -0.19805
67 -> 68 -0.20587
67 -> 69 0.21898
67 -> 72 0.58318

Excited State 7: Singlet-?Sym 5.4853 eV 226.03 nm f=0.0012 <S**2>=0.000
62 -> 70 -0.26620
64 -> 68 -0.31147
64 -> 69 0.28535
64 -> 72 0.26009
65 -> 70 0.42721

Excited State 8: Singlet-?Sym 5.5385 eV 223.86 nm f=1.0984 <S**2>=0.000
66 -> 68 0.56825
66 -> 69 0.24318
67 -> 69 -0.12015
67 -> 72 0.24521

Excited State 9: Singlet-?Sym 5.7755 eV 214.67 nm f=0.1023 <S**2>=0.000
66 -> 71 0.36421
67 -> 73 0.55534

Excited State 10: Singlet-?Sym 6.0565 eV 204.71 nm f=0.0040 <S**2>=0.000
62 -> 71 -0.11370
65 -> 71 -0.12314
66 -> 68 -0.23350
66 -> 69 0.56821
66 -> 72 -0.24790
67 -> 72 0.10930

*For PC **3***
Excited State 1: Singlet-?Sym 3.8989 eV 318.00 nm f=0.0300 <S**2>=0.000
67 -> 68 0.61994
67 -> 69 0.30119
67 -> 72 0.10722

Excited State 2: Singlet-?Sym 4.1188 eV 301.02 nm f=0.0000 <S**2>=0.000
61 -> 73 0.10066
66 -> 73 0.22195
67 -> 73 0.66172

Excited State 3: Singlet-?Sym 4.3068 eV 287.88 nm f=0.1522 <S**2>=0.000
66 -> 68 0.17758
67 -> 68 -0.23547
67 -> 69 0.57854
67 -> 72 -0.23428

Excited State 4: Singlet-?Sym 4.3901 eV 282.42 nm f=0.0370 <S**2>=0.000
66 -> 74 -0.10392
67 -> 71 0.67120

Excited State 5: Singlet-?Sym 4.5120 eV 274.79 nm f=0.0000 <S**2>=0.000
66 -> 70 -0.17746
67 -> 70 0.67208

Excited State 6: Singlet-?Sym 5.0215 eV 246.91 nm f=0.0013 <S**2>=0.000
66 -> 69 -0.22101
67 -> 68 -0.21333
67 -> 69 0.20686
67 -> 72 0.57323

Excited State 7: Singlet-?Sym 5.4482 eV 227.57 nm f=1.0386 <S**2>=0.000
66 -> 68 0.56884
66 -> 69 0.23891
67 -> 69 -0.10831
67 -> 72 0.25561

Excited State 8: Singlet-?Sym 5.4772 eV 226.36 nm f=0.0011 <S**2>=0.000
62 -> 70 0.27864
64 -> 68 0.32328
64 -> 69 -0.28386
64 -> 72 -0.25283
65 -> 70 0.41394

Excited State 9: Singlet-?Sym 5.6845 eV 218.11 nm f=0.0656 <S**2>=0.000
65 -> 68 -0.10290
66 -> 71 0.43813
67 -> 74 0.48674

Excited State 10: Singlet-?Sym 5.8354 eV 212.47 nm f=0.0007 <S**2>=0.000
66 -> 75 0.14003
67 -> 75 0.68086

*For PC **4***

Excited State 1: Singlet-?Sym 3.4009 eV 364.56 nm f=0.0007 <S**2>=0.000
66 -> 68 -0.19509

67 -> 68 0.62786
67 -> 69 0.17765

Excited State 2: Singlet-?Sym 4.3925 eV 282.26 nm f=0.0337 <S**2>=0.000
66 -> 68 0.42160
67 -> 68 0.27955
67 -> 69 -0.38112
67 -> 73 0.26007

Excited State 3: Singlet-?Sym 4.5662 eV 271.53 nm f=0.0560 <S**2>=0.000
64 -> 72 -0.11798
66 -> 70 0.10852
66 -> 74 -0.10006
67 -> 70 0.65225

Excited State 4: Singlet-?Sym 4.7150 eV 262.95 nm f=0.0887 <S**2>=0.000
64 -> 74 -0.11544
65 -> 70 0.11203
66 -> 68 0.18824
66 -> 73 0.11546
67 -> 69 0.11751
67 -> 71 0.13289
67 -> 72 0.60076

Excited State 5: Singlet-?Sym 4.8443 eV 255.94 nm f=0.0521 <S**2>=0.000
66 -> 68 0.32918
66 -> 69 0.29051
67 -> 69 0.46521
67 -> 71 -0.19480
67 -> 72 -0.11681

Excited State 6: Singlet-?Sym 5.0945 eV 243.37 nm f=0.1039 <S**2>=0.000
66 -> 68 -0.28588
66 -> 71 -0.18686
67 -> 71 -0.37637
67 -> 72 0.20087
67 -> 73 0.41236

Excited State 7: Singlet-?Sym 5.2208 eV 237.48 nm f=0.0343 <S**2>=0.000
66 -> 68 -0.13504
66 -> 69 0.16474
66 -> 71 0.18313
67 -> 69 0.11154
67 -> 71 0.46430
67 -> 73 0.40450

Excited State 8: Singlet-?Sym 5.3106 eV 233.46 nm f=0.0838 <S**2>=0.000
66 -> 70 0.40154
67 -> 70 -0.15116
67 -> 74 0.51924

Excited State   9:   Singlet-?Sym   5.4838 eV 226.09 nm f=0.0107 <S**2>=0.000
62 -> 69   0.17389
62 -> 71   0.20865
62 -> 73   0.10278
63 -> 69   0.34529
63 -> 71   -0.28977
63 -> 73   0.13065
65 -> 68   0.14137
65 -> 69   -0.21484
65 -> 71   -0.29114

Excited State 10:   Singlet-?Sym   5.5366 eV 223.93 nm f=0.1135 <S**2>=0.000
64 -> 68   -0.14808
66 -> 70   -0.35818
66 -> 74   0.11635
66 -> 75   -0.12006
67 -> 74   0.39572
67 -> 75   0.33502

Excited State 11:   Singlet-?Sym   5.5611 eV 222.95 nm f=0.3977 <S**2>=0.000
66 -> 69   0.40230
66 -> 72   0.25086
66 -> 73   -0.37240
67 -> 68   0.12600
67 -> 69   -0.10682
67 -> 72   0.17712

Molecular coordinates

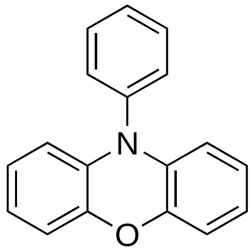

Basis set: LANL2DZ
PC State: neutral singlet
E_{0K} (not ZPE and thermally corrected) = -823.001758 hartrees
H (298 K) = -822.724019 hartrees
G (298 K) = -822.783277 hartrees

C	-6.77561000	-3.40521200	0.16697700
C	-5.37385100	-3.35412200	0.07778100
C	-4.72799900	-2.12492200	0.03751700
C	-5.44385600	-0.90887700	0.08374300
C	-6.84298200	-0.97092500	0.17263700
C	-7.50308700	-2.21017000	0.21407400
C	-3.33170500	0.30936000	-0.05454300
C	-2.64184800	-0.92158400	-0.09804500

C	-1.25669200	-0.97921300	-0.18726100
H	-0.77884800	-1.95352400	-0.21720600
C	-0.50875600	0.20983100	-0.23657600
C	-1.17468600	1.44027800	-0.19438500
C	-2.57561200	1.49061400	-0.10388800
H	-7.28254500	-4.36351000	0.19844100
H	-4.77284300	-4.25730900	0.03859200
H	-7.41694100	-0.04973400	0.20906200
H	-8.58590900	-2.23211100	0.28270100
H	0.57275400	0.16773900	-0.30623800
H	-0.61210900	2.36755600	-0.23102600
H	-3.08240300	2.45055900	-0.07139500
C	-5.45461600	1.55739900	0.07863700
C	-5.85161700	2.16877300	-1.11891900
C	-5.74781000	2.14858900	1.31559200
C	-6.54841000	3.38503200	-1.07781500
H	-5.61170300	1.68940100	-2.06438900
C	-6.44562600	3.36440800	1.35390700
H	-5.42899500	1.65365600	2.22916200
C	-6.84513600	3.98224300	0.15795500
H	-6.85791200	3.86323600	-2.00160300
H	-6.67569300	3.82679200	2.30844100
H	-7.38476300	4.92355600	0.18871800
N	-4.73934700	0.31239900	0.03743000
O	-3.32564000	-2.14861700	-0.05235700

PC State: neutral triplet
E_{0K} (not ZPE and thermally corrected) = -822.897185 hartrees
H (298 K) = -822.625477 hartrees
G (298 K) = -822.685561 hartrees

C	-6.75731900	-3.41442800	0.16171600
C	-5.36955100	-3.37257900	0.07564400
C	-4.70966500	-2.13857800	0.03447400
C	-5.44958900	-0.88395000	0.08027900
C	-6.86759100	-0.95506800	0.16762100
C	-7.51129200	-2.17840300	0.20782500
C	-3.35141700	0.32644700	-0.05224300
C	-2.63983800	-0.94384400	-0.09626100
C	-1.24338700	-0.99135000	-0.18363500
H	-0.75983300	-1.96343100	-0.21359300
C	-0.51078400	0.19031200	-0.22998300
C	-1.19915600	1.46341100	-0.18693900
C	-2.57835800	1.51960400	-0.10052300
H	-7.27554000	-4.36676100	0.19395700
H	-4.77305200	-4.27947900	0.03862200
H	-7.44413000	-0.03470200	0.20266600
H	-8.59369900	-2.21485200	0.27449700
H	0.57137900	0.16130000	-0.29774800
H	-0.62458300	2.38316100	-0.22280500
H	-3.08347700	2.48114400	-0.06891900

C	-5.45651900	1.55965200	0.07741800
C	-5.86000100	2.15320500	-1.12302000
C	-5.72894300	2.14581300	1.31775500
C	-6.55636400	3.37000600	-1.07792500
H	-5.62941100	1.66681000	-2.06679500
C	-6.42587400	3.36261600	1.35462100
H	-5.39902500	1.65377700	2.22851900
C	-6.83791700	3.97245100	0.15883100
H	-6.87611600	3.84344700	-2.00021600
H	-6.64492700	3.83046400	2.30864600
H	-7.37668100	4.91399200	0.19057400
N	-4.73608700	0.30521700	0.03449900
O	-3.33404800	-2.13381100	-0.05183900

PC State: radical cation
E_{0K} (not ZPE and thermally corrected) = -822.81811 hartrees
H (298 K) = -822.539348 hartrees
G (298 K) = -822.597917 hartrees

C	-6.75095500	-3.38592400	0.16974100
C	-5.36037300	-3.35753700	0.08367200
C	-4.71560300	-2.12268000	0.03953100
C	-5.43812600	-0.89906800	0.08040500
C	-6.84656900	-0.94827800	0.16683200
C	-7.48906800	-2.17880300	0.21076800
C	-3.34329100	0.30921300	-0.05700200
C	-2.64976500	-0.93098400	-0.09704300
C	-1.26013600	-0.99228500	-0.18562300
H	-0.77759400	-1.96215900	-0.21336700
C	-0.53767500	0.19837500	-0.23537200
C	-1.20902700	1.44371700	-0.19530100
C	-2.59381300	1.50486500	-0.10719500
H	-7.26942000	-4.33677100	0.20470900
H	-4.76522800	-4.26243400	0.04943700
H	-7.41723500	-0.02695700	0.19723000
H	-8.57005200	-2.21373000	0.27668700
H	0.54331800	0.17029000	-0.30439700
H	-0.63616300	2.36264700	-0.23358200
H	-3.10239400	2.46185900	-0.07676800
C	-5.45641900	1.56015400	0.07606700
C	-5.84295000	2.15963700	-1.12723000
C	-5.74152200	2.13114700	1.32094800
C	-6.53934100	3.37476100	-1.07719100
H	-5.60318100	1.68383100	-2.07355000
C	-6.43825700	3.34653100	1.35697100
H	-5.42435400	1.63378800	2.23279800
C	-6.83527300	3.96566300	0.16132000
H	-6.84808800	3.85560200	-1.99885000
H	-6.66865500	3.80582000	2.31195200
H	-7.37430500	4.90649700	0.19462800
N	-4.73310700	0.30102000	0.03168000

O	-3.33817400	-2.12621800	-0.04718100

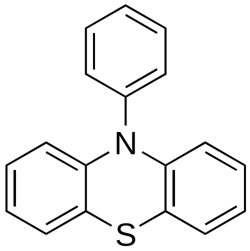

Basis set: LANL2DZ
PC State: neutral singlet
E_{0K} (not ZPE and thermally corrected) = -757.901466 hartrees
H (298 K) = -757.626528 hartrees
G (298 K) = -757.683229 hartrees

C	-7.06210200	-3.34084600	0.17696200
C	-5.66712500	-3.43794200	0.09309900
C	-4.86705000	-2.29126400	0.04340800
C	-5.43726600	-1.00039900	0.07605500
C	-6.84567500	-0.92053500	0.16047800
C	-7.64426500	-2.06853300	0.21011300
C	-3.25615100	0.25736200	-0.05813300
C	-2.42827200	-0.88486400	-0.10713900
C	-1.03664500	-0.76747500	-0.19207100
H	-0.43143700	-1.67048700	-0.22783900
C	-0.42002400	0.48989800	-0.23094600
C	-1.22595600	1.63306000	-0.18312800
C	-2.61792200	1.51760200	-0.09845300
H	-7.67160900	-4.23695400	0.21482200
H	-5.19133200	-4.41559200	0.06516800
H	-7.32455000	0.05226700	0.18782900
H	-8.72213200	-1.95947800	0.27467400
H	0.65957800	0.56783800	-0.29673300
H	-0.77896000	2.62161500	-0.21150300
H	-3.21725700	2.42090100	-0.06294700
C	-5.38810600	1.44143600	0.07240000
C	-5.80661600	2.04188600	-1.12208000
C	-5.65831100	2.04142600	1.30920700
C	-6.50368100	3.25794700	-1.07835600
H	-5.58356700	1.55435700	-2.06759100
C	-6.35556800	3.25744800	1.35089600
H	-5.32190800	1.55366600	2.22044200
C	-6.77800000	3.86556600	0.15766100
H	-6.83033700	3.72801200	-2.00040200
H	-6.56773500	3.72719100	2.30598200
H	-7.31751100	4.80682700	0.19073400
N	-4.66929900	0.18996700	0.02827900
S	-3.06600400	-2.60031200	-0.06516800

PC State: neutral triplet
E_{0K} (not ZPE and thermally corrected) = -757.799745 hartrees
H (298 K) = -757.527211 hartrees
G (298 K) = -757.588633 hartrees

C	-7.00256500	-3.33445200	0.34679000
C	-5.61743600	-3.40423600	0.48236700
C	-4.81708800	-2.25648400	0.33940000
C	-5.41114000	-0.98639000	0.07836100
C	-6.82671000	-0.94332400	-0.07263300
C	-7.60178800	-2.08693600	0.05907400
C	-3.27853800	0.29389500	-0.03260100
C	-2.51041400	-0.93085900	-0.33581300
C	-1.10958400	-0.79890200	-0.63666500
H	-0.55008600	-1.68904200	-0.91865300
C	-0.48644700	0.42337200	-0.54925300
C	-1.22237100	1.60372800	-0.11464700
C	-2.58982400	1.51952900	0.12682800
H	-7.60753800	-4.22891400	0.44621100
H	-5.13400900	-4.35668400	0.68537100
H	-7.30856900	0.00319900	-0.29440400
H	-8.67765700	-2.01666400	-0.06356900
H	0.56531900	0.51788100	-0.80422100
H	-0.70477100	2.55035000	-0.00203500
H	-3.14145400	2.41755900	0.39318400
C	-5.40832100	1.45824600	0.07227300
C	-5.59122100	2.19544800	-1.10336100
C	-5.90520400	1.91061200	1.30032400
C	-6.29017500	3.40924600	-1.04782100
H	-5.18924100	1.81770800	-2.03946200
C	-6.60169500	3.12648300	1.34983800
H	-5.74605600	1.31499500	2.19513500
C	-6.79450900	3.87461100	0.17749400
H	-6.44174100	3.98662400	-1.95387400
H	-6.98895700	3.48819700	2.29669800
H	-7.33459100	4.81505100	0.21827100
N	-4.67389600	0.20900200	0.01868800
S	-3.03451900	-2.47126000	0.51842300

PC State: radical cation
E_{0K} (not ZPE and thermally corrected) = -757.717292 hartrees
H (298 K) = -757.440438 hartrees
G (298 K) = -757.499699 hartrees

C	-7.02930700	-3.32626100	0.17443000
C	-5.64584100	-3.43681800	0.09836800
C	-4.84471300	-2.28312400	0.04924700
C	-5.42838100	-0.98642700	0.07705300
C	-6.84392100	-0.90077200	0.15561300
C	-7.62369500	-2.04551400	0.20275300

C	-3.27119700	0.25701200	-0.05805800
C	-2.44525900	-0.89995900	-0.10326000
C	-1.04714400	-0.78605300	-0.19124100
H	-0.44056400	-1.68589600	-0.22479500
C	-0.44764600	0.46735900	-0.23429400
C	-1.25450600	1.62550400	-0.18893100
C	-2.63413100	1.52559800	-0.10316800
H	-7.64476000	-4.21714400	0.21125800
H	-5.17378100	-4.41421300	0.07540000
H	-7.32269100	0.07034600	0.17822900
H	-8.70171900	-1.95104700	0.26167000
H	0.63034200	0.55230900	-0.30179500
H	-0.79483000	2.60641600	-0.22146600
H	-3.23185000	2.42804400	-0.06990400
C	-5.39263800	1.45098700	0.07396200
C	-5.80061900	2.03988200	-1.12671800
C	-5.65820200	2.03303800	1.31722100
C	-6.49817700	3.25453700	-1.07663900
H	-5.57527400	1.55559000	-2.07249300
C	-6.35648800	3.24774200	1.35523200
H	-5.32477400	1.54356000	2.22772700
C	-6.77490300	3.85628000	0.16127700
H	-6.82271800	3.72656700	-1.99752100
H	-6.57173600	3.71453100	2.31020700
H	-7.31511000	4.79644900	0.19537600
N	-4.66631800	0.18664900	0.02888800
S	-3.08024500	-2.57358000	-0.04790600

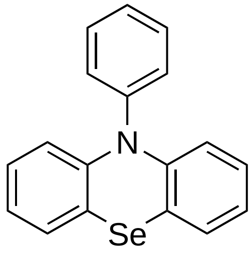

Basis set: LANL2DZ
PC State: neutral singlet
E_{0K} (not ZPE and thermally corrected) = -757.019164 hartrees
H (298 K) = -756.744911 hartrees
G (298 K) = -756.80271 hartrees

C	-7.12621800	-3.32204700	0.17942900
C	-5.73481200	-3.45217400	0.09151100
C	-4.89815000	-2.32810900	0.04078000
C	-5.43694600	-1.02213700	0.07624900
C	-6.84536100	-0.90934600	0.16574700
C	-7.67397500	-2.03476700	0.21641600
C	-3.23682600	0.24626600	-0.05900900
C	-2.38064700	-0.87679400	-0.11076100
C	-0.99007900	-0.71678100	-0.19329100

H	-0.35494800	-1.59986600	-0.23102700
C	-0.40312500	0.55408000	-0.22846900
C	-1.23872400	1.67548900	-0.17935100
C	-2.62642500	1.52323300	-0.09622200
H	-7.75823100	-4.20259300	0.21772700
H	-5.29197600	-4.44602100	0.06145000
H	-7.30311600	0.07318400	0.19652000
H	-8.74830600	-1.89585700	0.28481200
H	0.67443800	0.65901800	-0.29269400
H	-0.81874100	2.67603500	-0.20529000
H	-3.24430900	2.41355500	-0.05962700
C	-5.37220400	1.41434200	0.07074500
C	-5.79296600	2.01302700	-1.12364700
C	-5.64092100	2.01628600	1.30675100
C	-6.49075500	3.22869900	-1.08088800
H	-5.57110500	1.52400400	-2.06874700
C	-6.33882200	3.23193800	1.34792900
H	-5.30276200	1.52994700	2.21818300
C	-6.76369700	3.83818800	0.15455500
H	-6.81913100	3.69699000	-2.00327800
H	-6.54970300	3.70282600	2.30278400
H	-7.30386700	4.77913000	0.18706400
N	-4.65261100	0.16170900	0.02712700
Se	-2.99777600	-2.71986200	-0.07776000

PC State: neutral triplet
E_{0K} (not ZPE and thermally corrected) = -756.919452 hartrees
H (298 K) = -756.647441 hartrees
G (298 K) = -756.710138 hartrees

C	-7.07450500	-3.31900400	0.03226600
C	-5.72865600	-3.41096100	-0.31936900
C	-4.88619700	-2.28205600	-0.30029800
C	-5.41106200	-1.00339700	0.05539700
C	-6.78827500	-0.93463700	0.41892300
C	-7.59772100	-2.06179100	0.40779300
C	-3.26521000	0.27910800	-0.08952200
C	-2.44966200	-0.92900100	0.10961400
C	-1.03407900	-0.76023500	0.29017300
H	-0.42840200	-1.63879600	0.50890300
C	-0.44447900	0.47651400	0.15334400
C	-1.23954900	1.63243600	-0.22574800
C	-2.61849500	1.51959200	-0.33802400
H	-7.70323400	-4.20276500	0.02966000
H	-5.30732900	-4.37403200	-0.59985400
H	-7.21361500	0.01911700	0.71332000
H	-8.64033600	-1.96981100	0.69464200
H	0.62317100	0.59419500	0.31616100
H	-0.75439400	2.58718300	-0.39933000
H	-3.20880500	2.40357800	-0.56542900
C	-5.39252800	1.43810400	0.05662300

C	-6.04735900	1.88429400	-1.09742900
C	-5.42051000	2.18246000	1.24173700
C	-6.74599700	3.09927800	-1.06170800
H	-6.00665300	1.28394900	-2.00224900
C	-6.12173400	3.39584000	1.27213600
H	-4.89874300	1.80997600	2.11905100
C	-6.78399100	3.85425100	0.12157200
H	-7.25473700	3.45530300	-1.95157000
H	-6.15317000	3.97818900	2.18712900
H	-7.32596300	4.79415300	0.14685800
N	-4.65899100	0.18701400	0.01974000
Se	-3.05158400	-2.55645500	-0.80879300

PC State: radical cation
E_{0K} (not ZPE and thermally corrected) = -756.837558 hartrees
H (298 K) = -756.561357 hartrees
G (298 K) = -756.621873 hartrees

C	-7.09244200	-3.31129900	0.17612600
C	-5.71392000	-3.45218900	0.08068500
C	-4.87662800	-2.31917900	0.03175200
C	-5.42820800	-1.00778400	0.07646900
C	-6.84238800	-0.89220900	0.17525100
C	-7.65143700	-2.01615200	0.22412000
C	-3.25362400	0.24582400	-0.05907300
C	-2.39945100	-0.89146100	-0.11218100
C	-1.00059800	-0.73526700	-0.18977100
H	-0.36442800	-1.61507800	-0.22745800
C	-0.42886200	0.53031200	-0.21969600
C	-1.26555200	1.66586100	-0.17235100
C	-2.64230400	1.52972000	-0.09376900
H	-7.72901800	-4.18727900	0.21376200
H	-5.27463700	-4.44525000	0.04328800
H	-7.30049400	0.08804100	0.21382700
H	-8.72559800	-1.89227300	0.30004500
H	0.64726200	0.64144800	-0.27977400
H	-0.83247600	2.65921500	-0.19700500
H	-3.25772500	2.41972400	-0.05781200
C	-5.37825900	1.42461400	0.06962200
C	-5.79456800	2.00677700	-1.13140500
C	-5.63623000	2.01596900	1.31004900
C	-6.49175500	3.22194500	-1.08540200
H	-5.57524200	1.51608800	-2.07536800
C	-6.33403500	3.23112600	1.34518700
H	-5.29652100	1.53244600	2.22152700
C	-6.76042300	3.83221300	0.15014800
H	-6.82218300	3.68784900	-2.00739300
H	-6.54273400	3.70427000	2.29855300
H	-7.30019400	4.77277400	0.18157300
N	-4.65154800	0.15980300	0.02638100
Se	-3.01657800	-2.68900700	-0.09418500

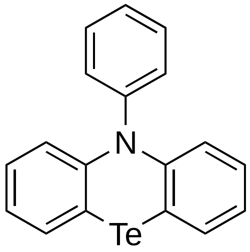

Basis set: LANL2DZ
PC State: neutral singlet
E_{0K} (not ZPE and thermally corrected) = -755.842884 hartrees
H (298 K) = -755.568399 hartrees
G (298 K) = -755.630463 hartrees

C	-7.00153200	-3.34220600	-0.14300700
C	-5.73573500	-3.37664400	0.45801900
C	-4.97015100	-2.20635000	0.59313900
C	-5.44480100	-0.97950800	0.07402200
C	-6.71888100	-0.95878100	-0.53526400
C	-7.49235100	-2.12105400	-0.62726400
C	-3.26896000	0.27450400	-0.07104800
C	-2.39524000	-0.72378000	0.41916600
C	-1.01963000	-0.66092600	0.14016000
H	-0.36151600	-1.44600100	0.50588500
C	-0.48026600	0.41477000	-0.57860500
C	-1.33714900	1.42687500	-1.03444900
C	-2.71424500	1.35033800	-0.79894500
H	-7.59252800	-4.24857100	-0.22444400
H	-5.35047000	-4.31755100	0.84487800
H	-7.10860100	-0.02792500	-0.93425300
H	-8.47236100	-2.07210800	-1.09198400
H	0.58649300	0.46298400	-0.77120400
H	-0.93862600	2.27142600	-1.58824400
H	-3.36212300	2.13386000	-1.17810100
C	-5.38930300	1.47176900	0.30532400
C	-5.86928900	2.19121700	-0.80098100
C	-5.59463500	1.95937500	1.60498400
C	-6.55301900	3.39894700	-0.60444900
H	-5.70574000	1.80285800	-1.80386100
C	-6.27950200	3.16761400	1.80039000
H	-5.21427800	1.38436600	2.44614300
C	-6.75880100	3.88831700	0.69536400
H	-6.92316200	3.95439700	-1.46031800
H	-6.43772300	3.54398300	2.80615100
H	-7.28864900	4.82374700	0.84550800
N	-4.67815000	0.22290400	0.15638100
Te	-3.16187400	-2.23125300	1.68662700

PC State: neutral triplet
E_{0K} (not ZPE and thermally corrected) = -755.751418 hartrees
H (298 K) = -755.477856 hartrees
G (298 K) = -755.541771 hartrees

C	-7.12314400	-3.29306400	0.33772600
C	-5.76460800	-3.44283200	0.07910400
C	-4.89282800	-2.33964600	-0.07822200
C	-5.43019900	-1.02285900	0.03457100
C	-6.83970500	-0.87989300	0.22254500
C	-7.66060400	-1.98778600	0.38140500
C	-3.24176000	0.23773300	-0.09747800
C	-2.38324800	-0.89435100	-0.22764700
C	-0.98359900	-0.68922900	-0.20539300
H	-0.34098800	-1.56488500	-0.31753300
C	-0.41348500	0.57162900	-0.06325100
C	-1.26808300	1.69443500	-0.00371100
C	-2.64661300	1.53561500	-0.03206000
H	-7.76104600	-4.15898100	0.48230900
H	-5.33931500	-4.44347300	-0.02152100
H	-7.28900400	0.10612200	0.25970100
H	-8.72451800	-1.84059000	0.53898800
H	0.66393000	0.69395200	-0.02006200
H	-0.85130300	2.69453000	0.06362700
H	-3.27045400	2.42122300	0.01258200
C	-5.37049000	1.40719500	0.01127300
C	-5.78492500	1.99753500	-1.18704400
C	-5.64047100	2.00593100	1.24719800
C	-6.48430500	3.21266800	-1.14703800
H	-5.55994500	1.50744600	-2.13041000
C	-6.33968700	3.22016000	1.28249900
H	-5.30471200	1.52154500	2.16039600
C	-6.76157000	3.82339400	0.08626800
H	-6.81018800	3.67846300	-2.07127700
H	-6.55397900	3.69207700	2.23577100
H	-7.30309900	4.76341600	0.11550600
N	-4.64696200	0.14760900	-0.03212800
Te	-2.91189100	-2.94350000	-0.78097700

PC State: radical cation
E_{0K} (not ZPE and thermally corrected) = -755.661536 hartrees
H (298 K) = -755.386071 hartrees
G (298 K) = -755.447903 hartrees

C	-7.18186500	-3.28711200	0.17413800
C	-5.80645000	-3.47008800	0.12619200
C	-4.91914400	-2.36975300	0.07716800
C	-5.42736900	-1.03795900	0.07775000
C	-6.84256600	-0.88006300	0.12953600
C	-7.69324100	-1.97344800	0.17537200
C	-3.22637900	0.23005000	-0.05764600

C	-2.33075300	-0.87843600	-0.09356000
C	-0.93649700	-0.66342300	-0.19525400
H	-0.26310400	-1.51645200	-0.22625500
C	-0.40602900	0.61808100	-0.25620100
C	-1.28383100	1.72024200	-0.21291700
C	-2.65383700	1.53362900	-0.11664100
H	-7.84778100	-4.14121600	0.20981300
H	-5.40688000	-4.48124300	0.12561000
H	-7.27559400	0.11180500	0.13188400
H	-8.76406200	-1.80713700	0.21186800
H	0.66472200	0.76528100	-0.33391500
H	-0.89069900	2.72982000	-0.25590900
H	-3.29358500	2.40611300	-0.08769000
C	-5.35558700	1.38798600	0.07962100
C	-5.76277300	1.98565900	-1.11710100
C	-5.62558500	1.96726400	1.32311800
C	-6.46064100	3.20013700	-1.06427900
H	-5.53509300	1.50546600	-2.06470700
C	-6.32430100	3.18184400	1.36705200
H	-5.29380000	1.47304400	2.23188900
C	-6.74075800	3.79683000	0.17562200
H	-6.78313000	3.67631400	-1.98394500
H	-6.54154000	3.64374500	2.32415900
H	-7.28132300	4.73679800	0.21305300
N	-4.62885500	0.12261600	0.03210600
Te	-2.91446500	-2.85480200	0.01578600

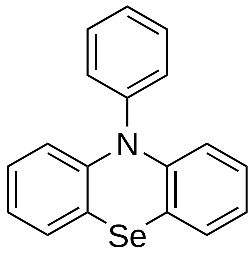

Basis set: 6-31+G(d,p) (structure optimization) and 6-311+G(d,p) (energy calculation)
PC State: neutral singlet
E_{0K} (not ZPE and thermally corrected) = -3149.525583 hartrees
H (298 K) = -3149.252373 hartrees
G (298 K) = -3149.313652 hartrees

C	-6.82737200	-3.34219900	0.51053300
C	-5.62232000	-3.35624400	-0.18507800
C	-4.95648800	-2.16586600	-0.46033600
C	-5.44464700	-0.93748300	0.00738800
C	-6.65598600	-0.93729200	0.71097200
C	-7.34225000	-2.12490700	0.94648700
C	-3.31588200	0.29183900	-0.12524500
C	-2.54067100	-0.77002800	-0.61216600
C	-1.15689900	-0.77663900	-0.46626000
H	-0.58602300	-1.62814700	-0.83169200

C	-0.50694500	0.30805400	0.11435000
C	-1.26255000	1.38529200	0.56778000
C	-2.65030100	1.37505200	0.46273300
H	-7.35661900	-4.27096000	0.70595600
H	-5.20349000	-4.29607900	-0.53972800
H	-7.07315600	-0.00191400	1.07204700
H	-8.28399400	-2.09174200	1.48882900
H	0.57569500	0.31059200	0.20803700
H	-0.77392300	2.24389400	1.02187300
H	-3.21735600	2.22292700	0.83546600
C	-5.44170800	1.50080300	-0.20990800
C	-5.83568000	2.06085200	-1.42262700
C	-5.74029700	2.14661000	0.99017000
C	-6.52930400	3.26905000	-1.43774900
H	-5.59097100	1.53870300	-2.34544600
C	-6.43333400	3.35362100	0.97367000
H	-5.42644500	1.69968400	1.93248300
C	-6.82798200	3.91520300	-0.24018500
H	-6.83597800	3.70519400	-2.38518800
H	-6.66511100	3.85620100	1.90934200
H	-7.36911800	4.85806400	-0.25153800
N	-4.72736100	0.25812100	-0.24368000
Se	-3.40833000	-2.16866600	-1.56556800

PC State: neutral triplet
E_{0K} (not ZPE and thermally corrected) = -3149.425986 hartrees
H (298 K) = -3149.157142 hartrees
G (298 K) = -3149.220764 hartrees

C	-7.07133500	-3.32118600	0.17658800
C	-5.70818100	-3.44205600	0.09208100
C	-4.86806100	-2.30912300	0.04054500
C	-5.43645100	-0.97664000	0.07589400
C	-6.85209100	-0.89128500	0.16362500
C	-7.64794100	-2.00915100	0.21283400
C	-3.27691100	0.26840900	-0.05772700
C	-2.41240500	-0.89284900	-0.10767100
C	-1.01254500	-0.73406500	-0.19049600
H	-0.39024800	-1.62835600	-0.22552900
C	-0.43203300	0.50774300	-0.22707000
C	-1.27482900	1.66588800	-0.18026600
C	-2.63952700	1.53791200	-0.09896100
H	-7.70253700	-4.20437900	0.21549700
H	-5.24879800	-4.43020400	0.06279400
H	-7.32724700	0.08478500	0.19348300
H	-8.72606100	-1.89004800	0.27992000
H	0.64744300	0.61079400	-0.29081800
H	-0.83629800	2.65981100	-0.20915400
H	-3.24320200	2.43994900	-0.06542200
C	-5.37545900	1.41936100	0.07100300
C	-5.78685100	2.00547400	-1.11898400

C	-5.63703600	2.00588200	1.30252100
C	-6.47924800	3.21335100	-1.07285000
H	-5.56258300	1.51415400	-2.06381200
C	-6.32980700	3.21374600	1.34163700
H	-5.29791800	1.51500600	2.21270400
C	-6.74943400	3.81529500	0.15565500
H	-6.80745500	3.68408500	-1.99589900
H	-6.54167200	3.68486900	2.29794000
H	-7.28981300	4.75795800	0.18892100
N	-4.65763200	0.16976200	0.02690700
Se	-3.05862600	-2.61330200	-0.07017100

PC State: radical cation
E_{0K} (not ZPE and thermally corrected) = -3149.340559 hartrees
H (298 K) = -3149.066318 hartrees
G (298 K) = -3149.126584 hartrees

C	-7.06253200	-3.29944300	0.17808100
C	-5.69465900	-3.42749700	0.08305200
C	-4.87446700	-2.29031700	0.03199200
C	-5.42700800	-0.98993500	0.07533500
C	-6.83260400	-0.88884500	0.17315500
C	-7.62521600	-2.01566900	0.22336500
C	-3.27047200	0.25373100	-0.05869100
C	-2.42616800	-0.87850600	-0.11397500
C	-1.03228600	-0.73881400	-0.19212100
H	-0.41144700	-1.63136000	-0.23130300
C	-0.45481300	0.51119600	-0.21925700
C	-1.27994600	1.64387200	-0.16859900
C	-2.65097700	1.52294700	-0.09025400
H	-7.69383300	-4.18154100	0.21778000
H	-5.23654000	-4.41372800	0.04642100
H	-7.30369200	0.08668700	0.21040700
H	-8.70213100	-1.89730100	0.29919100
H	0.62398600	0.61496100	-0.27969900
H	-0.84083100	2.63694100	-0.19042900
H	-3.25580000	2.42160200	-0.05209100
C	-5.37838200	1.42388000	0.06815100
C	-5.78920100	2.00435000	-1.12510300
C	-5.63683900	2.00962400	1.30103700
C	-6.48092800	3.21132600	-1.07857700
H	-5.56704600	1.51289700	-2.07003300
C	-6.32896100	3.21668200	1.33602600
H	-5.29792000	1.52243700	2.21296200
C	-6.74927500	3.81499000	0.14903900
H	-6.80992300	3.67987600	-2.00202000
H	-6.53967200	3.68964900	2.29124300
H	-7.28932600	4.75751000	0.18087000
N	-4.65757600	0.16924200	0.02493500
Se	-3.05631100	-2.61985200	-0.09517000

11. NMR Spectra for PC 3

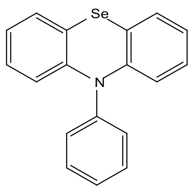

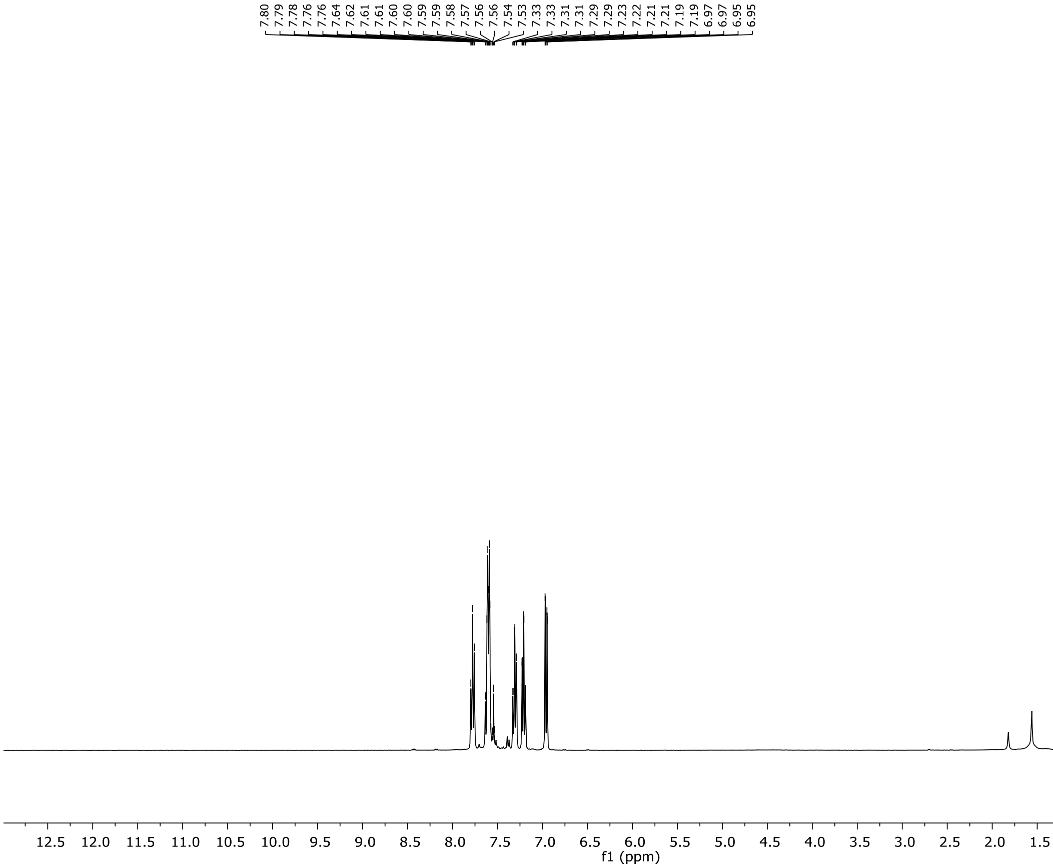

Figure S83. ^{1}H NMR Spectrum of PC **3** (400 MHz, $CDCl_3$).

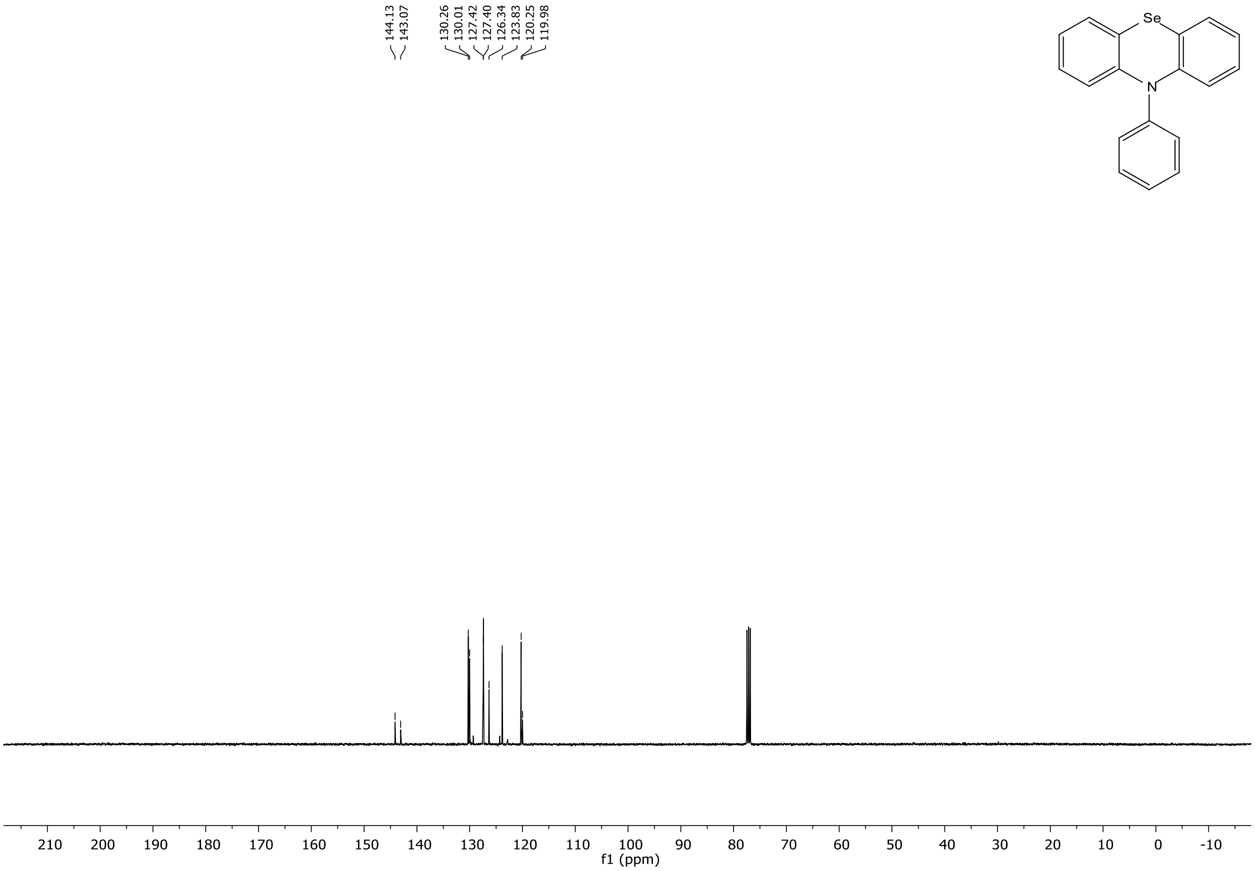

Figure S84. ^{13}C NMR Spectrum of PC **3** (101 MHz, $CDCl_3$).

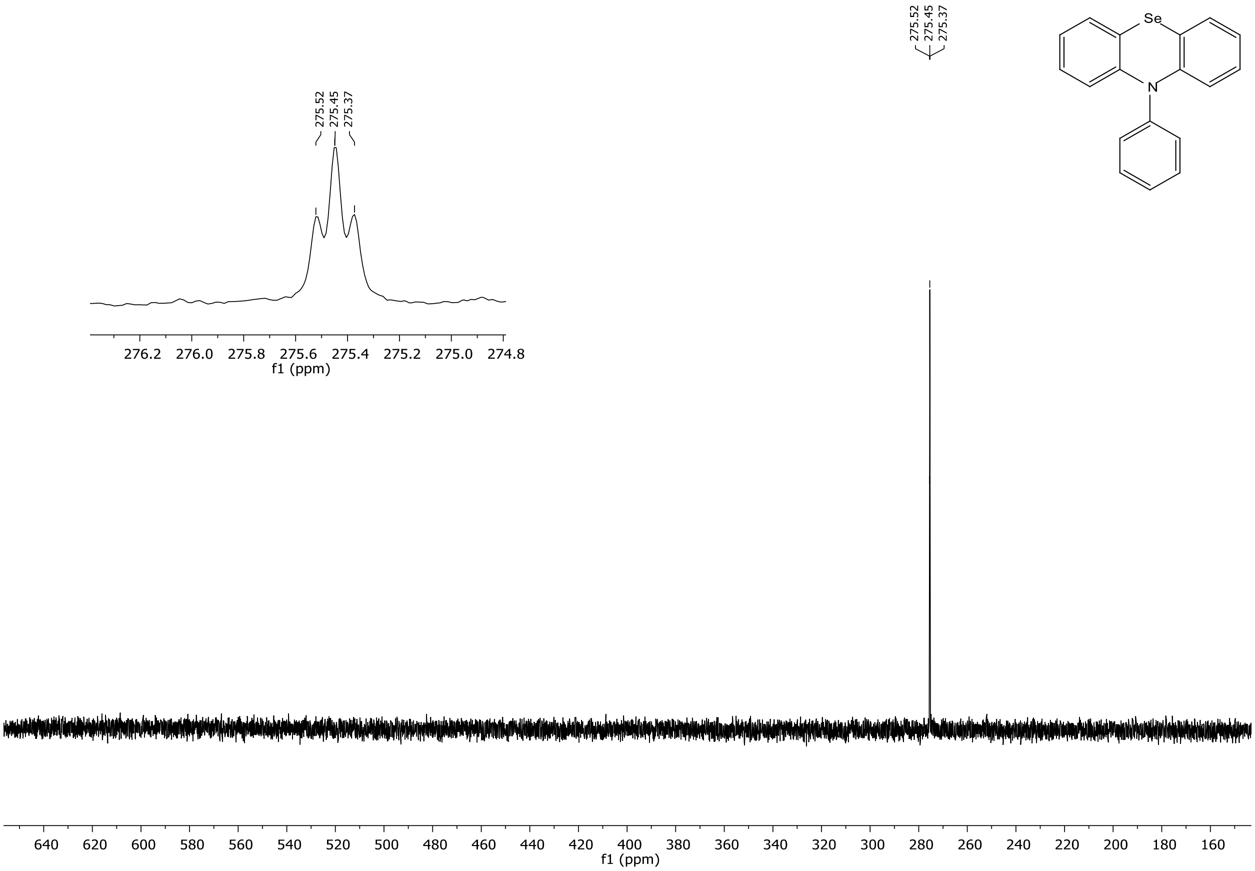

Figure S85. ^{77}Se NMR Spectrum of PC **3** (115 MHz, $CDCl_3$).

12. References

1. K. Targos; O. P. Williams; Z. K. Wickens, Unveiling Potent Photooxidation Behavior of Catalytic Photoreductants. *J. Am. Chem. Soc.* **2021**, *143*, 4125–4132.
2. G. M. Sheldrick, Program for Empirical Absorption Correction of Area Detector Data. *SADABS* **1996**.
3. G. M. Sheldrick, *SHELXTL*, v. 6.14; Bruker AXS: Madison, WI, 1999.
4. C. F. Macrae; I. Sovago; S. J. Cottrell; P. T. A. Galek; P. McCabe; E. Pidcock; M. Platings; G. P. Shields; J. S. Stevens; M. Towler; P. A. Wood, Mercury 4.0: From Visualization to Analysis, Design and Prediction. *J. Appl. Cryst.* **2020**, *53*, 226–235.
5. N. Liu; B. Wang; W. Chen; C. Liu; X. Wang; Y. Hu, A General Route for Synthesis of *N*-Aryl Phenoxazines *via* Copper(I)-Catalyzed *N*-, *N*-, and *O*-Arylations of 2-Aminophenols, *RSC Adv.* **2014**, *4*, 51133–51139.
6. C. L. Klein; J. M. Conrad III; S. A. Morris, Structure of *N*-Phenylphenothiazine, $C_{18}H_{13}NS$, *Acta Cryst.* **1985**, *C41*, 1202–1204.
7. R. R. Gagne; C. A. Koval; G. C. Lisensky, Ferrocene as an Internal Standard for Electrochemical Measurements. *Inorg. Chem.* **1980**, *19*, 2854–2855.
8. S. Trasatti, The Absolute Electrode Potential: An Explanatory Note (Recommendations 1986). *Pure Appl. Chem.* **1986**, *58*, 955–966.
9. M. J. Frisch; G. W. Trucks; H. B. Schlegel; G. E. Scuseria; M. A. Robb; J. R. Cheeseman; G. Scalmani; V. Barone; G. A. Petersson; H. Nakatsuji; X. Li; M. Caricato; A. V. Marenich; J. Bloino; B. G. Janesko; R. Gomperts; B. Mennucci; H. P. Hratchian; V. J. Ortiz; A. F. Izmaylov; J. L. Sonnenberg; D. Williams-Young; F. Ding; F. Lipparini; F. Egidi; J. Goings; B. Peng; A. Petrone; T. Henderson; D. Ranasinghe; V. G. Zakrzewski; J. Gao; N. Rega; G. Zheng; W. Liang; M. Hada; M. Ehara; K. Toyota; R. Fukuda; J. Hasegawa; M. Ishida; T. Nakajima; Y. Honda; O. Kitao; H. Nakai; T. Vreven; K. Throssell; J. A. Montgomery Jr.; J. E. Peralta; F. Ogliaro; M. J. Bearpark; J. J. Heyd; E. N. Brothers; K. N. Kudin; V. N. Staroverov; T. A. Keith; R. Kobayashi; J. Normand; K. Raghavachari; A. P. Rendell; J. C. Burant; S. S. Iyengar; J. Tomasi; M. Cossi; J. M. Millam; M. Klene; C. Adamo; R. Cammi; J. W. Ochterski; R. L. Martin; K. Morokuma; O. Farkas; J. B. Foresman; D. J. Fox, *Gaussian 16, Revision C.01* Gaussian, Inc., Wallingford CT, 2016.
10. H. He; P. Zapol; L. A. Curtiss, A Theoretical Study of CO_2 Anions on Anatase (101) Surface. *J. Phys. Chem. C,* **2010**, *114*, 21474–21481.
11. J. A. Tossell, Calculation of the Properties of Molecules in the Pyridine Catalyst System for the Photochemical Conversion of CO_2 to Methanol. *Comput. Theor. Chem.* **2011**, *977*, 123–127.
12. P. Winget; C. J. Cramer; D. G. Truhlar, Computation of Equilibrium Oxidation and Reduction Potentials for Reversible and Dissociative Electron-Transfer Reactions in Solution. *Theor. Chem. Acc.* **2004**, *112*, 217–227.
13. Y. Zhao; D. Truhlar, The M06 Suite of Density Functionals for Main Group Thermochemistry, Thermochemical Kinetics, Noncovalent Interactions, Excited States, and Transition Elements: Two New Functionals and Systematic Testing of Four M06-Class Functionals and 12 Other Functionals. *Theor. Chem. Acc.* **2008**, *120*, 215–241.
14. B. G. McCarthy; R. M. Pearson; C.-H. Lim; S. M. Sartor; N. H. Damrauer; G. M. Miyake, Structure–Property Relationships for Tailoring Phenoxazines as Reducing Photoredox Catalysts. *J. Am. Chem. Soc.* **2018**, *140*, 5088–5101.
15. F. Sun; R. Jin, DFT and TD-DFT Study on the Optical and Electronic Properties of Derivatives of 1,4-Bis(2-Substituted-1,3,4-Oxadiazole)Benzene. *Arab. J. Chem.* **2017**, *10*, S2988–S2993.
16. C.-H. Lim; M. D. Ryan; B. G. McCarthy; J. C. Theriot; S. M. Sartor; N. H. Damrauer; C. B. Musgrave; G. M. Miyake, Intramolecular Charge Transfer and Ion Pairing in *N,N*-Diaryl

Dihydrophenazine Photoredox Catalysts for Efficient Organocatalyzed Atom Transfer Radical Polymerization. *J. Am. Chem. Soc.* **2017**, *139*, 348–355.
17. R. M. Pearson; C.-H. Lim; B. G. McCarthy; C. B. Musgrave; G. M. Miyake, Organocatalyzed Atom Transfer Radical Polymerization using *N*-Aryl Phenoxazines as Photoredox Catalysts. *J. Am. Chem. Soc.* **2016**, *138*, 11399–11407.
18. N. J. Treat; H. Sprafke; J. W. Kramer; P. G. Clark; B. E. Barton; J. R. de Alaniz; B. P. Fors; C. J. Hawker, Metal-Free Atom Transfer Radical Polymerization. *J. Am. Chem. Soc.* **2014**, *136*, 16096–16101.
